# Fenvalerate exposure induces AKT/AMPK-dependent alterations in glucose metabolism in hepatoma cells

**DOI:** 10.3389/fphar.2025.1540567

**Published:** 2025-02-25

**Authors:** Lu Sun, Zheng-Guo Cui, Qianwen Feng, Jibran Sualeh Muhammad, Yu-Jie Jin, Songji Zhao, Lingqi Zhou, Cheng-AI Wu

**Affiliations:** ^1^ Department of Pediatric Cardiology, Heart Center, Guangzhou Women and Children’s Medical Center, Guangzhou Medical University, Guangzhou, China; ^2^ Department of Environmental Health, University of Fukui School of Medical Sciences, Fukui, Japan; ^3^ Biocytogen Phaceuticals, Daxing Bio-Medicine Industry Park, Beijing, China; ^4^ Department of Biomedical Sciences, College of Medicine and Health, University of Birmingham, Birmingham, United Kingdom; ^5^ Department of General Practice, The First Hospital of Hebei Medical University, Shijiazhuang, Hebei, China; ^6^ Advanced Clinical Research Center, Fukushima Global Medical Science Center, Fukushima Medical University, Fukushima, Japan; ^7^ Department of Molecular Orthopedics, Beijing Research Institute of Traumatology and Orthopedics, Beijing Jishuitan Hospital, Beijing, China

**Keywords:** fenvalerate, warburg-like effect, cancer cell metabolism, reactive oxygen species, insulin receptor

## Abstract

**Background:**

Fenvalerate (Fen) is a synthetic pyrethroid insecticide significantly associated with an increased risk of type 2 diabetes. Tumor cells exhibit a shift in glucose metabolism, known as the Warburg effect. Accordingly, we aimed to elucidate whether Fen interferes with insulin signaling and affects hepatoma cell metabolism.

**Methods:**

The cells were subjected to Fen to assess glucose uptake, acidification, oxygen consumption, and ATP production. ROS generation, mitochondrial membrane potentials, and protein expression were evaluated by flow cytometry, immunofluorescence microscopy, and western blot analyses.

**Results:**

Our results demonstrated that Fen promotes glucose uptake, lactate production, and ATP generation in various cancer cells. Moreover, Fen enhanced insulin receptor phosphorylation and upregulated p-AKT/p-AMPK expression. Fen enhanced insulin receptor sensitivity and endocytosis via reactive oxygen species generation rather than the PP2B pathway. Additionally, the antioxidants N-acetyl-L-cysteine and ascorbic acid reversed the Fen-induced increase in glycolysis. Finally, chronic Fen exposure protected hepatoma cells against metformin-induced cell death via the AKT/AMPK pathway.

**Conclusion:**

These findings raise concerns regarding the safety of Fen and its potential role in altering cancer cell metabolism, affecting insulin signaling and treating drug resistance, thereby necessitating further research.

## 1 Introduction

Liver cancer is one of the top five malignancies, killing approximately seven million people annually (Zhong et al., 2018). Due to the unclear early clinical symptoms of liver cancer, many patients are diagnosed after tumor cells have already spread to nearby lymph nodes or other organs. Several studies have described a strong link between hepatic tumorigenesis and obesity, diabetes, and metabolic liver diseases (Singh and Stricker, 2019; Guo et al., 2023). Metabolic comorbidities that impair glucose metabolism enhance the progression of liver cancer because the liver plays a major role in carbohydrate, amino acid, and lipid metabolism (Chen et al., 2018). In mammals, glycolytic breakdown of glucose produces pyruvate under aerobic conditions or lactate under anaerobic conditions. The “Evolutionary game theory” states that cancer cells use extra carbon sources from glucose consumption to accelerate the anabolic processes (Liberti and Locasale, 2016). In hepatoma cells, the glucose uptake rate and lactate production increase dramatically, even in the presence of oxygen (Zhong et al., 2018). Increased glycolysis, termed the “Warburg-like effect,” provides anabolic precursors and raw materials for ATP biosynthesis and reduces intracellular reactive oxygen species (ROS) production (Altman et al., 2016). Therefore, these processes are conducive to cancer cell growth and survival (Liberti and Locasale, 2016).

Fenvalerate (Fen) is a widely used agricultural and residential synthetic pyrethroid insecticide. It is a low molecular weight, oxygen-stable compound detected in crops, soil, water, sediments, and indoor environments and may enter the human body via the food chain (Cui et al., 2018). According to an *in vivo* study, it is rapidly absorbed and extensively distributed throughout the body due to its lipophilicity (Han et al., 2017). Chronic pyrethroid exposure causes chronic diseases of the immune, cardiovascular, and genetic systems (Amjad et al., 2020). Previous studies have revealed that Fen induces apoptosis in normal tissues but resists apoptosis in cancer cells (Cui et al., 2018; Zhao et al., 2011; Qiu et al., 2019). Moreover, Fen can induce hepatic lesions (Qiu et al., 2019). However, the effect of Fen on liver cancer remains unknown.

Mitogen activated protein kinase (MAPK) family members can affect cell proliferation, differentiation, survival and migration, and play a central role in human tumors. ERK is an important member of MAPK family, and some ERK kinase inhibitors have entered clinical trials (Drosten and Barbacid, 2020; Wagner and Nebreda, 2009). AKT is also overexpressed or activated in many tumors, including ovarian, lung, and pancreatic cancers. Many studies have selected AKT as a target for tumor prevention and treatment (Song et al., 2019). The use of PP2B (also known as calcineurin) inhibitors as immunosuppressants is associated with a high risk of type 2 diabetes (Duan and Cobb, 2010). In addition, PP2B inhibitors directly and indirectly affect glucose homeostasis in adipose tissue, skeletal muscle, pancreas, brain, and liver (Chakkera et al., 2017). Fenvalerate is a potent and specific inhibitor of PP2B, and non-pyrethroid inhibitors of PP2B include cyclosporine A and FK506 (Petr et al., 2013).

Previous studies have demonstrated that certain pyrethroids, including permethrin and deltamethrin, alter the glucose metabolism in mice (Xiao, 2017). Additionally, pyrethroid exposure is positively associated with the risk of diabetes, with Fen being the most important independent contributor (Jia et al., 2022). However, few studies have addressed the mechanism by which Fen regulates glucose metabolism. Besides, the impact of Fen on cancers remains uncertain. This study is the first to elucidate the potential hazards of Fen exposure by regulating energy production, including the molecular mechanism by which it may alter glucose metabolism in tumor cells.

## 2 Materials and methods

### 2.1 Reagents

Okadaic acid, deltamethrin, DMEM (Low Glucose) mediums, and cyclosporin A (Wako Pure Chemical Industries Ltd., Osaka, Japan), Antimycin A (Enzo Life Sciences, Farmingdale, NY, United States), Fen, LY294002, U0126, bpV (HOpic), c-Jun *N*-terminal kinase (JNK) inhibitor VIII, and metformin were purchased from Sigma-Aldrich Corp. (St. Louis, MO, United States). Other reagents were obtained from Tokyo Chemical Industry Co. Ltd. (Tokyo, Japan). Fenvalerate was dissolved in dimethyl sulfoxide (DMSO) prior to addition to culture medium to achieve various treatment concentrations. DMSO concentration in culture medium including control and fenvalerate treatment groups were 0.02%, as previously described (Cui et al., 2018).

### 2.2 Cell culture

HepG2, MCF-7, and A549 cells were obtained from the Japan Cancer Research Resources Bank (Tokyo, Japan). Cells were grown in Dulbecco’s Modified Eagle’s medium (DMEM) containing 10% fetal bovine serum (FBS) without any antibiotics and maintained at 37°C in a humidified incubator with 5% CO_2_. The medium was changed every 2–3 days. When the cells reached 75%–85% confluence, they were harvested using 0.05% trypsin and were sub-cultured into different plates according to the requirements of each experiment. The cells were allowed to grow freely overnight before treatment, as previously described (Li et al., 2019).

### 2.3 ATP luminescence assay

Intracellular ATP was measured using an ATP assay kit (Toyo Ink Inc., Tokyo, Japan) according to the manufacturer’s protocol. Briefly, cells were seeded in a 96-well culture plate at a density of 2.5 × 10^4^ cells/well and were pretreated with chemicals at various concentrations for 1 h. Then, 100 μL of the kit reagent was added to each well and incubated for 10 min at room temperature. Luminescence was detected using the Filter Max F5 plate reader (Molecular Devices, San Jose, CA, United States).

### 2.4 Extracellular acidification and oxygen consumption assays

Extracellular acidification was monitored using a glycolysis assay kit, and oxygen consumption was monitored using an extracellular oxygen consumption assay kit (Abcam, Cambridge, MA, United States). The experiments were performed according to the manufacturer’s instructions.

### 2.5 Intra and extracellular glucose measurement assays

Glucose levels were measured in cell culture medium using a glucose (GO) assay kit (Sigma-Aldrich Corp., St. Louis, MO, United States). Briefly, 50 μL of culture medium was combined with 950 μL of assay reagent at 37°C for 30 min. The absorbance was measured at 540 nm using a Filter Max F5 plate reader (Molecular Devices, San Jose, CA, United States). Cellular glucose uptake was measured using the glucose uptake assay kit (Biovision, Milpitas, CA, United States). Briefly, the cells were treated with 2-NBDG and a glucose uptake enhancer for 30 min at 37°C. Then, the cells were analyzed for fluorescence using flow cytometry (FACS Canto II; Beckman-Coulter, Miami, FL, United States).

### 2.6 Cell cytotoxicity assay

The cell counting kit-8 (CCK-8) (Dojindo Laboratories, Kumamoto, Japan) was used to determine cancer cell cytotoxicity. After treatment, cancer cells were plated in 96-well plates at a density of 5 × 10^3^ cells/well and incubated at 37°C with 5% CO_2_ for 24 h. After adding 10 μL of the CCK-8 solution, the cells were incubated for another 2 h. The absorbance was measured at 450 nm using a plate reader (Filter Max F5; Molecular Devices, San Jose, CA, United States). A lactate dehydrogenase (LDH) cytotoxicity detection kit (Takara Bio, Shiga, Japan) was used according to the manufacturer’s instructions.

### 2.7 Intracellular ROS assay

Intracellular ROS levels were detected using flow cytometry with dihydroethidium (DHE) and 2,7-dichlorodihydrofluorescein diacetate (DCFH) (Molecular Probes, Eugene, OR, United States). DHE in the cytosol fluoresces blue until it is oxidized by superoxide to 2-hydroxyethidium, which then intercalates within cellular DNA and stains the nucleus bright fluorescent red. The cells were incubated with 4 μM DHE or 20 μM DCFH for 30 min at 37°C and washed twice with PBS. Relative ROS generation was analyzed using flow cytometry (DHE: excitation at 488 nm and emission at 575 nm; DCFH: excitation at 488 nm and emission at 515 nm).

### 2.8 Measurement of mitochondrial membrane potentials

Tetramethylrhodamine, methyl ester, perchlorate (TMRM) was used to stain mitochondria. After treatment, the cancer cells were harvested, and TMRM (10 nM) (Molecular Probes, Eugene, OR, United States) was added to 1 mL of 1% FBS in PBS for 30 min at 37°C. The percentage of cells with loss in mitochondrial membrane potential (MMP) was analyzed using flow cytometry gated on red TMRM fluorescence (wavelengths were 488 nm for excitation and 575 nm for emission).

### 2.9 Immunofluorescence microscopy

HepG2 cells (5 × 10^5^ cells/well) were plated on four-well chamber slides and treated with Fen for 24 h or left untreated as a negative control. Next, the mitochondria were stained using Mito-Tracker, and lysosomes and late endosomes were stained using Lyso-Tracker. The cells were fixed in 1% formaldehyde in PBS for 5 min. Permeabilization was achieved using 0.2% Triton-X 100 in PBS for 20 min. Then, the cells were blocked with 1% bovine serum albumin for 1 h. The blocked cells were incubated with anti-human IR-beta, LC-3A/B, or PHB2 primary antibodies (1:200 dilution) overnight at 4°C, followed by incubation with a secondary antibody (1:1000 dilution). Images were obtained using an LSM780 confocal microscope (Zeiss).

### 2.10 Western blot analyses

After cell culture treatment, cells were washed three times with PBS, and total proteins were extracted using RIPA lysis buffer or IP buffer containing protease and phosphatase inhibitors. Western blot analysis was performed for protein expression as previously described (Sun et al., 2018). The phospho-IR (p-IR), IR-beta, PTEN, and beta-actin (β-actin) primary antibodies were purchased from Santa Cruz Biotechnology, Inc. (Santa Cruz, CA). Other primary and secondary antibodies were purchased from Cell Signaling Technology, Inc. (Beverly, MA, United States). Supplementary Table S1 shown the detail information of all antibodies. All images were quantified using ImageJ software (National Institute of Mental Health, Bethesda, MA, United States).

### 2.11 Statistics

All experiments were performed at least three times, with at least three replicate wells. Three independent experiments were performed for Western blotting, with a representative image. Data are presented as means ± standard deviation. One-way or two-way ANOVA with Dunnett’s test was performed to analyze multiple comparisons. Statistical significance was set at *P* < 0.05. Analyses were conducted using the GraphPad PRISM software version 5.

## 3 Results

### 3.1 Fen promotes aerobic glycolysis by increasing glucose uptake, lactate production, and ATP generation in cancer cell lines

**FIGURE 1:**
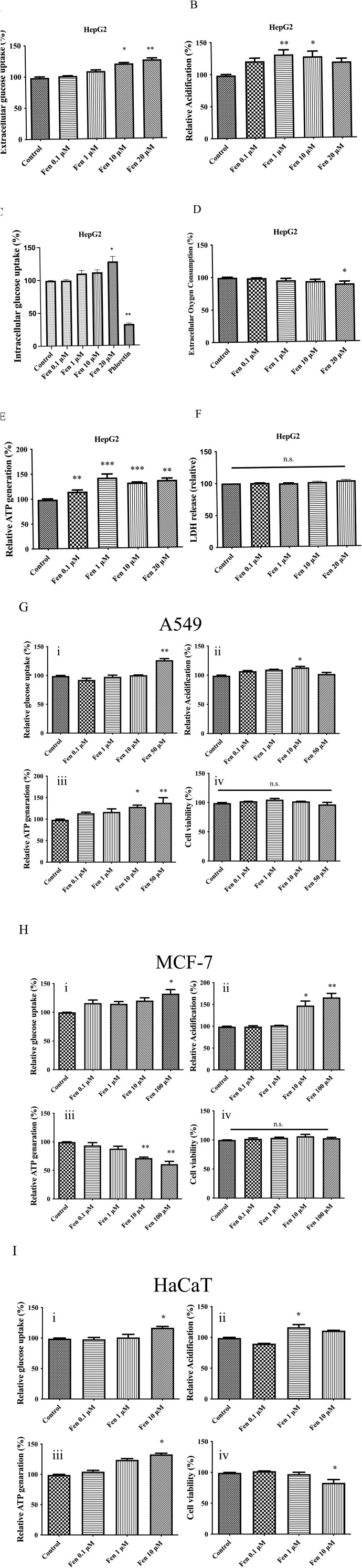
Fen enhances glucose uptake, lactate production, and ATP generation in several cell lines. **(A)** In HepG2 cells, extracellular glucose uptake was measured with the glucose (GO) assay kit. **(B)** HepG2 cells were treated with Fen for 1 h, and glycolysis was measured using a glycolysis assay kit. **(C)** HepG2 cell glucose uptake was measured by 2-NBDG uptake and flow cytometry. **(D)** HepG2 cells were incubated with the indicated Fen concentrations for 1 h, and extracellular oxygen consumption was measured using an extracellular oxygen consumption assay kit. **(E)** HepG2 cells were incubated with various Fen concentrations for 1 h. Cellular ATP levels were measured using a cell ATP assay reagent. **(F)** Cell culture medium was sampled, and cytotoxicity was determined using an LDH assay. In A549 cells **(G)**, MCF-7 cells **(H)**, and HaCaT cells **(I)**, extracellular glucose uptake was measured with glucose (GO) assay kit (i); glycolysis was determined with a glycolysis assay kit (ii); cellular ATP levels were measured with a cell ATP assay reagent (iii); viability was evaluated with CCK-8 reagent (iv). Statistical analysis was performed using one-way ANOVA (Dunnett’s test). *P < 0.05, **P < 0.01, and ***P < 0.001 vs. the control group.

We exposed HepG2 cells to increasing doses of Fen to elucidate their role in cancer metabolism. This pyrethroid increased the extracellular glucose uptake and lactate production and did not cause cell death (Figures 1A, B, F). To confirm this effect, we evaluated intracellular glucose uptake by treating cells with the fluorescent glucose analog, 2-NBDG, followed by flow cytometry. At 20 μM, Fen enhanced glucose uptake in HepG2 cells, whereas the negative control, the glucose transport inhibitor, phloretin, markedly decreased glucose uptake (Figure 1C). Furthermore, at 10–20 μM, Fen decreased extracellular oxygen consumption and promoted ATP production (Figures 1D, E). These results suggested that Fen may promote a Warburg-like effect in liver cancer cells.

We treated A549 lung adenocarcinoma and MCF-7 breast cancer cells with Fen to further evaluate their effects on other cancer cell lines. Fen promoted glycolysis and increased extracellular glucose uptake, lactate production, and ATP levels in A549 cells, as observed in HepG2 cells (Figure 1G). Fen treatment did not reduce MCF-7 cell viability but reduced ATP levels at a high concentration (100 µM), which may be because Fen demonstrated an estrogenic-like effect (Go et al., 1999). Instead, Fen sustained a Warburg-like effect in MCF-7 cells by increasing glucose uptake and lactate generation (Figure 1H).

We treated human immortalized keratinocytes (HaCaT) and 3T3-Swiss mouse fibroblast cells with Fen to investigate whether it induced a Warburg-like effect in non-cancer cells. The HaCaT cells responded to Fen treatment in essentially the same way as cancer cells. It enhanced glucose uptake in a dose-dependent manner. In HaCaT cells, Fen substantially increased lactate generation and ATP production at one or 10 μM (Figure 1I). The 3T3-Swiss cells also observed a similar trend of increasing glucose uptake and ATP production but did not alter lactate production (Supplementary Figure S1). Consistent with previous research, a low dose of Fen (10 μM) suppressed non-cancer cell viability, while cancer cells were unaffected (Figure 1G). Therefore, Fen exhibited a stronger impact on cancer cell metabolism than on non-cancer cell metabolism.

### 3.2 Fen-enhanced insulin receptor phosphorylation and upregulated p-AKT/p-AMPK expression

**FIGURE 2:**
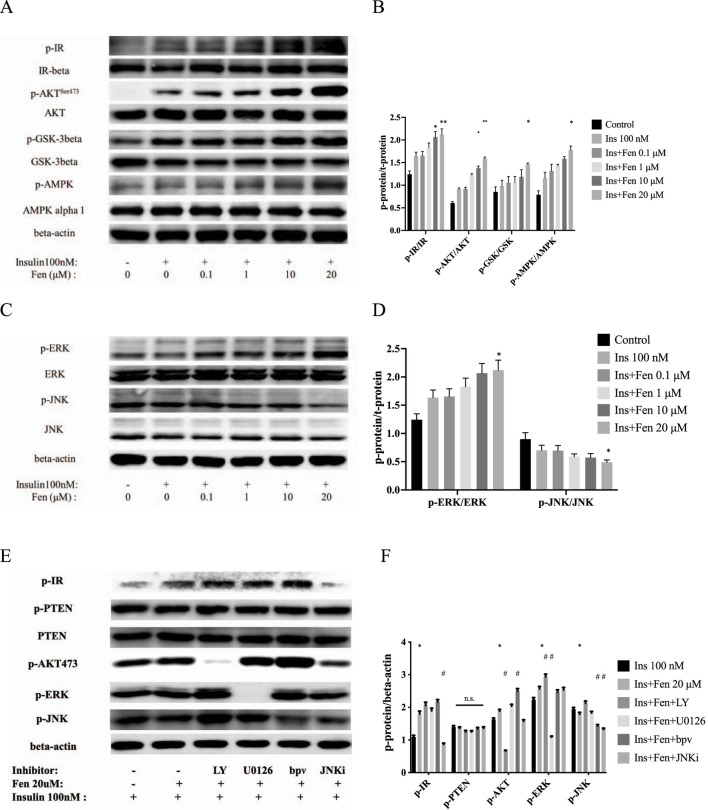
Fen enhanced insulin receptor phosphorylation and upregulated p-AKT/p-AMPK expression. **(A)** After 24 h of Fen pretreatment, followed by stimulation with 100 nM insulin for 10 min, phosphorylated and total IR, AKT, GSK-3-β, and AMPK levels were analyzed using Western blotting. **(C)** Expression of phosphorylated and total forms of ERK and JNK were analyzed using Western blotting. **(E)** HepG2 cells were pretreated with each inhibitor for 1 h before incubation with 20 µM Fen and stimulated with 100 nM insulin for 10 min. The expression levels of p-IR, p-PTEN, PTEN, p-AKT, p-ERK, and p-JNK were analyzed using Western blotting; β-actin was used as the loading control. Average values of the ratio of phosphorylated to total proteins **(B, D)** or beta-actin **(F)** were quantified in each group. Statistical analysis was performed using a two-way ANOVA. *P < 0.05 and **P < 0.01 vs. the control group. #*P* < 0.05 and ##*P* < 0.01 reagent pretreatment group vs. the 20 μM Fen combined group.

Insulin is the principal hormone responsible for cellular glucose metabolism. We examined the effects of Fen on the insulin receptor pathway using Western blotting to determine whether it increases glucose uptake in liver cancer cells by modulating insulin signaling. However, regarding insulin receptor and hepatokine expression, HepG2 cells appeared closer to the *in vivo* situation than immortalized human liver cells (Sefried et al., 2018). Pretreatment with Fen for 24 h, followed by stimulation with 100 nM insulin for 10 min, increased phosphorylation of the insulin receptor p-IR and its downstream factor p-AKT dose-dependently without altering IR-beta or AKT expression (Figures 2A, B). Furthermore, Fen upregulated p-AMPK^Ser485/491^ and GSK-3-beta^Ser9^ phosphorylation without changing total GSK-3-β and AMPK expression (Figures 2A, B). Additionally, Fen upregulated p-IR, p-AKT, and p-AMPK in mouse myoblast (C2C12) cells (Supplementary Figure S2A). These results suggest that Fen enhances the insulin signaling pathway in liver and myoblast cells.

The insulin signaling pathway mediates the growth and mitogenic effects of the PI3K-AKT and Ras/MAPK pathways (Hopkins et al., 2018). Consistently, 20 µM Fen combined with insulin increased the phosphorylation of the extracellular signal-regulated kinase (p-ERK) and decreased the phosphorylation of JNK (Figures 2C, D). To further examine the role of Fen in the PI3K-AKT and MAPK pathways, we pretreated HepG2 cells with different inhibitors for 1 h before incubation with 20 µM Fen, followed by stimulation with 100 nM insulin for 10 min. The PI3K-AKT inhibitor LY294002 and ERK inhibitor U0126 (20 µM) inhibited the expression of p-AKT and p-ERK, respectively, but did not modulate p-IR levels. The PTEN inhibitor bpV (HOpic) (5 µM) did not modulate p-PTEN or PTEN expression, and the increased p-AKT expression indicated that the inhibitor had worked. Furthermore, JNK inhibitor VIII (JNKi; 20 µM) did not upregulate p-IR expression (Figures 2E, F). Overall, these results suggested that Fen promoted glucose metabolism by increasing phosphorylation of insulin receptors and activated its downstream PI3K-AKT pathway in HepG2 cells.

### 3.3 Fen decreased IRS1 expression and promoted insulin receptor endocytosis

**FIGURE 3:**
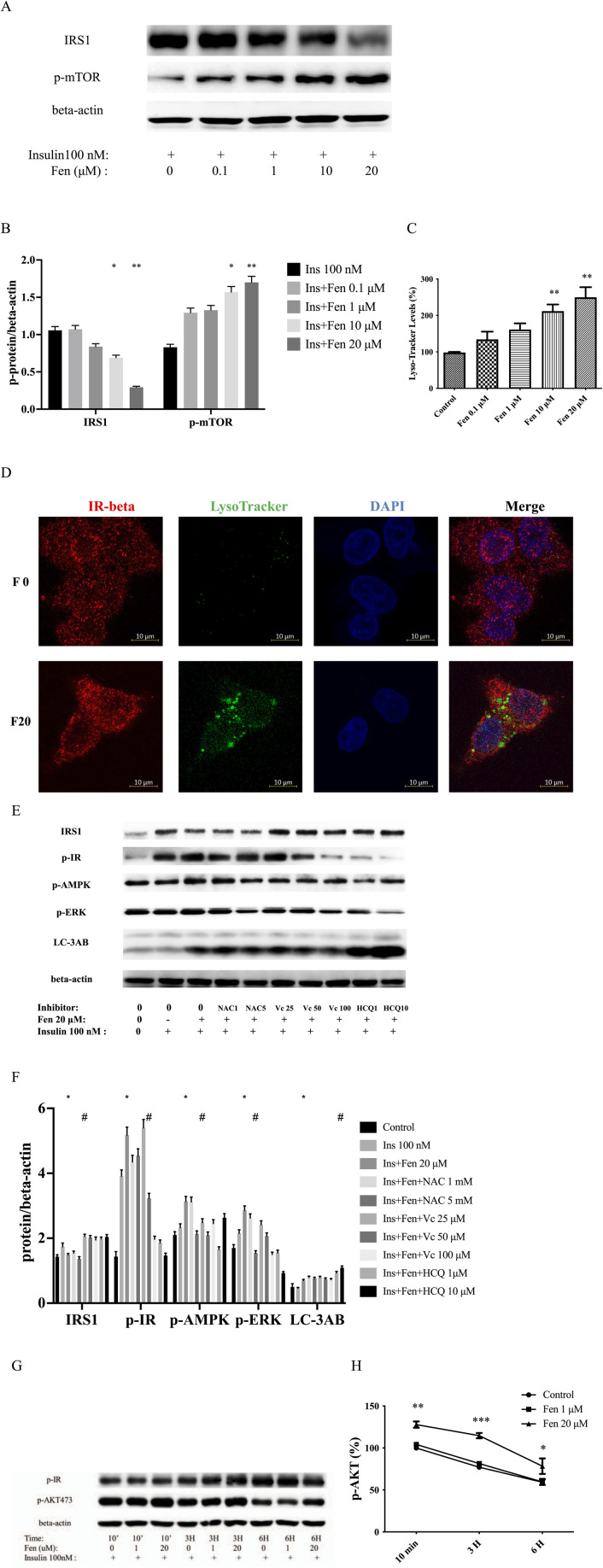
Fen decreased IRS1 expression and promoted insulin receptor endocytosis. HepG2 cells were pretreated with Fen for 24 h and stimulated with 100 nM insulin for 10 min. **(A)** Expression levels of IRS1 and p-mTOR were analyzed using western blotting. **(C)** Lyso-Tracker level was measured using flow cytometry with Lyso-Tracker staining. **(D)** Co-expressed IR-β and Lyso-tracker were stained with anti-IR-β (red), Lyso-Tracker staining (green), and DAPI (blue). Scale bar = 10 µm. Control or pretreatment with each inhibitor for 1 h before incubation with 20 μM Fen for 24 h, followed by stimulation with 100 nM insulin for 10 min. **(E)** The IRS1, p-IR, p-AMPK, p-ERK, and LC-3AB expression levels were analyzed using a western blot. **(G)** Pretreatment with Fen for 24 h followed by stimulation with 100 nM insulin for different times. The p-IR and p-AKT expression levels were analyzed using a western blot. Average values of the ratio of phosphorylated to beta-actin **(B,F,H)** were quantified in each group. **(B,F,H)** were performed using a two-way ANOVA. *P < 0.05 and **P < 0.01 vs. the control group. #*P* < 0.05 reagent pretreatment group vs. the 20 μM Fen combined group. Other statistical analysis was performed using one-way ANOVA (Dunnett’est). *P < 0.05, **P <0.01, and ***P < 0.001 vs. the control group.

Insulin binding to its receptor tyrosine kinases (RTKs) increased its concentration in vesicles, which was regulated by AMPK (Bergeron et al., 2016; Ray et al., 2023). Initiation of insulin receptor endocytosis required mTOR-dependent IRS-1 degradation (Yoneyama et al., 2018). Our results revealed that the combination of Fen and insulin induced the activation of p-mTOR and p-AMPK, leading to IRS1 degradation (Figures 2A, B, 3A). Furthermore, Fen (20 µM) promoted insulin receptor endocytosis, as manifested by the increased number of vesicles co-expressing IR-β and Lyso-Tracker (Figures 3C, D). The ROS generation may participate in this process. Fen-induced downregulation of IRS1 and upregulation of p-AMPK Fen could be partially recovered by co-treatment with the H_2_O_2_ scavenger ascorbic acid (Figures 3E, F). We used the inhibitor hydroxychloroquine (HCQ) to block autophagy flux. Our data exhibited that Fen combined with HCQ induced a greater LC-3II accumulation (Figure 3F). Therefore, Fen could not completely inhibit autophagy flux. Fen combined with insulin realized higher p-AKT activity than insulin alone in a time-dependent manner. This difference was particularly evident at 3 h (Figures 3G, H). Endosome acidification dissociated insulin from its receptor. Therefore, the Fen pretreatment group presented less p-IR protein than the insulin-only group after 6 h (Figure 3H). The Fen combined group also sustained higher p-AKT expression than the insulin-only treatment. In hepatoma cells, Fen-enhanced insulin-stimulated receptor endocytosis may allow them to adapt to the ambient environment.

### 3.4 Fen-driven ROS generation sustained a warburg-like effect, independent of the PP2B pathway

**FIGURE 4:**
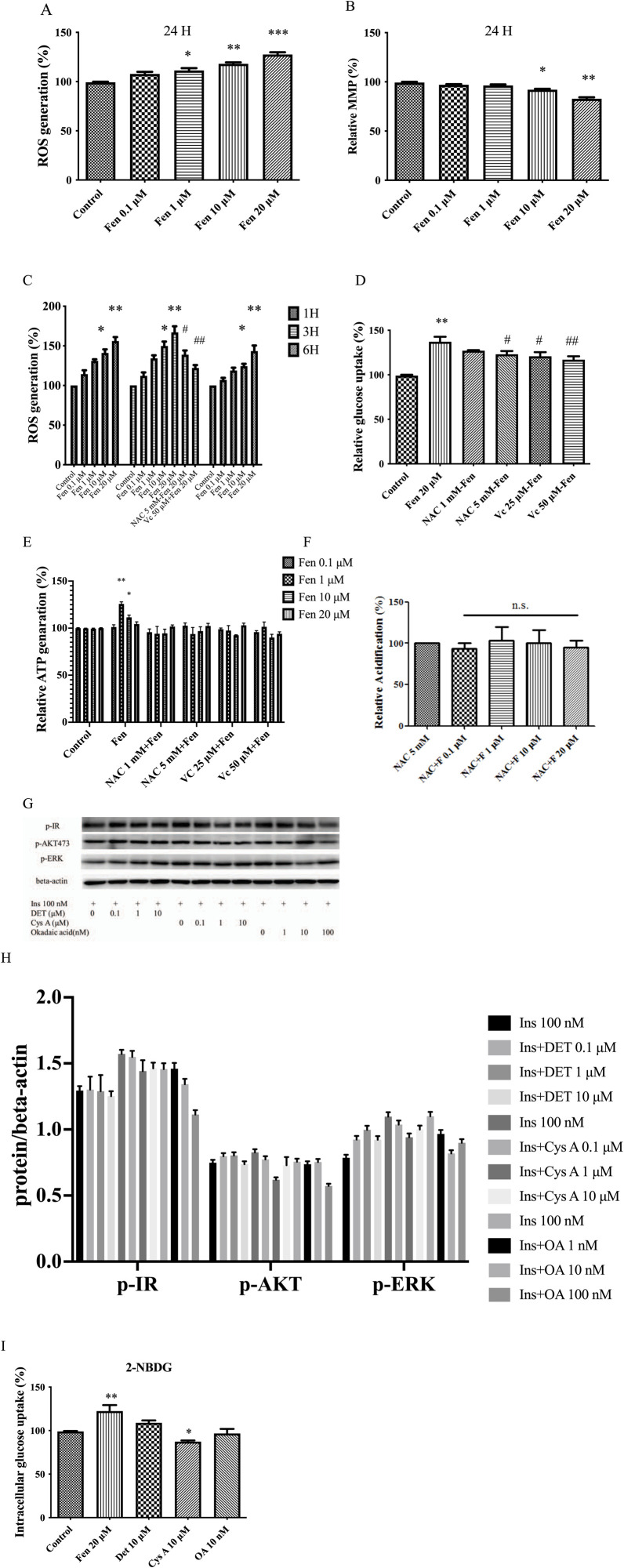
Fen-driven ROS generation induced mitochondrial dysfunction and sustained the Warburg-like effect. **(A)** Intracellular O_2_ generation was measured using flow cytometry with DHE staining. **(B)** MMP was determined using flow cytometry with TMRM staining. **(C)** HepG2 cells were either untreated or pretreated with ROS scavengers for 1 h before incubation with Fen for the indicated times. **(D)** After 24 h Fen treatment, extracellular glucose uptake was measured using a glucose (GO) assay kit. **(E)** Cellular ATP levels were measured using a cell ATP assay reagent. **(F)** Glycolysis was determined using a glycolysis assay kit. **(G)** After deltamethrin or cyclosporin A or okadaic acid pretreatment for 24 h, followed by stimulation with 100 nM insulin for 10 min, the p-IR, p-AKT, and p-ERK expression levels were analyzed using Western blot; β-actin was the loading control. **(I)** HepG2 cell glucose uptake was assessed by analyzing 2-NBDG uptake using flow cytometry. Average values of the ratio of phosphorylated to beta-actin **(H)** was quantified in each group. **(C, E, G)** were performed using a two-way ANOVA. *P < 0.05 and **P < 0.01 vs. the control group. #*P* < 0.05 and ##*P* < 0.01 reagent pretreatment group vs. the 20 μM Fen combined group. Other analysis was performed using a one-way ANOVA (Dunnett’s test). *P < 0.05, **P < 0.01, and ***P < 0.001 vs. the control group; #*P* < 0.05 and ##*P* < 0.01 for the pretreatment group vs. the 20 μM Fen combined group; n.s = non-significant .

As we established, Fen promoted glucose metabolism via the insulin receptor pathway. Previous studies demonstrated that pyrethroids induce ROS generation, modulating glucose uptake (Qiu et al., 2019; Loh et al., 2009). In our data, ROS generation was first examined using DHE flow cytometry analysis at various concentrations (Figure 4A). DCFH staining discloses similar results (Supplementary Figure S2B). ROS generation and glycolysis were closely related to mitochondrial function (Cui et al., 2018). Next, we evaluated the mitochondrial function and found that MMP loss increased in a dose-dependent manner at 24 h (Figure 4B). Dose-dependent Fen-induced ROS generation was partially inhibited by the ROS scavengers N-acetylcysteine (NAC) and ascorbic acid (Vc) (Figure 4C). This finding was corroborated by co-treatment with NAC or ascorbic acid, significantly abrogating Fen-induced glucose uptake (Figure 4D; Supplementary Figure S2C), ATP generation (Figure 4E), and lactate production (Figure 4F).

Furthermore, Fen is a potent inhibitor of protein phosphatase 2B (PP2B, which also known as calcineurin), an important factor involved in glucose uptake (Fonseca et al., 2018; Chakkera et al., 2017). We attempted to determine whether the PP2B inhibitors (deltamethrin and cyclosporine A) and the PP1-PP2A inhibitor (okadaic acid) also regulate glucose uptake. Western blotting and intracellular glucose uptake measurements revealed that deltamethrin, cyclosporine A, and okadaic acid failed to upregulate p-IR or increase glucose uptake in liver cancer cells (Figures 4G–I). Fen-driven ROS generation may sustain Warburg-like effects and promote glucose metabolism. PP2B inhibitors may not be involved in Fen-enhanced glucose consumption in liver cancer cells.

### 3.5 Chronic low-dose fen exposure protects HepG2 cells against metformin-induced cell death

**FIGURE 5:**
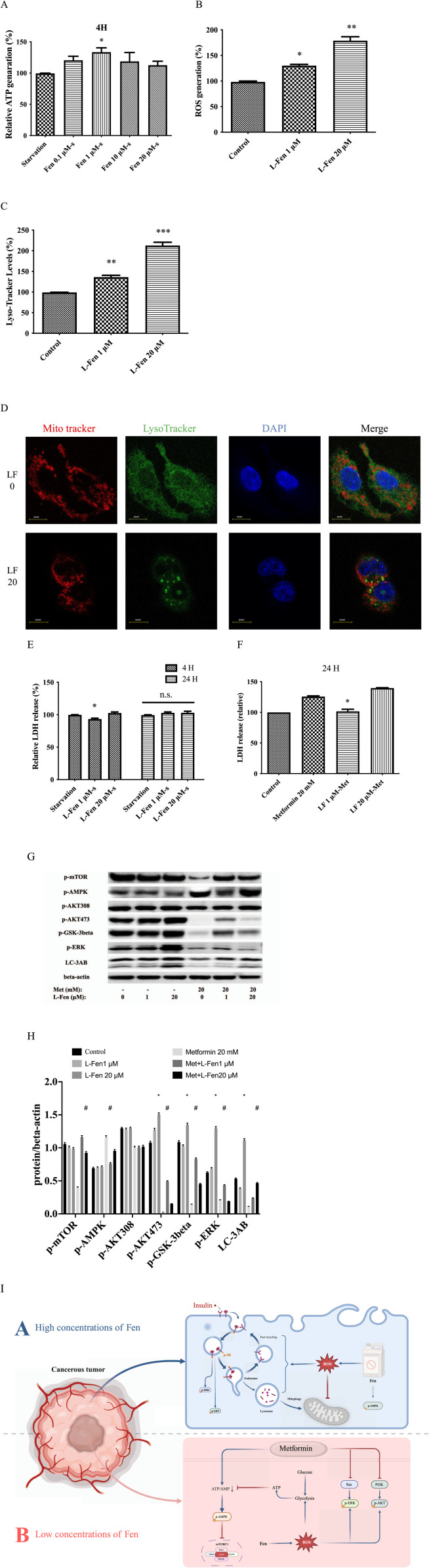
Chronic Fen exposure protected HepG2 cells against metformin-induced cell death. **(A)** Chronic Fen-HepG2 cell cultures were treated with 20 mM metformin for 24 h. Cell culture medium were sampled, and cytotoxicity was determined using an LDH assay. **(B)** Expression levels of p-mTOR, p-AMPK, p-AKT^Thr308^, p-AKT^Ser473^, p-GSK-3-β, p-ERK, and LC-3AB were analyzed using Western blotting; β-actin was used as the loading control. **(D)** Co-expressed Mito-Tracker and Lyso-Tracker were stained with Mito-Tracker staining (red), Lyso-Tracker staining (green), and DAPI (blue). Scale bar = 10 µm. **(E)** In chronic Fen-treated HepG2 cell cultures, Lyso-Tracker levels were measured using flow cytometry with Lyso-Tracker staining. **(F)** Intracellular O_2_ generation was measured using flow cytometry with DHE staining. **(G)** Chronic Fen-treated HepG2 cell cultures were starved at different times. Cell culture medium were sampled, and cytotoxicity was determined using the LDH assay. **(H)** Cellular ATP levels were measured using a cell ATP assay reagent. **(I)** Schematic diagram depicting the Fen-sustained Warburg-like effect and enhanced glucose metabolism (Created in BioRender. Sun, L. (2025) https://BioRender.com/x86k997). Average values of the ratio of phosphorylated to beta-actin **(C)** was quantified in each group. **(C, G)** was performed using a two-way ANOVA. *P < 0.05 vs. the control or starvation group; #*P* < 0.05 for the pretreatment group vs. the metformin group. The other analysis was performed using a one-way ANOVA (Dunnett’s test). *P < 0.05, **P < 0.01, and ***P < 0.001 vs. the control group; n.s = non-significant .

Starvation has been proven to reduce age-related diseases, including cancer and diabetes (O'Flanagan et al., 2017). Our results demonstrated that starvation (DMEM, excluding serum and glucose) was too severe in the HepG2 cells. After 24 h, nearly all the cells were floating. After 4 h, 1 µM Fen can maintaining high ATP levels (Figure 5A). Therefore, we selected 1 µM Fen with the obviously ATP production under starvation and 20 µM Fen with significant glucose uptake for long-term culture. We cultured HepG2 cells with 0, one or 20 µM Fen for 3 months. In short, we refreshed the culture medium containing Fen or DMSO every 2 days and the cells were counted every 4 days to ensure consistent cell numbers. After 3 months, cells cultured in Fen produced more ROS generation and lysosomal acidification (Figures 5B, C). The co-expression of acidic vesicles and Mito-Tracker suggested that high concentration of Fen maybe promote mitophagy, thereby affecting ATP production (Figure 5D; Supplementary Figure S3B). Consistent with before, after 4 h of starvation, chronic 1 µM Fen could delay cancer cell death, while chronic 20 µM Fen did not have this effect (Figure 5E). Metformin, also widely used in metabolic diseases, inhibited lysosomal proton pumps v-ATPase, which was the central nodes of AMPK activation after starvation (Chen et al., 2013; Ma et al., 2022). The lower dose of Fen (1 µM) improved HepG2 cell resistance to metformin-induced cell death (Figure 5F). Although chronic 20 µM Fen did not rescue metformin-induced cell death, it promoted HepG2 cell adhesion to the plates (Supplementary Figure S3A). Western blotting assays revealed that 1 µM Fen combined with 20 mM metformin significantly upregulated p-AKT^Ser473^ and downregulated p-AMPK compared to the 20 mM metformin group (Figure 5G). However, 20 mM metformin did not substantially downregulate p-AKT^Thr308^ (Figure 5H). These results indicated that chronic exposure to low doses of Fen (1 µM) could increase cancer cell viability by inhibiting p-AMPK expression and activating p-AKT. However, the high-dose group (20 µM) affected mitochondrial function (Figure 5H).

## 4 Discussion

The Warburg effect is a characteristic cancer cell property (Faubert et al., 2017). This study is the first to demonstrate that Fen treatment enhances cancer cell glucose uptake, lactate production, and ATP generation and decreases oxygen consumption in various cancer cell lines. Our data revealed that Fen promotes insulin receptor sensitivity, internalization, and p-AKT/p-AMPK expression. Fen-driven ROS generation sustained energy production via anaerobic glycolysis, indicating a Warburg-like effect. ROS scavengers inhibit this effect. Finally, we demonstrated that chronic Fen treatment induced stress resistance to metformin therapy via the AKT and AMPK pathways (Figure 5I).

Pyrethroid promoted glycolysis and reduced products of the TCA cycle (Guven et al., 2018). Moreover, previous *in vivo* studies demonstrated that pyrethroids, such as cismethrin, permethrin, and deltamethrin, significantly increase blood glucose and lactate levels in rats (Yang et al., 2018; Cremer and Seville, 1982). Similarly, a high dose of Fen decreased mouse serum glucose levels and increased LDH activity (Qiu et al., 2019). Previous studies suggested that the half-life of Fen in rodents is approximately 0.5–0.6 days (Shah, 2012). A cohort study displayed that Fen could still be detected in human serum with a median concentration of 1.16 μg/L. Moreover, pyrethroids were positively associated with the risk of incident diabetes, with serum Fen ranked as a top contributor (Jia et al., 2022). However, the molecular mechanisms by which Fen affects cellular metabolism and glucose uptake remain unclear. Our study is the first to reveal the impact of Fen on altering tumor cell metabolism, especially in the insulin signaling pathway.

Aerobic glycolysis is a general and essential mechanism for cancer cells to consume glucose under normoxin, and it is a preferred way to obtain ATP better than oxidative phosphorylation in tumor cells (Guo et al., 2023). Glycolysis is an incomplete energy-release process that generates less ROS than complete mitochondrial metabolism (aerobic respiration). Additionally, previous studies indicate that ROS generation is closely related to inflammation, protein kinase, metabolism, and insulin resistance (Loh et al., 2009; Di et al., 2011; Zhang et al., 2016). Accordingly, altered redox regulation adds complexity to the cancer phenotype (Karisch et al., 2011). In hepatoma cells, Fen promoted ROS generation, displayed a Warburg-like effect, and enhanced insulin receptor sensitivity (Figures 1A–E). In chronic exposure experiments, excessive autophagy induction and ROS generation did not ameliorate metformin-induced cell death (Figures 5E, F). Long-term exposure to 20 µM Fen may enhance mitophagy (Figure 5D; Supplementary Figure S3B). Therefore, we speculated that ROS produced by Fen may promote glucose uptake.

The protein tyrosine phosphatases (PTPs) family influences cell growth, differentiation, receptor endocytosis, mitotic cycles, and metabolic control (Gurzov et al., 2015), such as PTP1B preferentially dephosphorylates the insulin receptor β subunit in tandem with tyrosine residues (pY1162/1163) to downregulate insulin signaling (Dagnell et al., 2013). Protein phosphatase 2 B (PP2B) and PTPs contain an essential cysteine at the active site, forming a covalent intermediate with the phosphate substrate (Morrison et al., 2000). As a result, Fen, a PP2B inhibitor, may affect the PTP family. In our study, Deltamethrin, a pyrethroid insecticide, did not significantly increase glucose uptake in HepG2 cells and did not result in increased glucose uptake in mouse myoblast cells (Figure 4I; Supplementary Figure S2C). The differences in the effects of Fen and Deltamethrin may be explained by their relative cytotoxicity. Female mice fed 40.8 mg/kg bw/d Fen present with slightly increased alanine aminotransferase activity (Shah, 2012), while female rats fed 0.3 mg/kg bw/d deltamethrin exhibit disruption of hepatic function (Chargui et al., 2012). Furthermore, PP2B siRNA decreased the blood glucose levels in db/db mice (Wang et al., 2012). Conversely, the PP2B inducer decreased blood glucose in ob/ob mice (Lim et al., 2018). Consistent with these findings, our study found that the same PP2B inhibitor, Fen, promoted glucose uptake, while cyclosporine A inhibited glucose uptake in HepG2 cells (Figure 4I). Therefore, the PP2B/NFAT pathway has various effects on regulating glucose metabolism under different conditions and may not be involved in Fen-enhanced glucose uptake.

Endocytosis is the major internalization pathway for ligand-occupied RTKs (Goh and Sorkin, 2013). The endocytosis of activated insulin receptors concentrates them in endosomes and phosphorylates the insulin receptor substrates, which are recycled from the endosomes to the cell surface much faster than others (Goh and Sorkin, 2013). Moreover, the recycling of insulin-like growth factor receptors sustains higher activity of protein kinase B in endosomes (Balbis et al., 2000). Consistently, our data displayed that Fen combined with insulin promoted insulin receptor internalization (Figure 3D) and sustained higher p-AKT expression than insulin-only treatment (Figure 3H). Moreover, Fen activated duration of AKT and AMPK pathways via ROS generation in a dose-dependent manner (Figures 2A, 3E). AMPK and AKT have major effects on metabolic stress. AKT promotes energy production and cell proliferation and contributes to the occurrence and progression of tumors (He et al., 2021). When activated, AMPK promotes glucose uptake and glycolysis and facilitates antioxidant production (Zhao et al., 2017). The duration of AMPK activation is an important factor for hepatocellular cells to evade treatment, and tumor cells can promote drug resistance (Guo et al., 2023; Zhang et al., 2022; Han et al., 2023). Consistently, Fen promoted glucose uptake via AM^52^PK/AKT pathway. Moreover, the AMPK/AKT pathway is involved in rescuing metformin-induced cell death.

For decades, metformin has been the mainstay of therapy for diabetes mellitus (Pernicova and Korbonits, 2014). A large number of epidemiological and clinical studies have shown that metformin can prevent cancer and inhibit the progress of tumor growth and metastasis (Wu et al., 2023). The effects of metformin on hepatocellular carcinoma (HCC) cell death pathways include apoptosis, autophagy, ferroptosis and pyroptosis (Wu et al., 2023). As the most important target of metformin, AMPK not only induces apoptosis, but also promotes other types of cell death pathways. Metformin can also induce cell death through autophagy, as *in vitro* and *in vivo* models of liver cancer, the AMPK- mTOR axis is altered after metformin treatment. Metformin can promote ferroptosis in HCC by down regulating the expression of transcription factor 4. Furthermore, metformin induces pyroptosis in leptin receptor-defective hepatocytes via overactivation of the AMPK axis (Franco-Juarez et al., 2024). In addition, in other cancer cells, metformin can promote cell death through anoikis, necroptosis, senescence, mitotic catastrophes (Wu et al., 2023). In general, the outer layer of solid tumors is rich in nutrients, while the center is prone to cell death due to nutrient deprivation. The distribution of Fen in tumors also follows this rule. Low concentrations of Fen (1 μM) inhibit cell death caused by starvation or metformin through AMPK/AKT. However, high concentrations of Fen (20 μM) activate the insulin pathway more significantly and uptake more glucose. In addition, high concentrations of Fen can increase the adhesion of cells, and the invasion ability might be strengthened.

## 5 Conclusion

In this study, we demonstrated that Fen induces Warburg-like effect in cancer cells via ROS generation. Fen enhanced glucose metabolism and insulin receptor internalization via upregulated ROS generation. Moreover, chronic Fen treatment induces stress resistance to metformin therapy. Although Fen is widely used due to its comparatively low toxicity, it changes the metabolism of tumor cells, especially the insulin signaling pathway, and potentially affects chemotherapy resistance. In the future, we would elucidate it signaling pathways and the potential to predicted liver cancer.

## Data Availability

The original contributions presented in the study are included in the article/Supplementary Material, further inquiries can be directed to the corresponding authors.

## Glossary

Fen: Fenvalerate
ATP: Adenosine 5′-triphosphate
AKT: protein kinase B
AMPK: AMP-activated protein kinase
PP2B: protein phosphatase 2B
NAC: N-acetyl-L-cysteine
Vc: ascorbic acid
ROS: reactive oxygen species
LF: Long term fenvalerate culture
MAPK: Mitogen activated protein kinase
ERK: extracellular regulated kinase
DHE: dihydroethidium
DCFH: 2,7-dichlorodihydrofluorescein diacetate
CCK-8: cell counting kit-8
TMRM: Tetramethylrhodamine, methyl ester, perchlorate
GSK-3-β: Glycogen synthase kinase-3 beta
IR: insulin receptor
PTEN: Phosphatase and tensin homolog deleted on chromosome 10
JNK: c-Jun N-terminal kinase
IRS1: Insulin Receptor Substrate 1
FBS: fetal bovine serum
DMEM: Dulbecco’s Modified Eagle’s medium
2-NBDG: 2-(N-(7-nitrobenz-2-oxa-1,3-diazol-4-yl)amino)-2-deoxyglucose
LDH: lactate dehydrogenase
MMP: mitochondrial membrane potential (MMP)
RTKs: receptor tyrosine kinases
HCQ: hydroxychloroquine
mTOR: mammalian target of rapamycin
PP1: protein phosphatase one
PP2A: protein phosphatase 2A
TCA: tricarboxylic acid
PTPs: protein tyrosine phosphatases
NFAT: nuclear factor of activated T cells
HCC: hepatocellular carcinoma
