# Supplementary material for: Fenvalerate exposure induces AKT/AMPK-dependent alterations in glucose metabolism in hepatoma cells
Source: Front Pharmacol. 2025 Feb 25;16:1540567. doi: 10.3389/fphar.2025.1540567 (PMC11893604; doi:10.3389/fphar.2025.1540567)

Fig.2A

p-IR

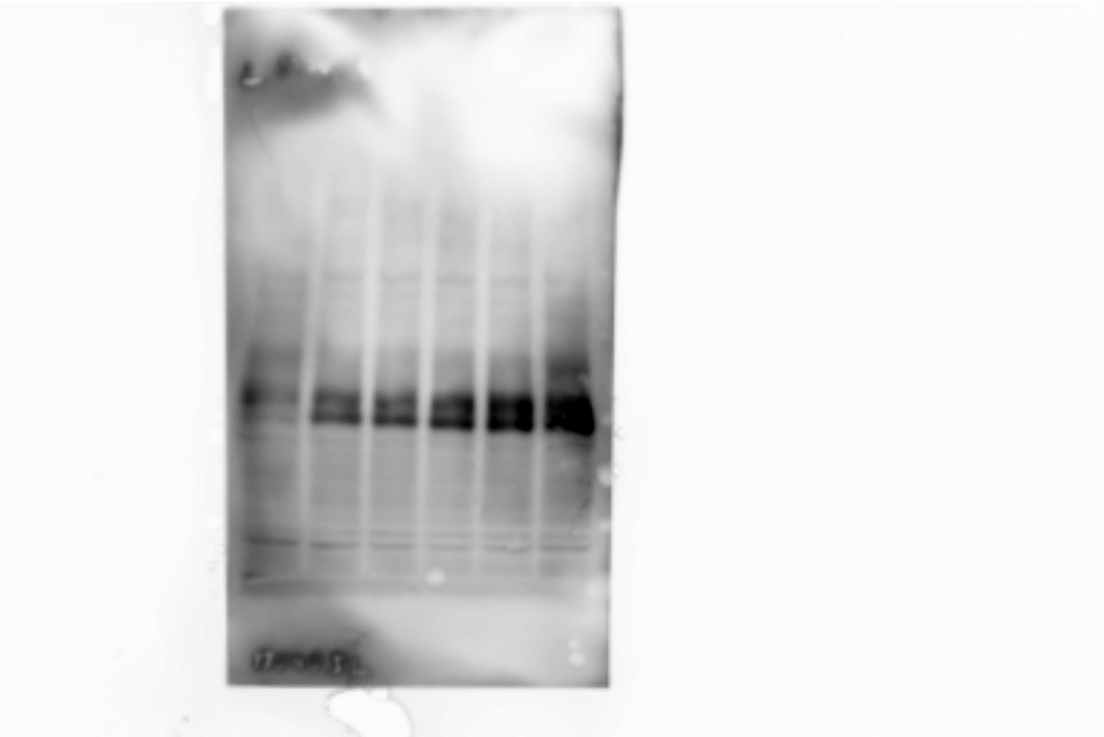

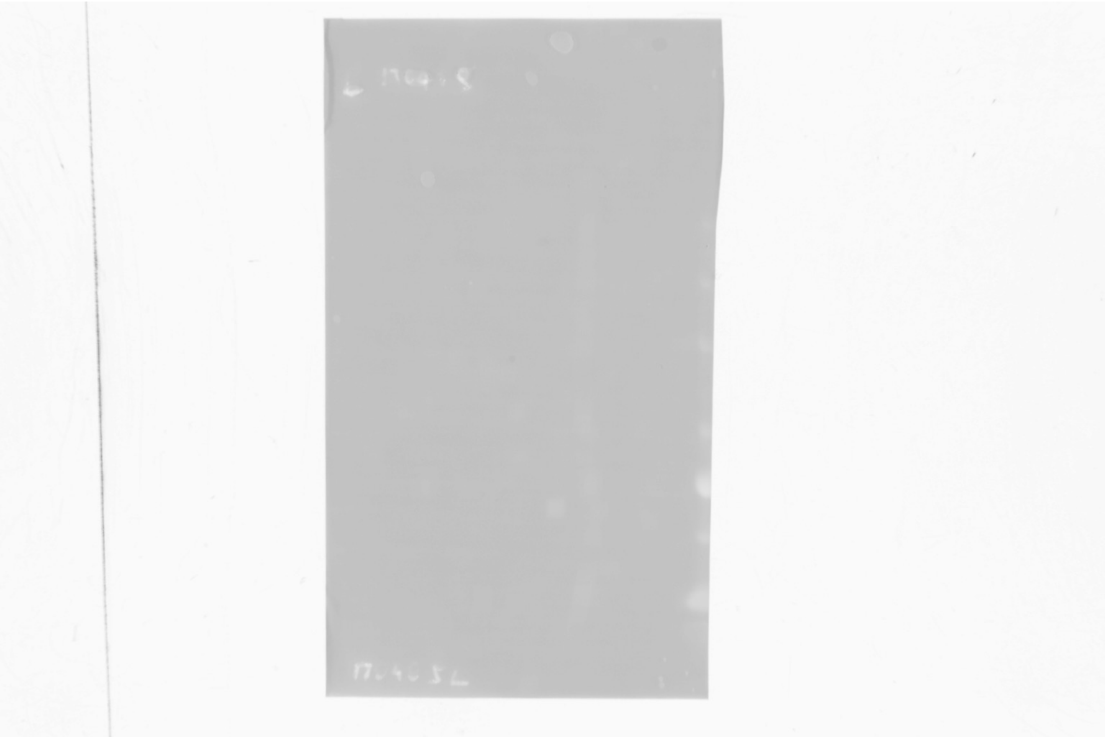

IR-beta

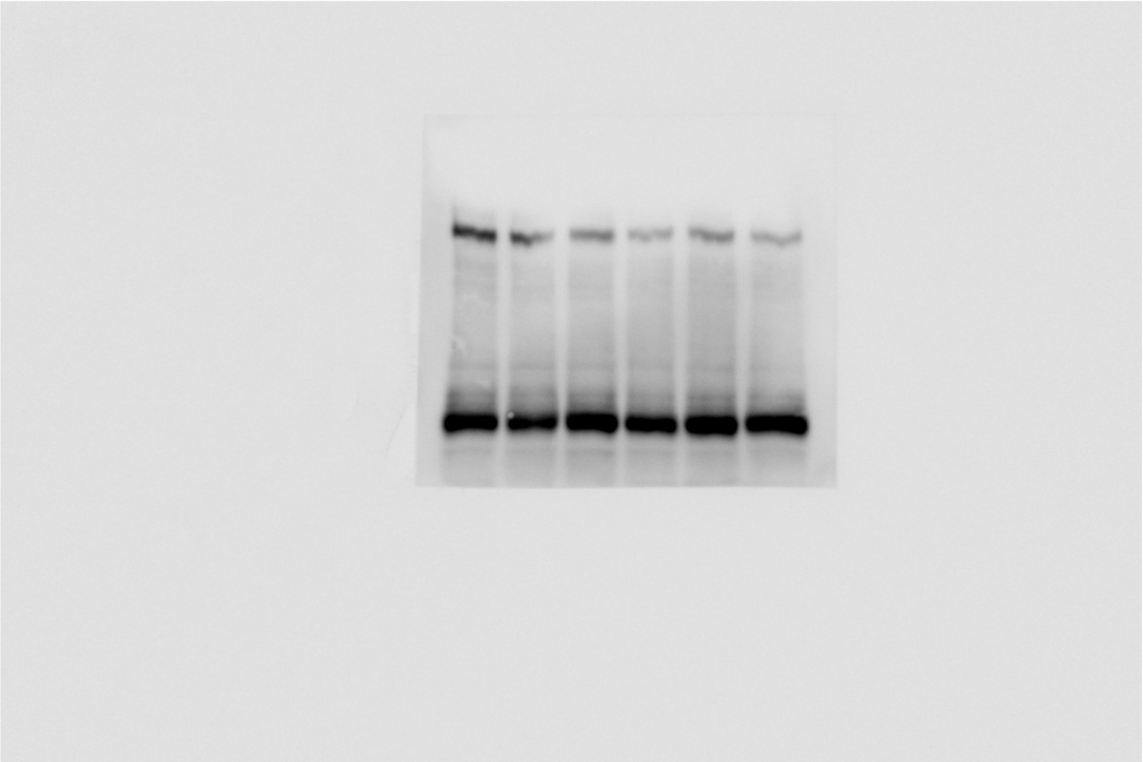

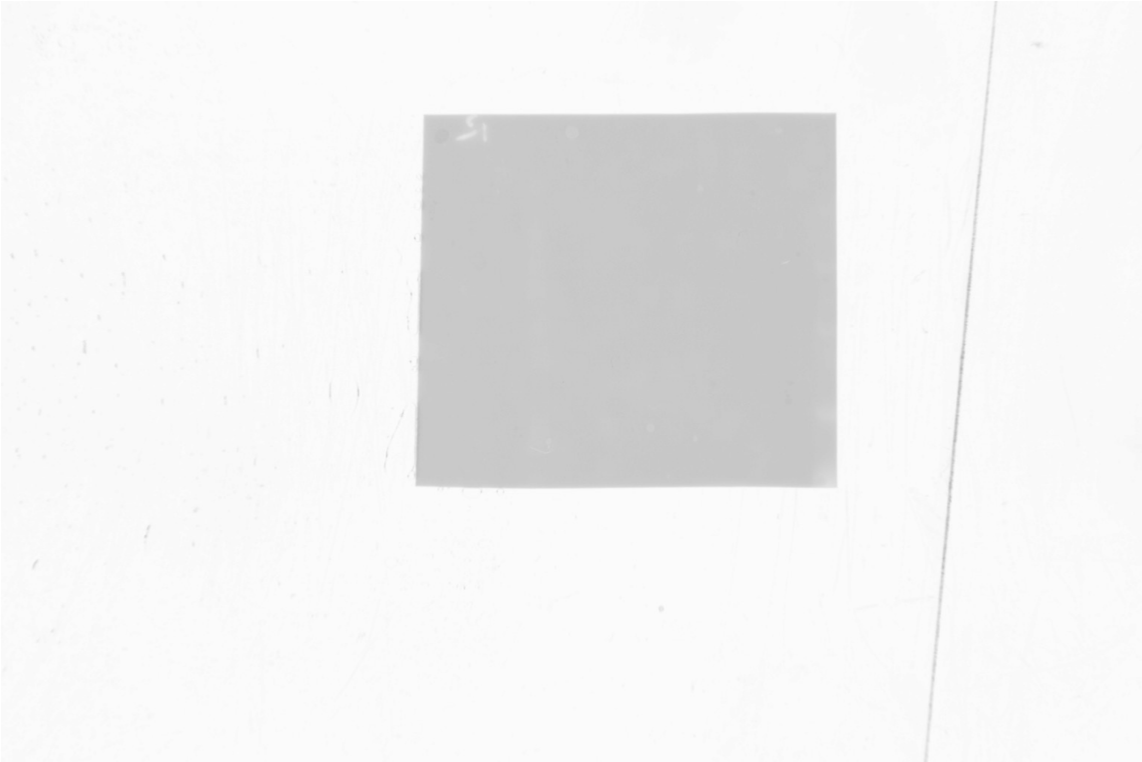

p-AKT473

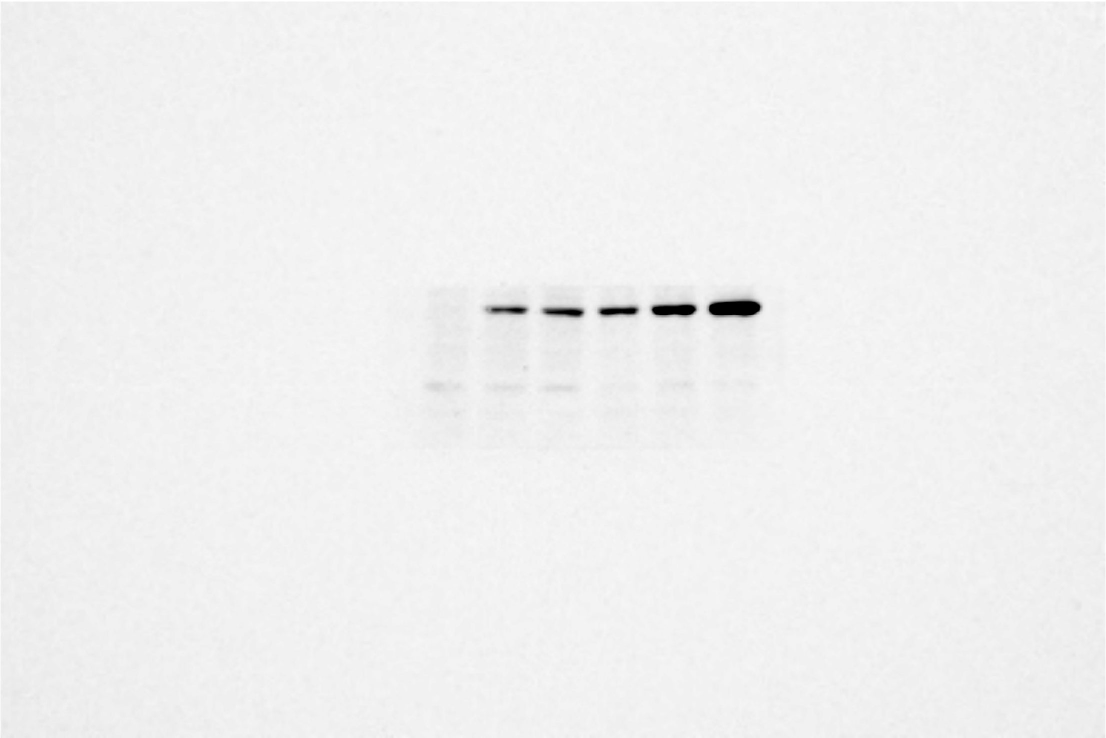

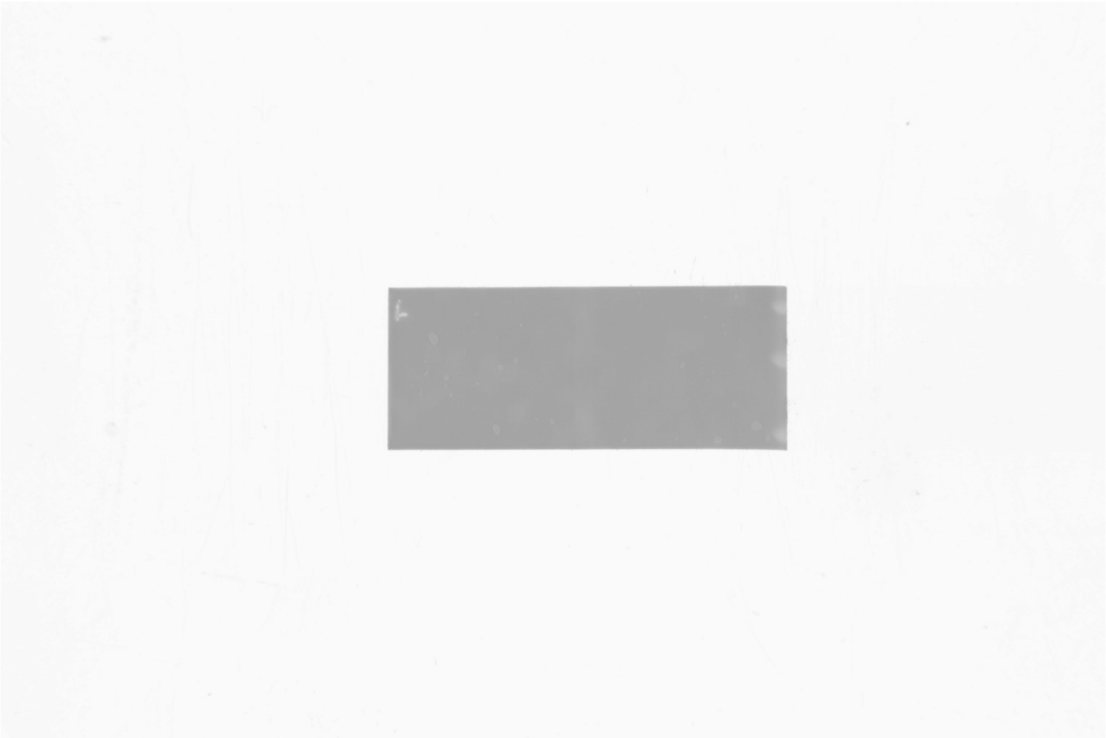

AKT

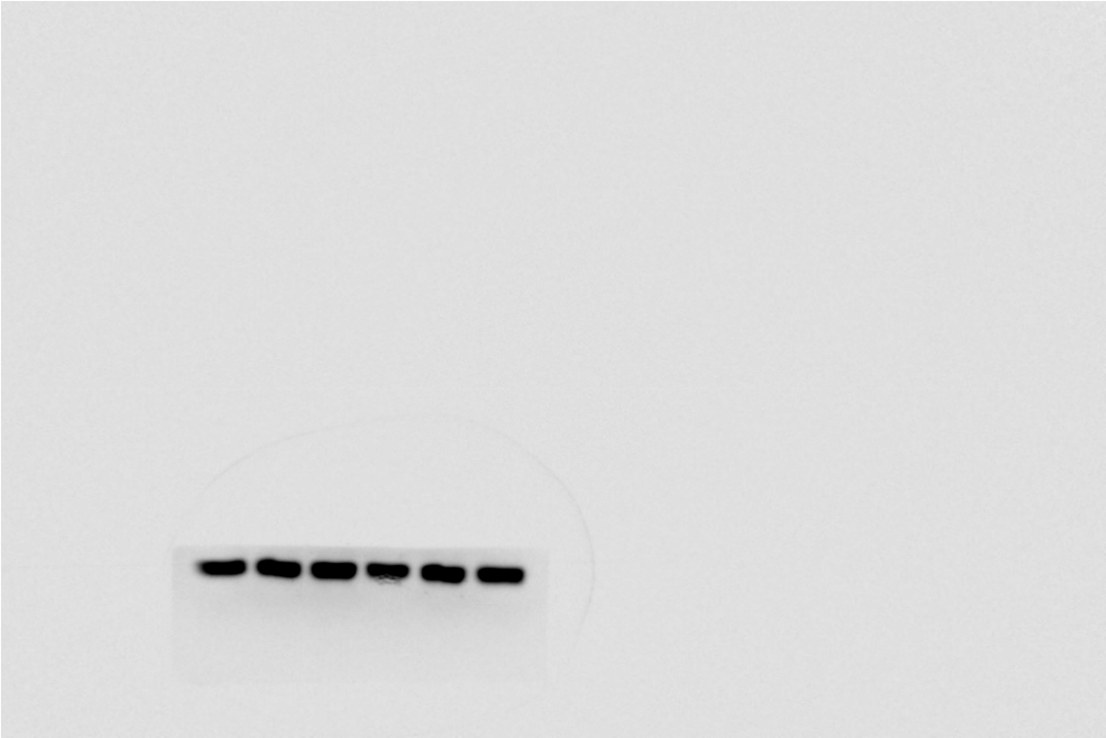

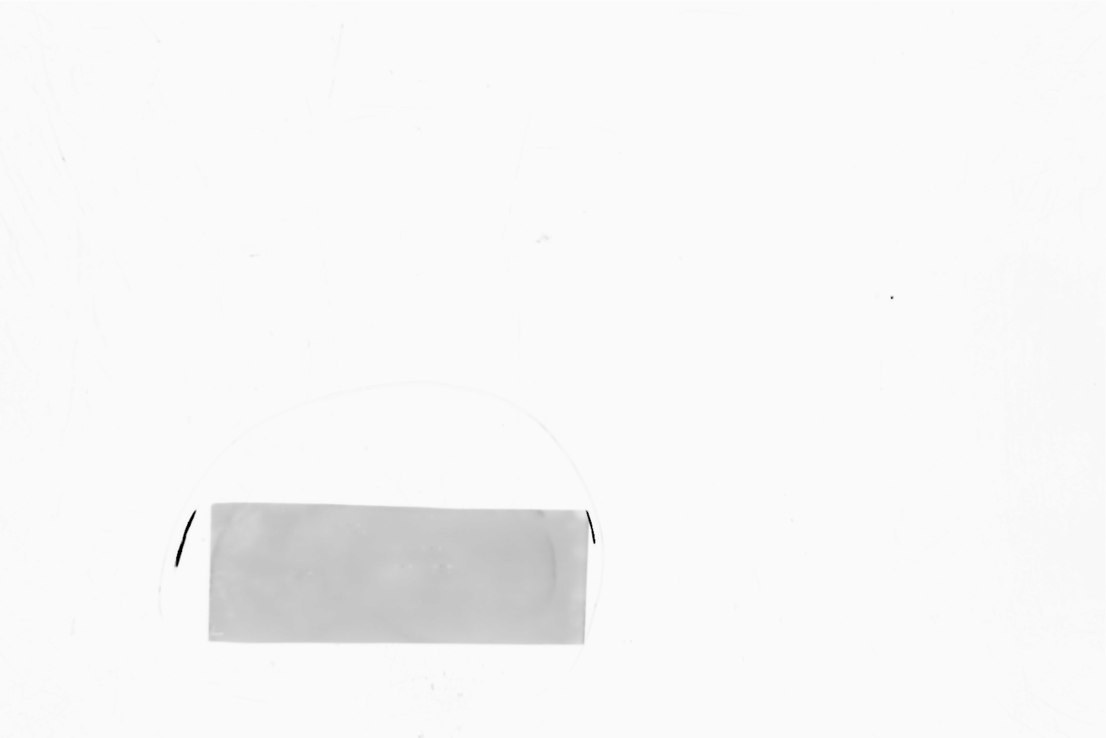

p-GSK-3beta

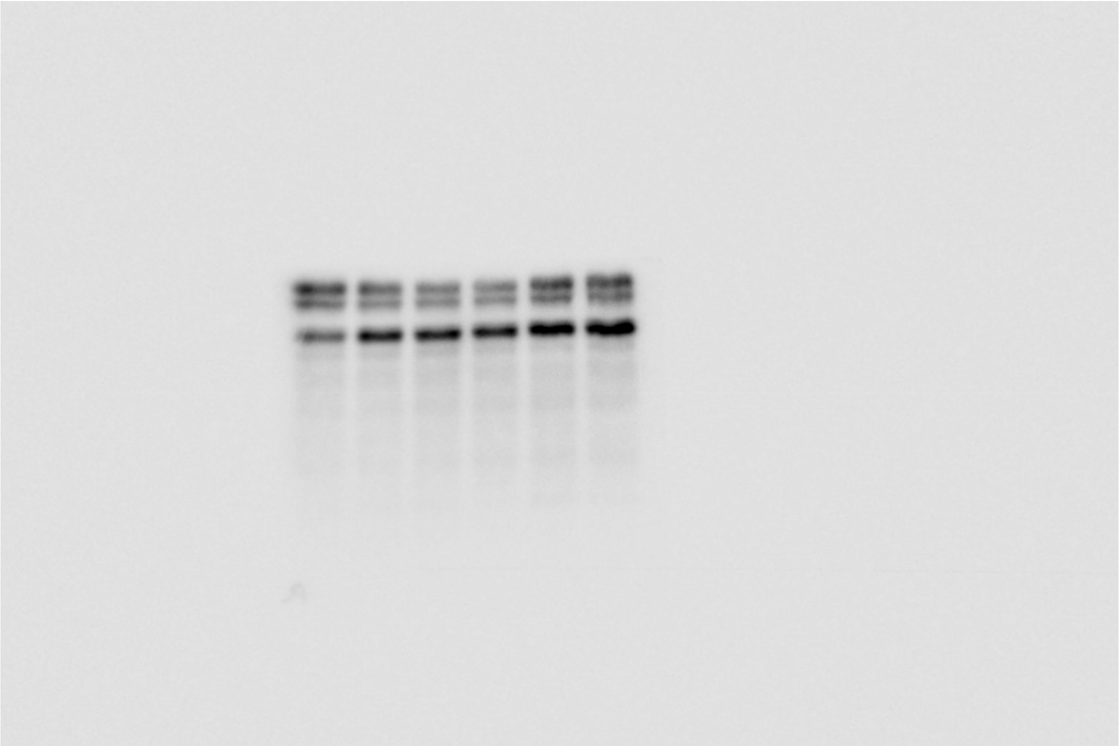

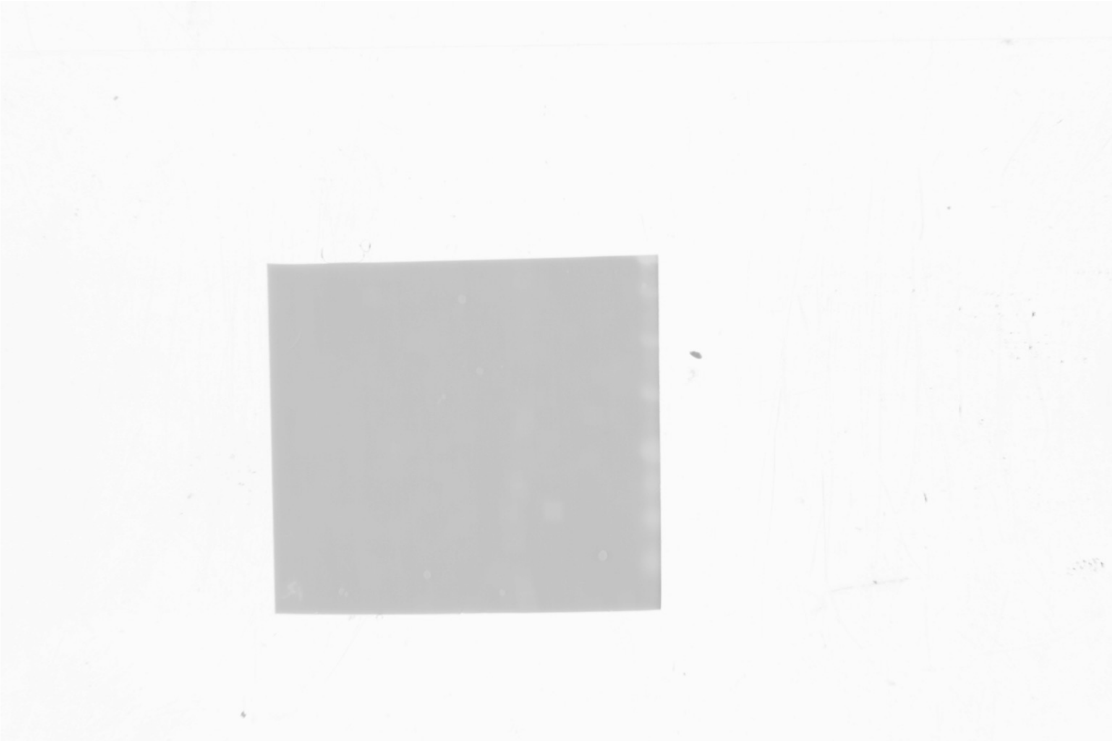

GSK-3beta

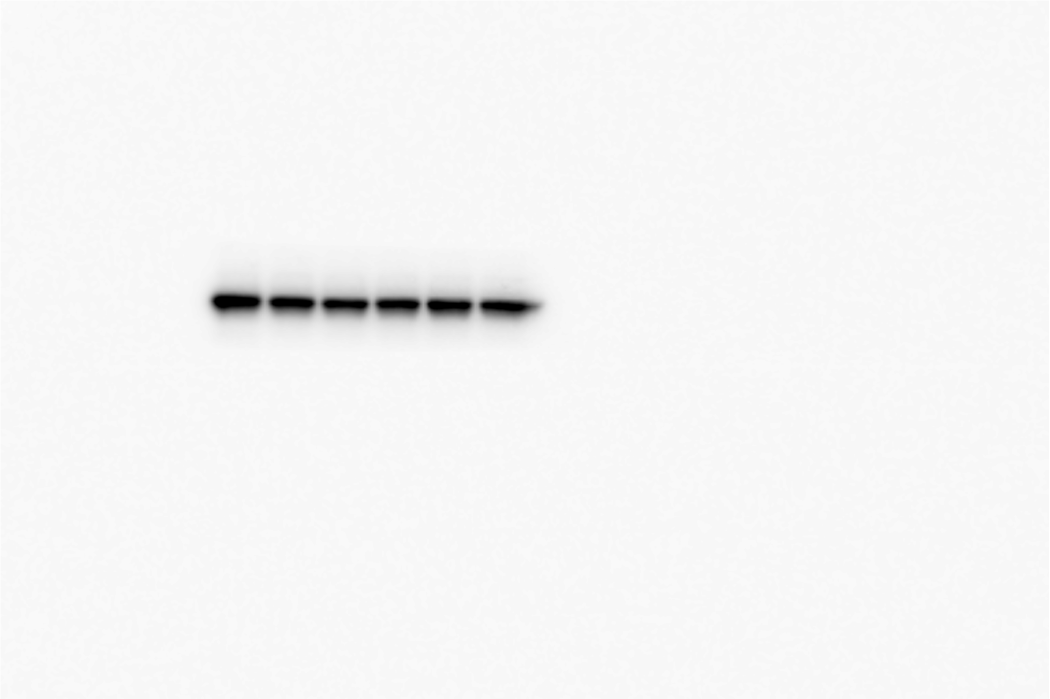

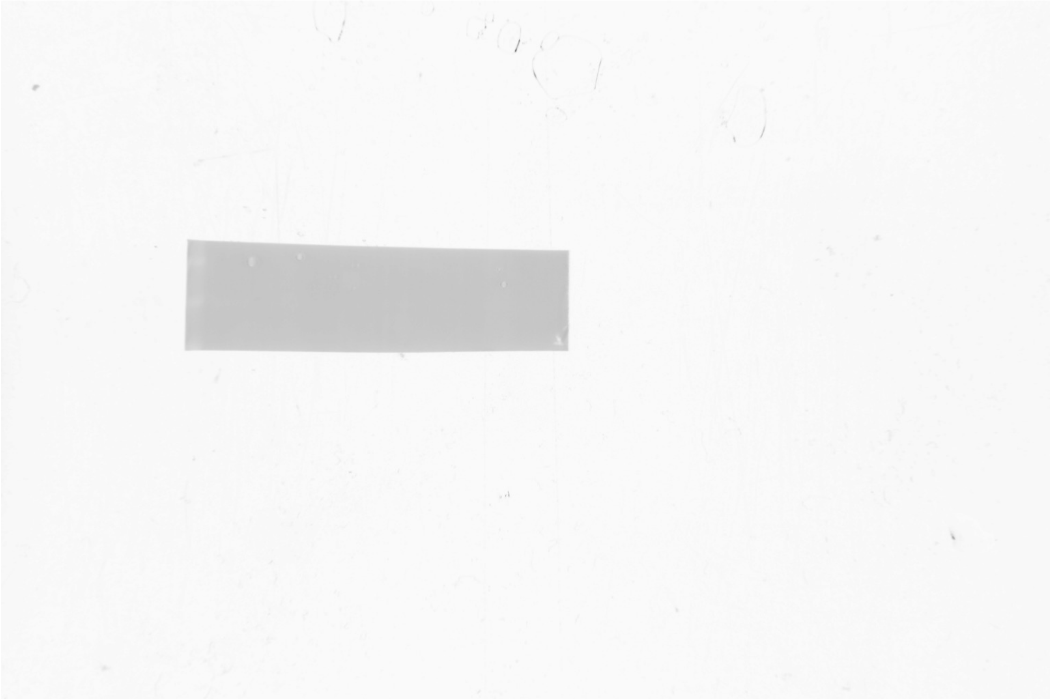

p-AMPK

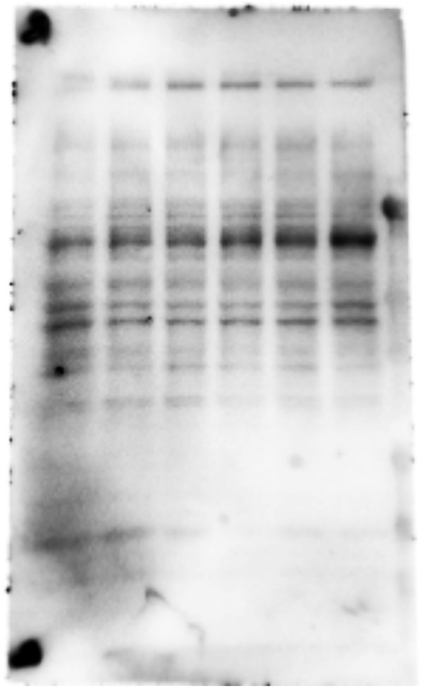

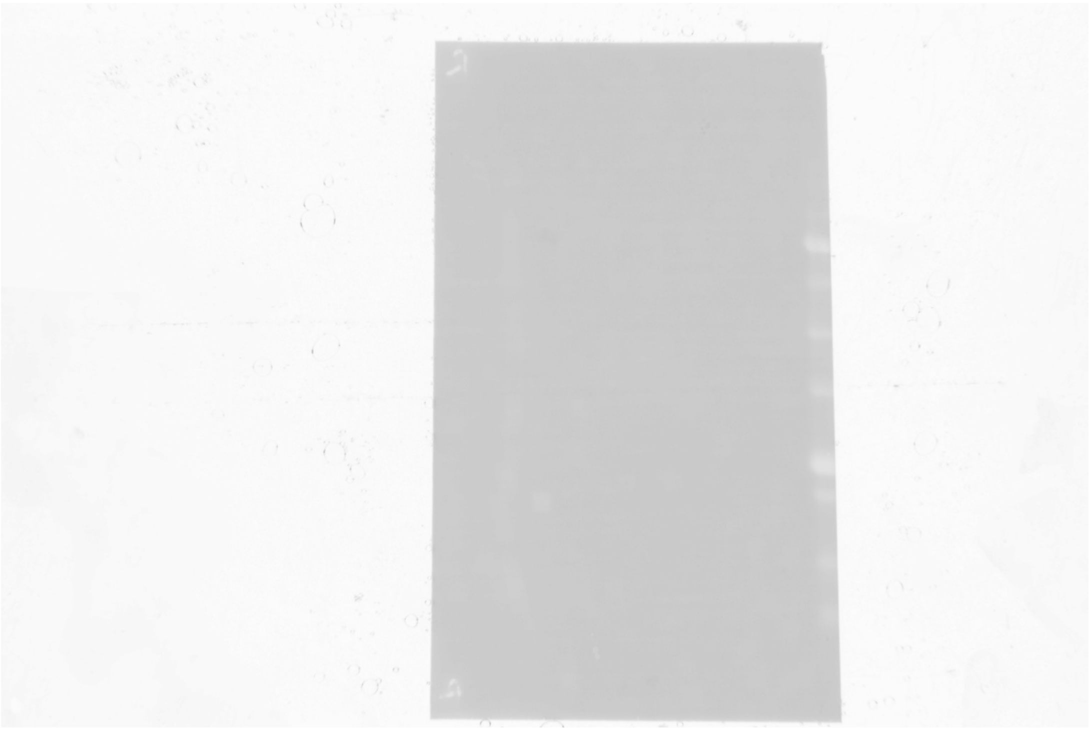

p-AMPK

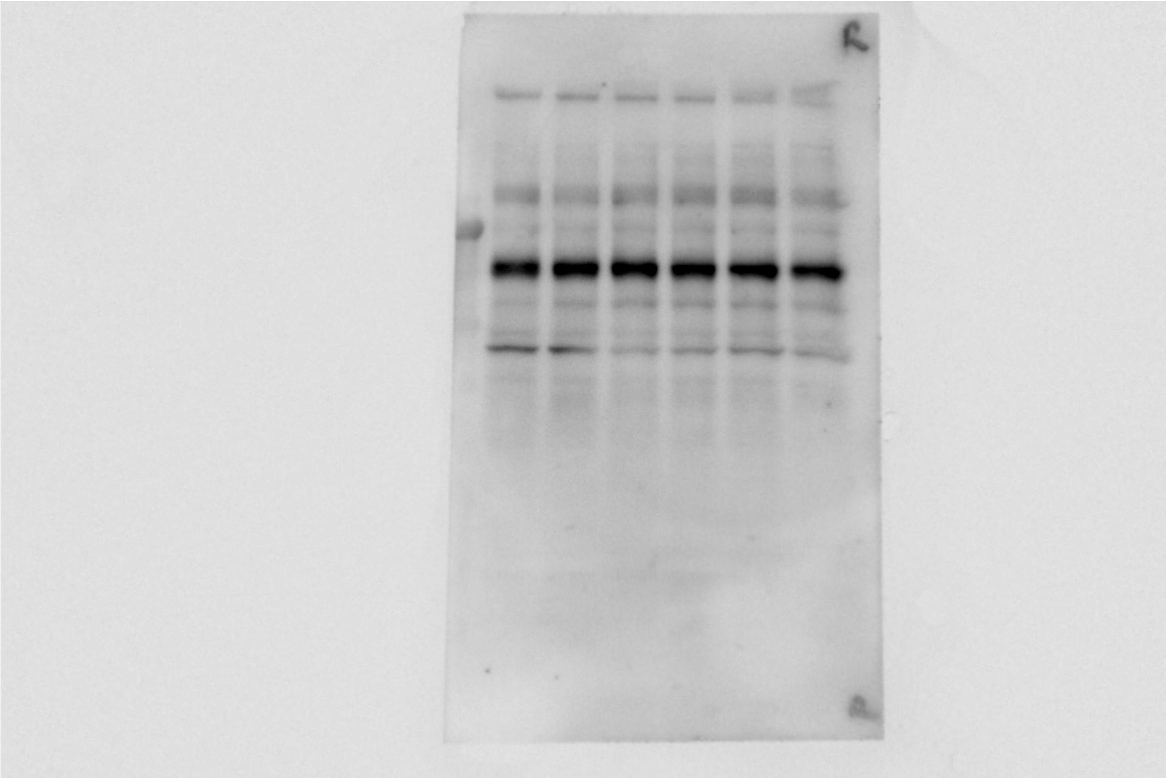

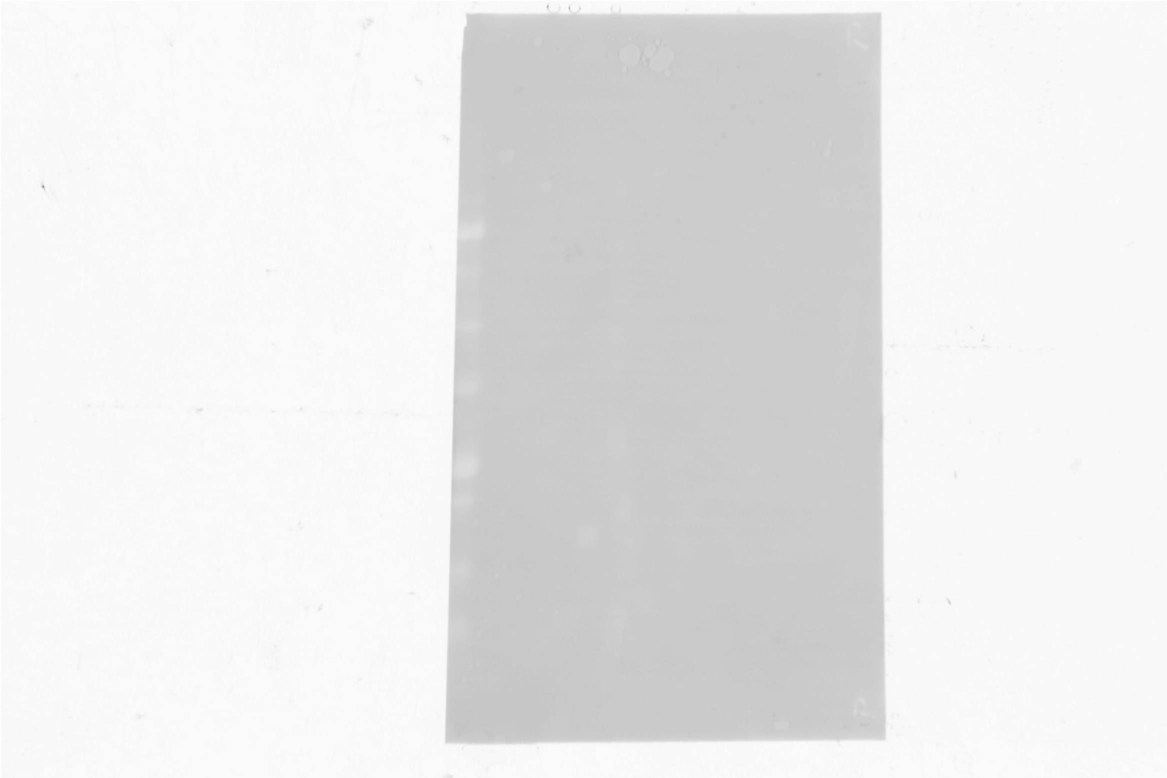

beta-actin

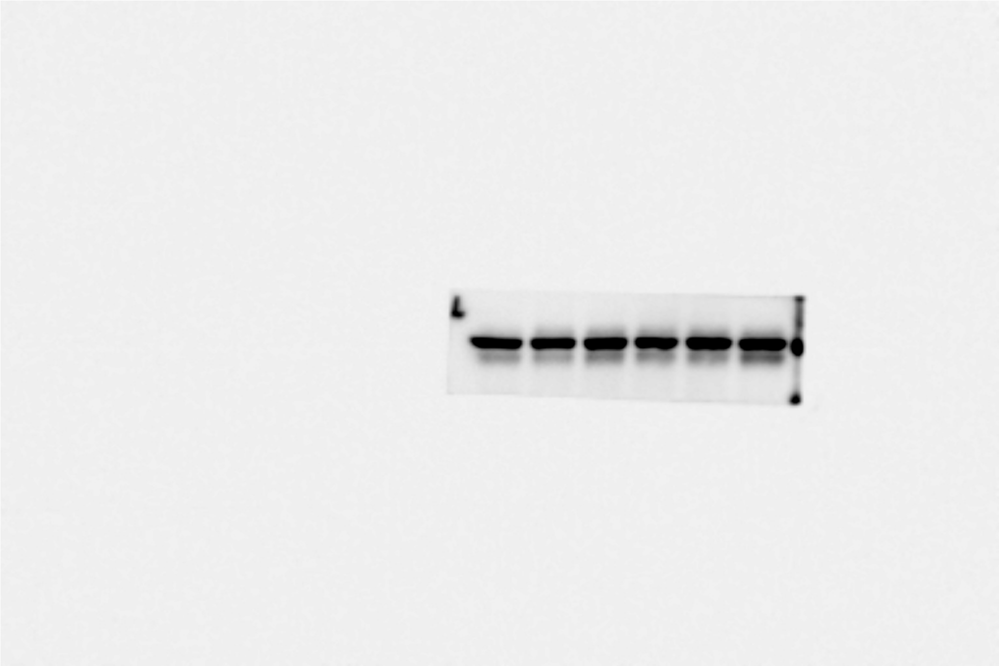

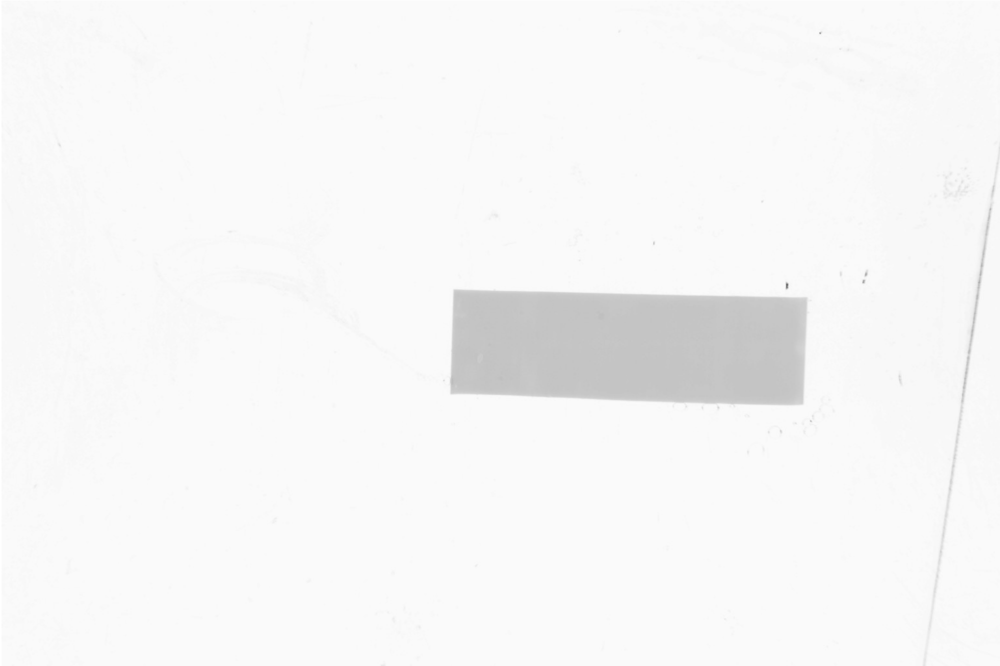

Fig.2B

p-ERK

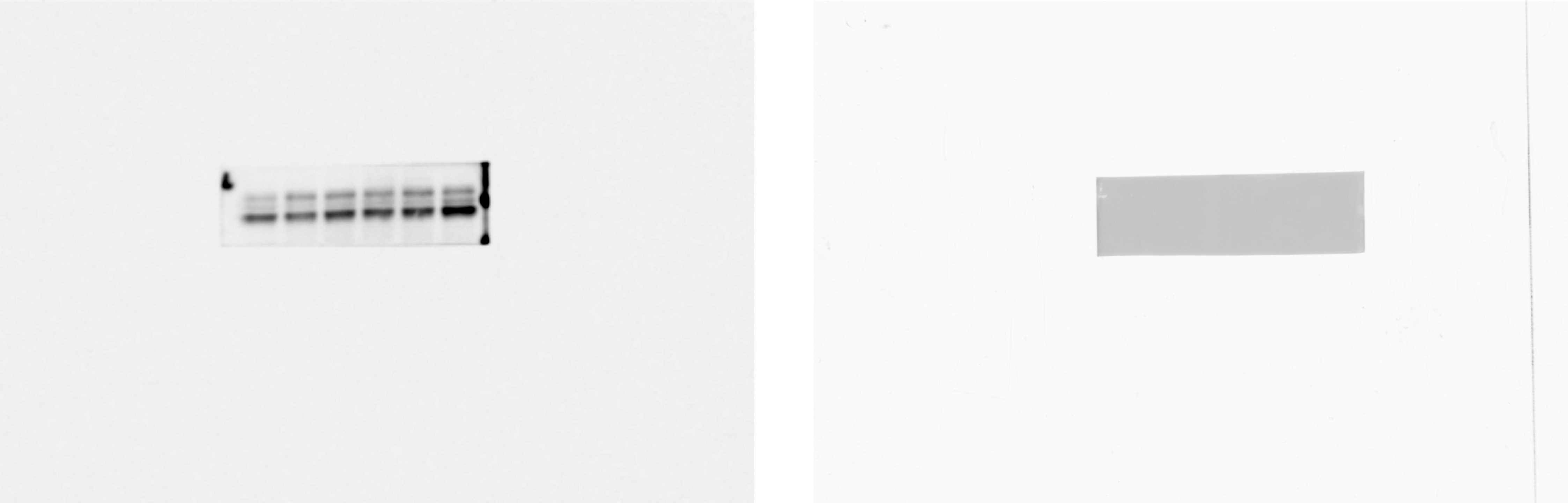

ERK

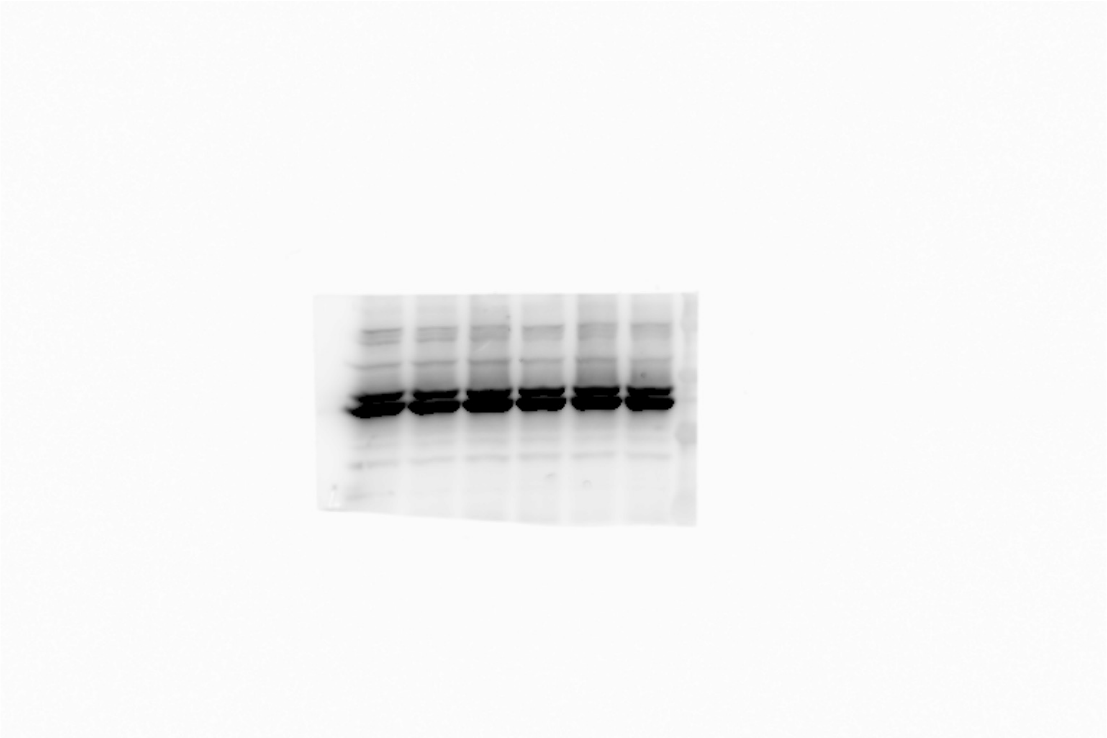

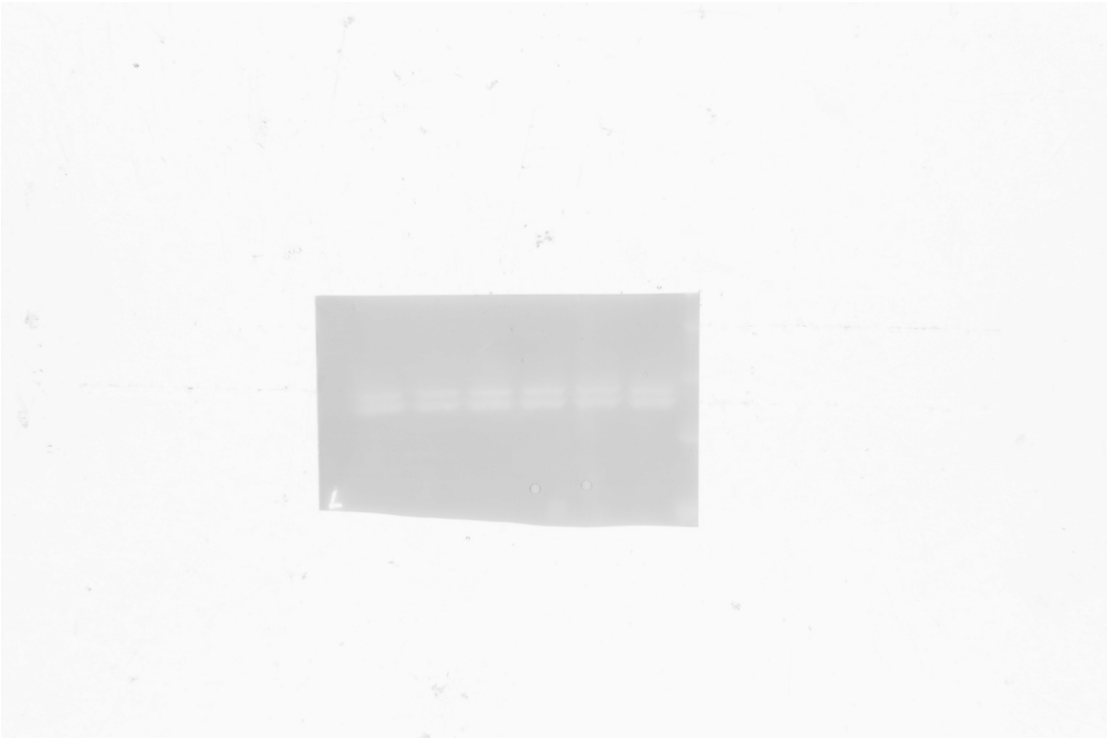

p-JNK

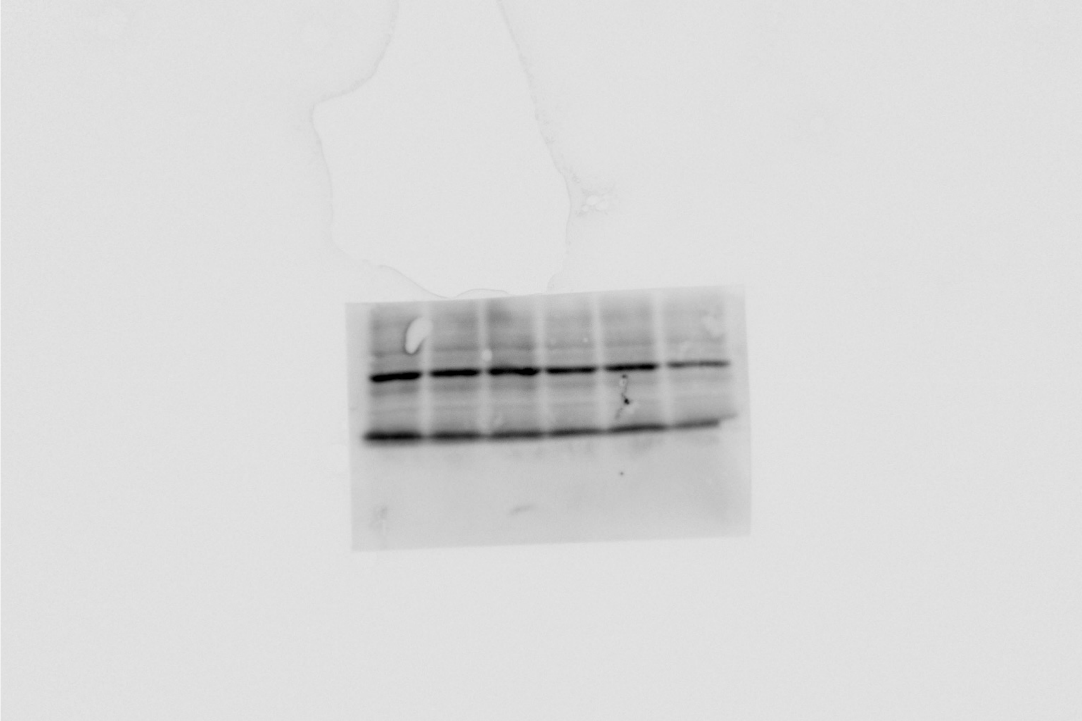

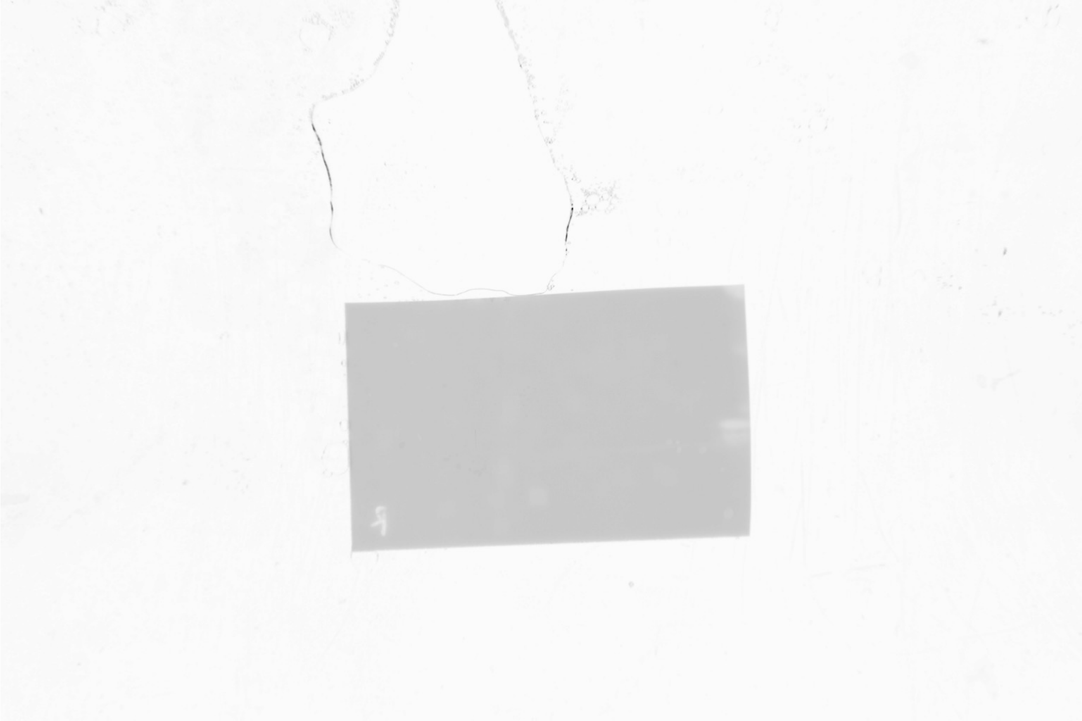

beta-actin

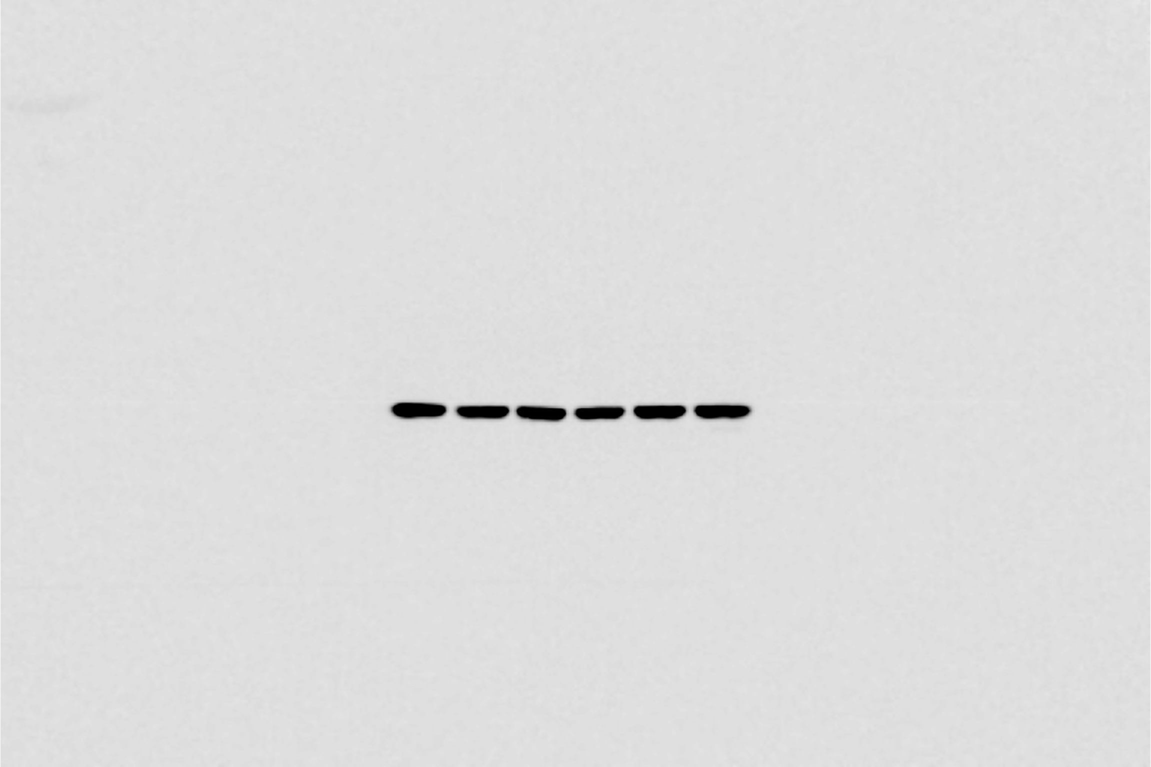

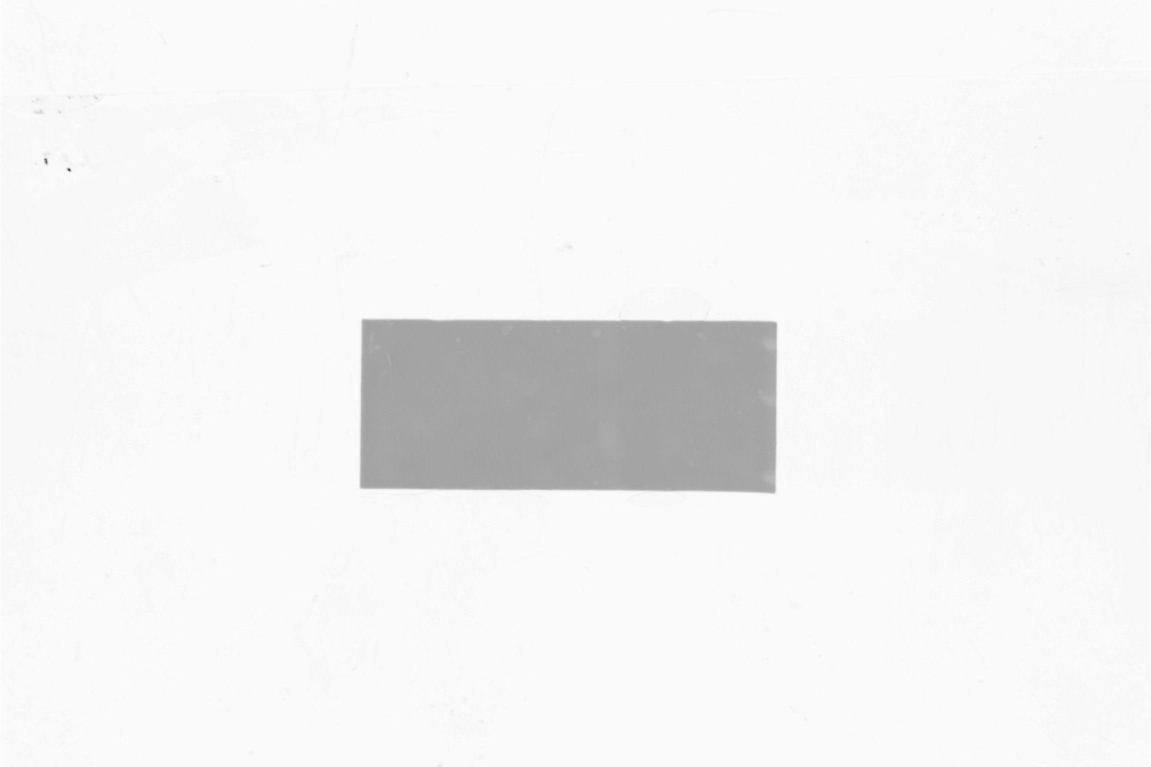

Fig.2C

p-IR

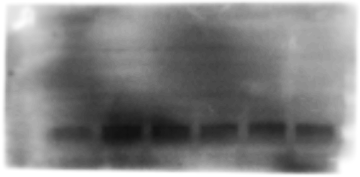

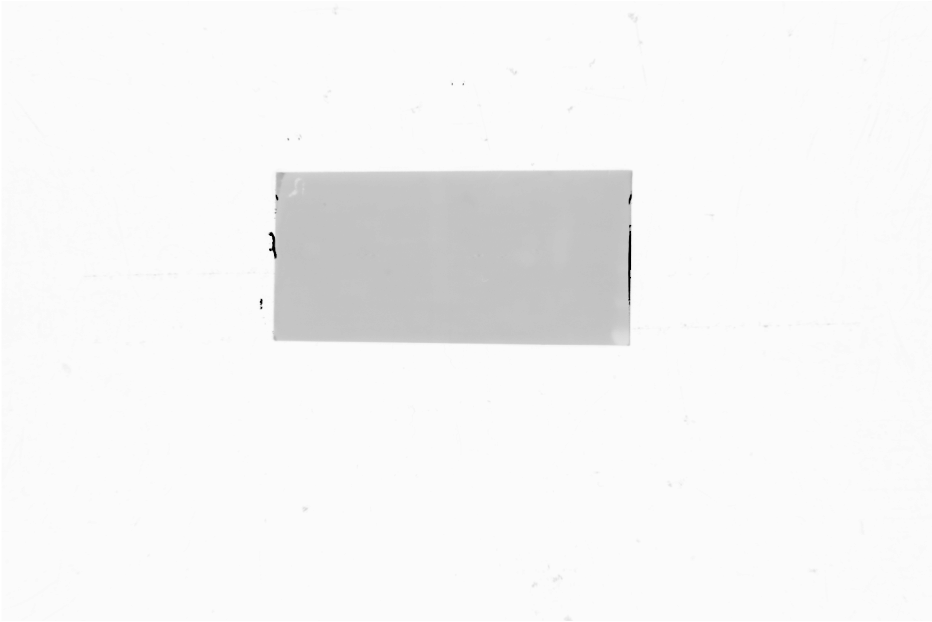

p-PTEN

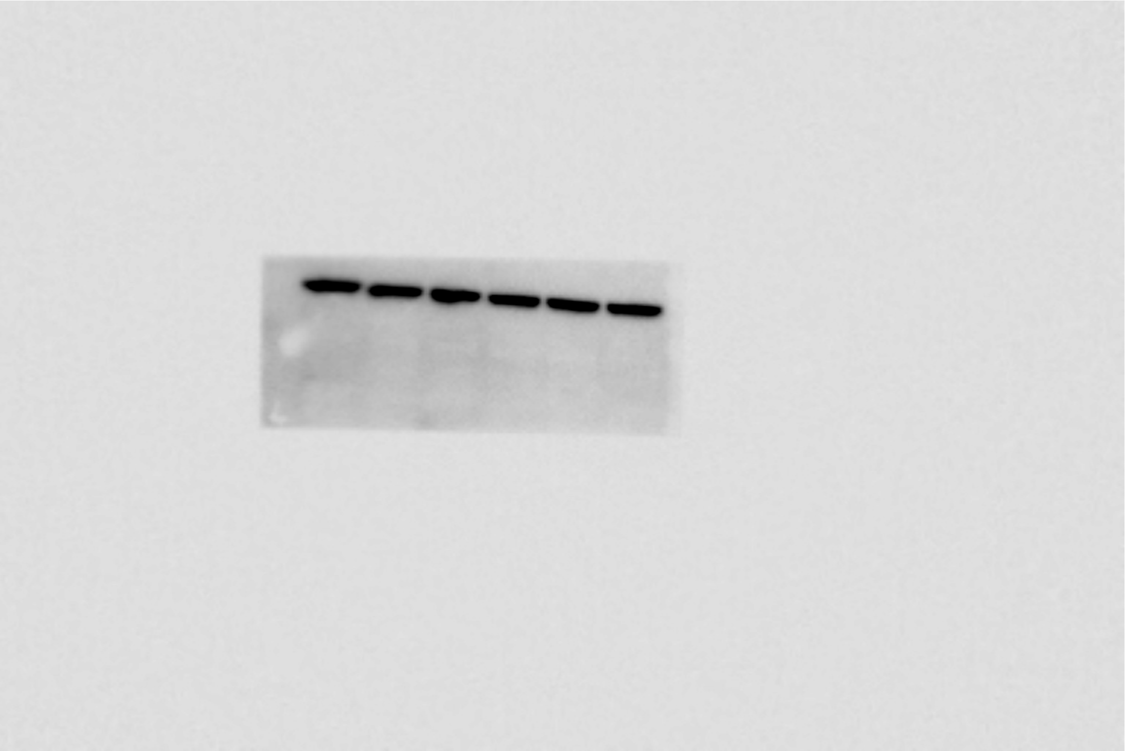

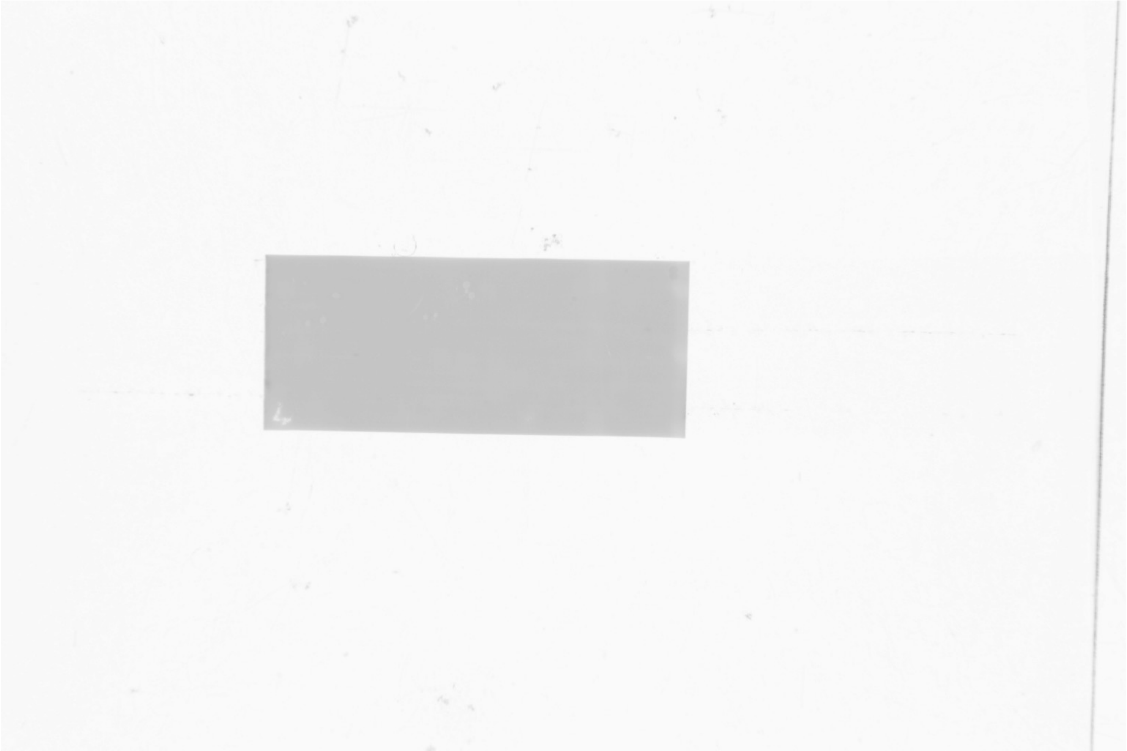

PTEN

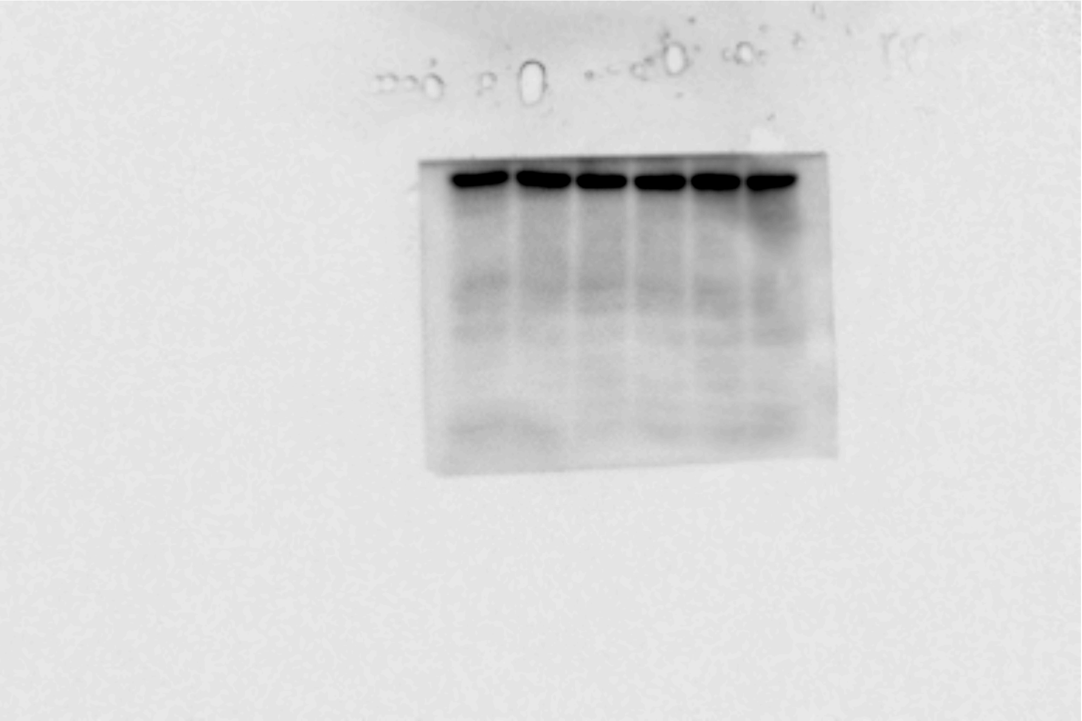

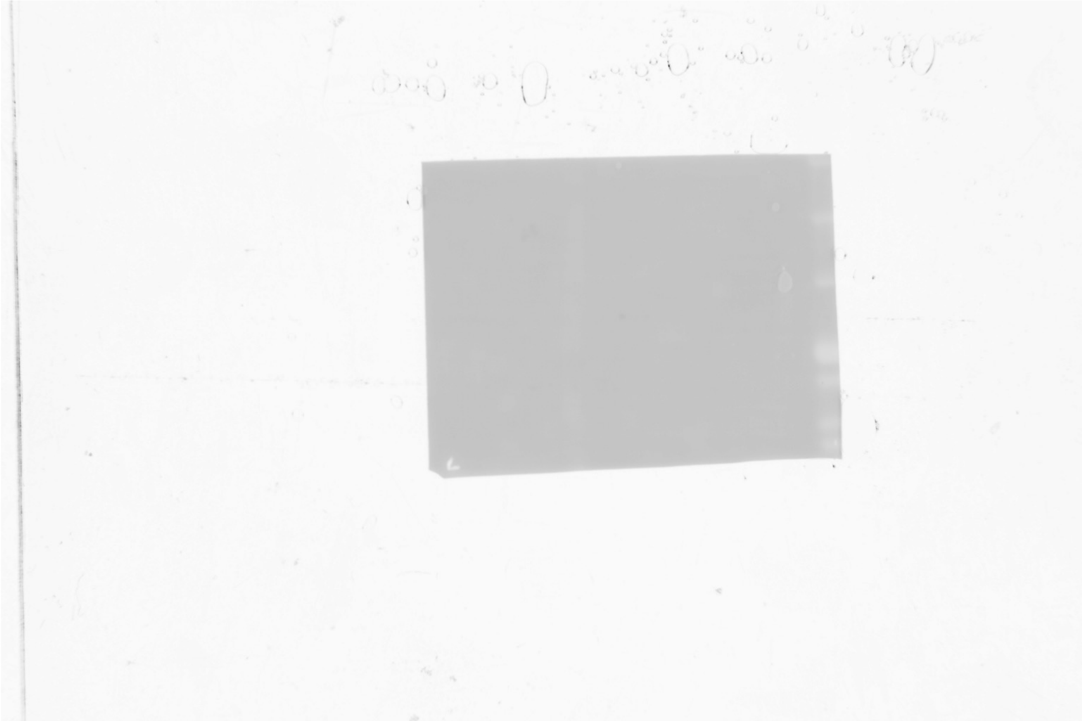

p-AKT473

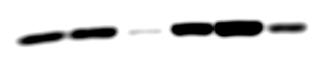

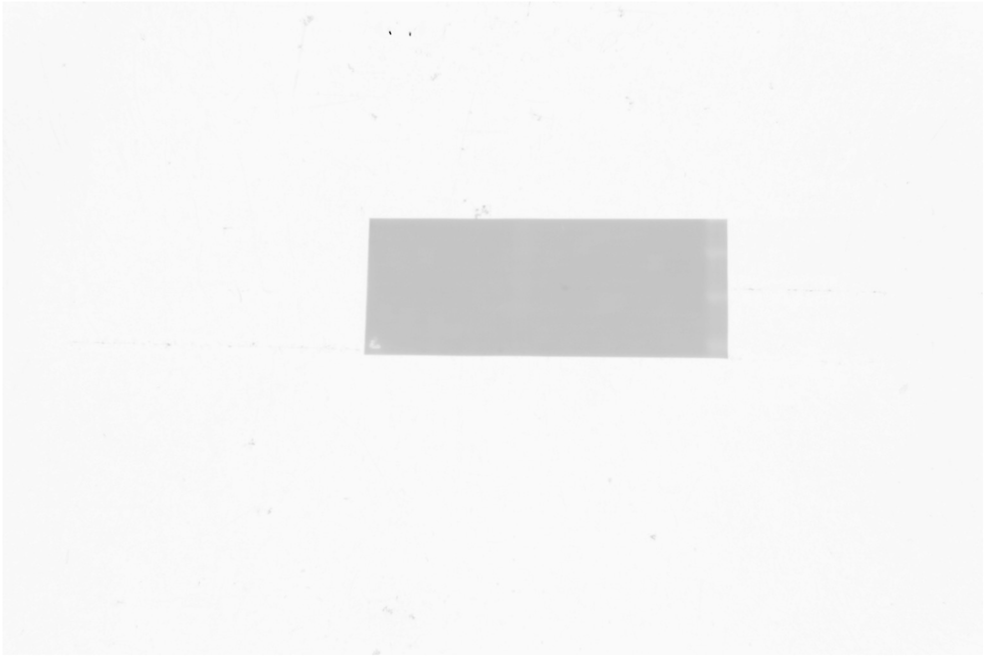

p-ERK

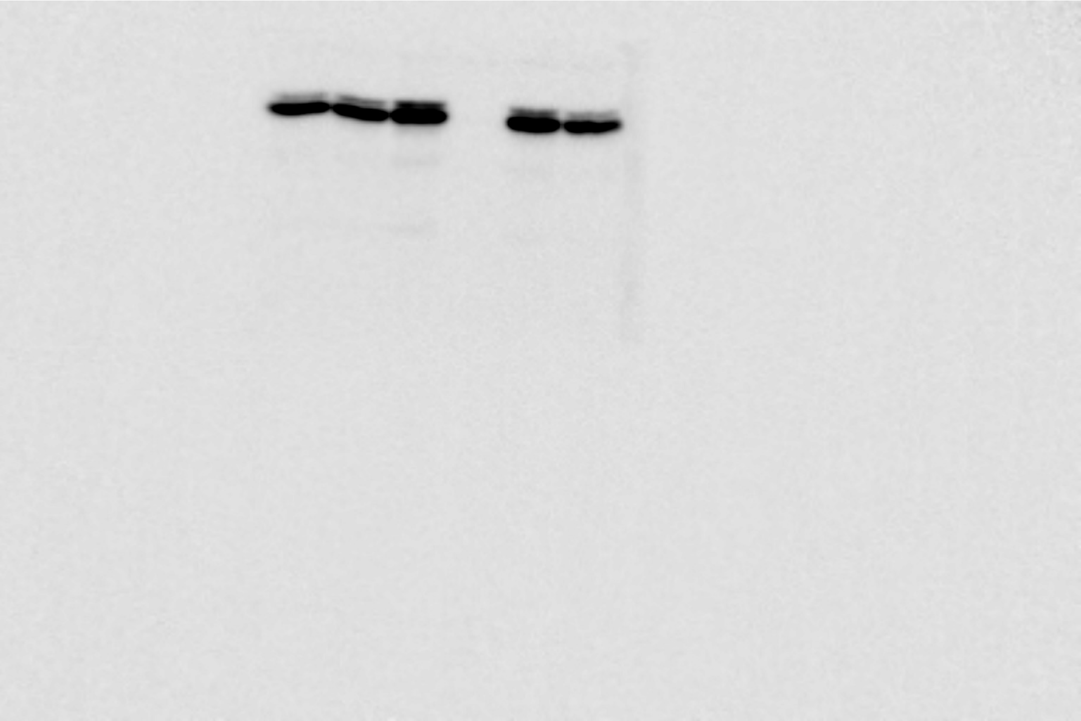

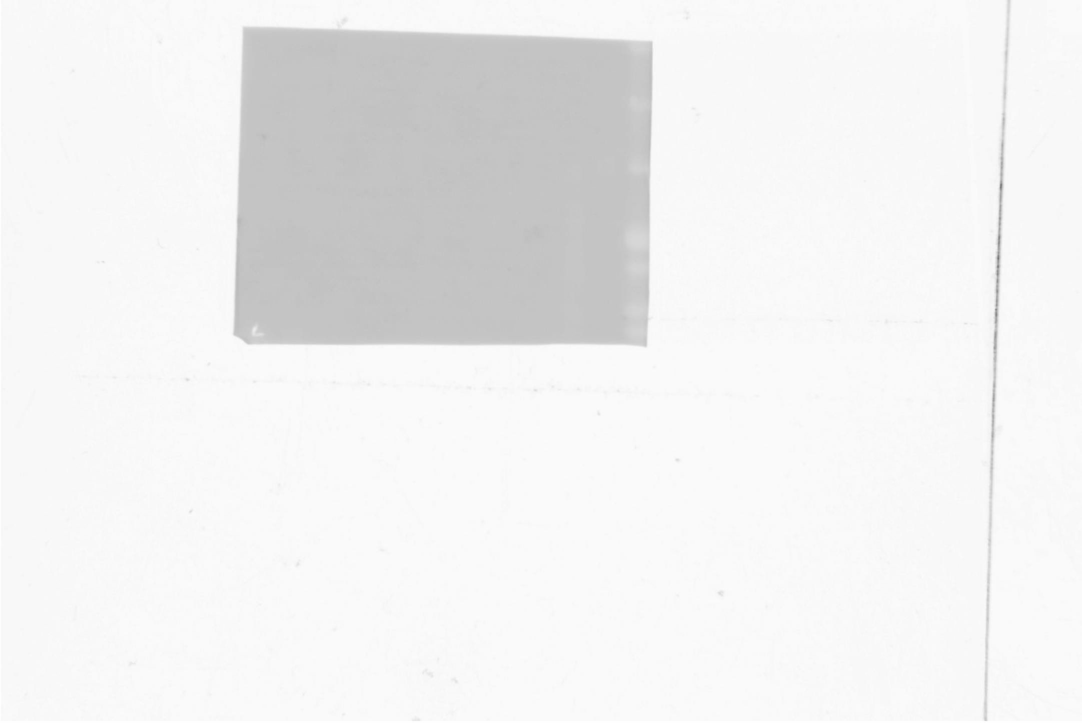

p-JNK

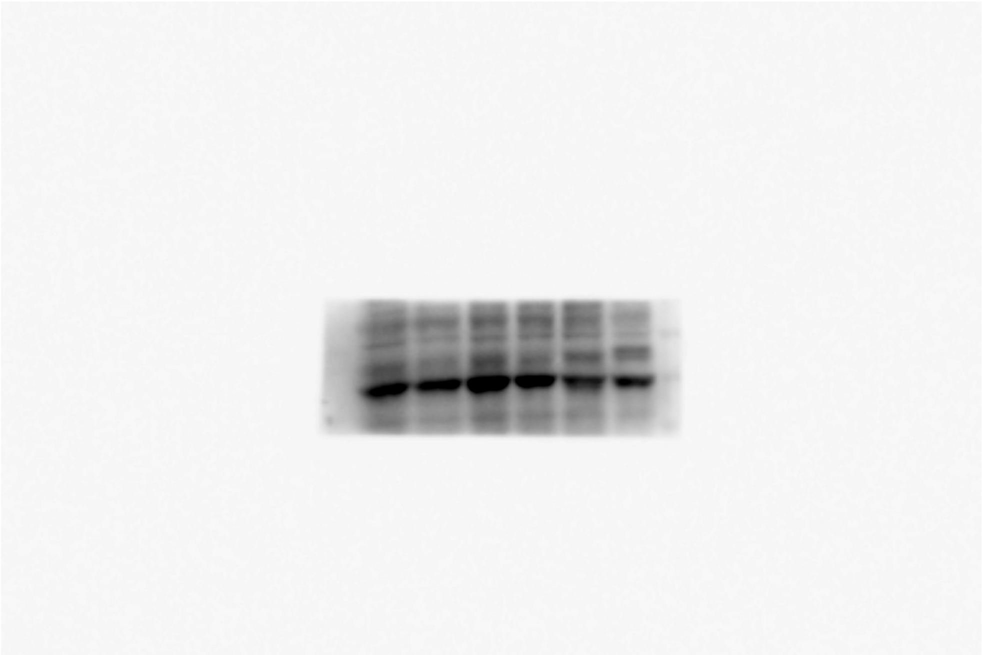

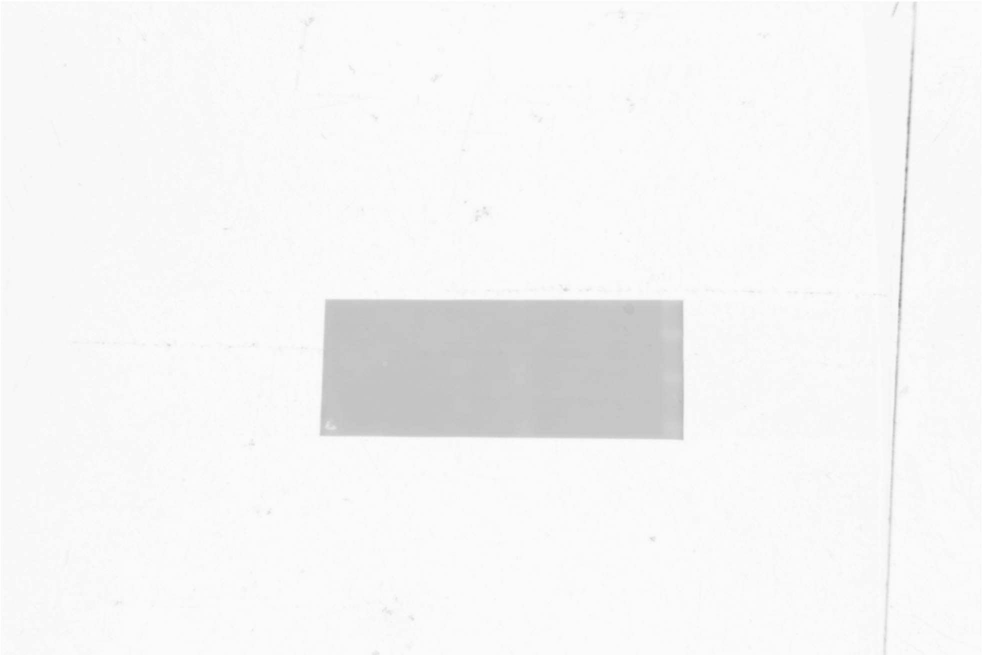

beta-actin

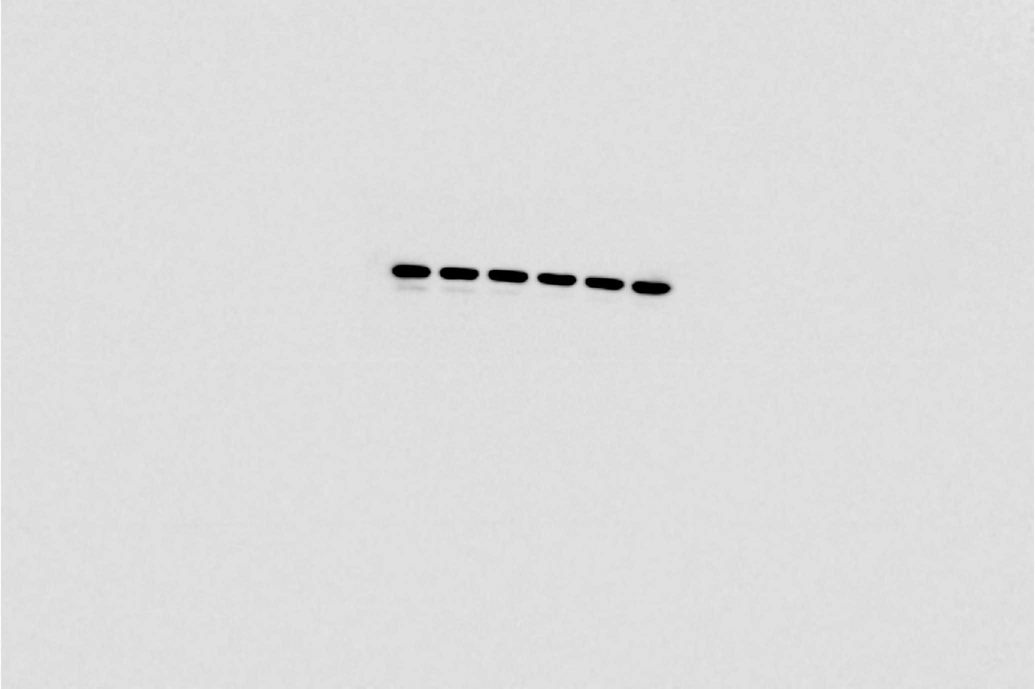

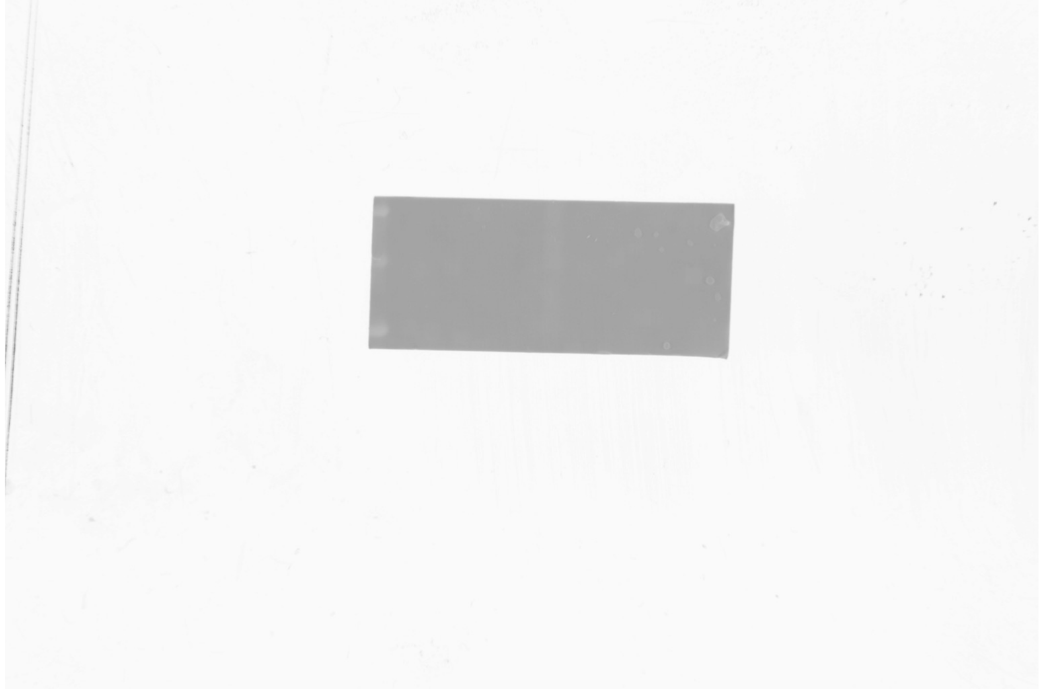

Fig.3A

IRS1

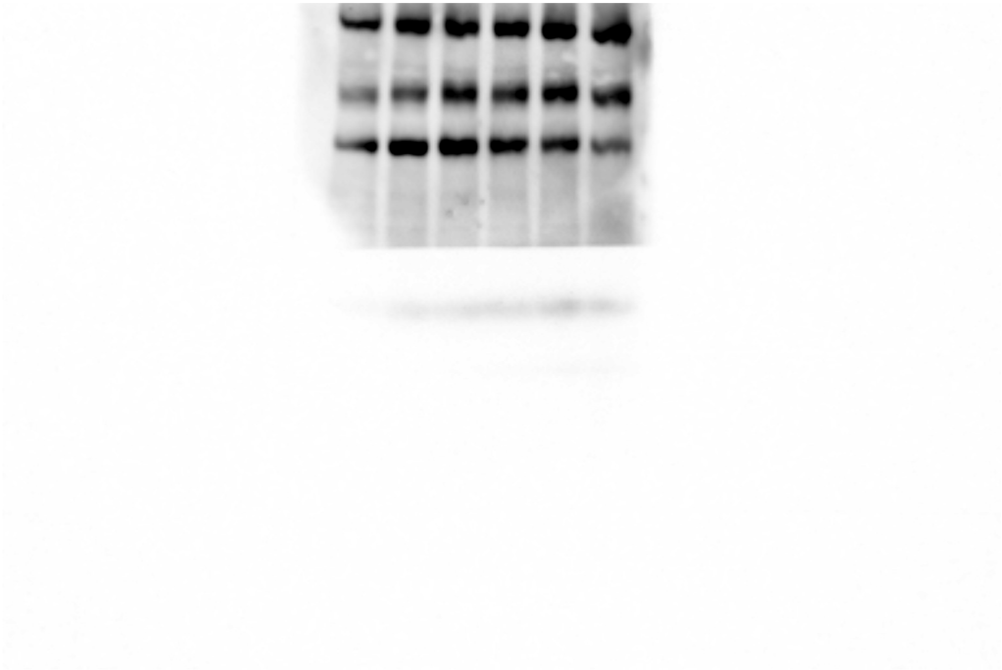

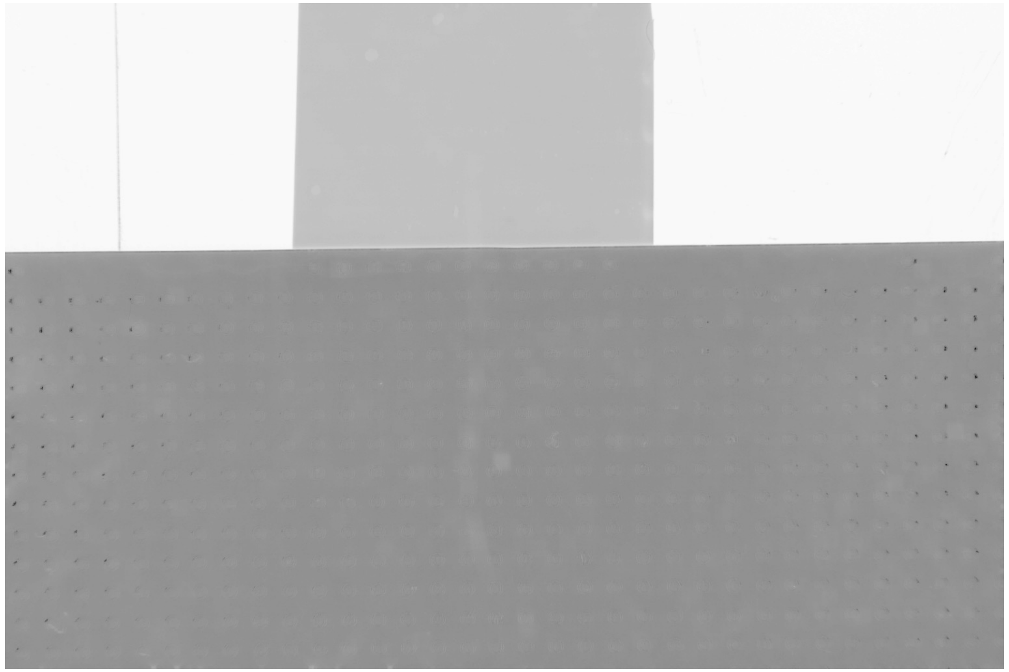

p-mTOR

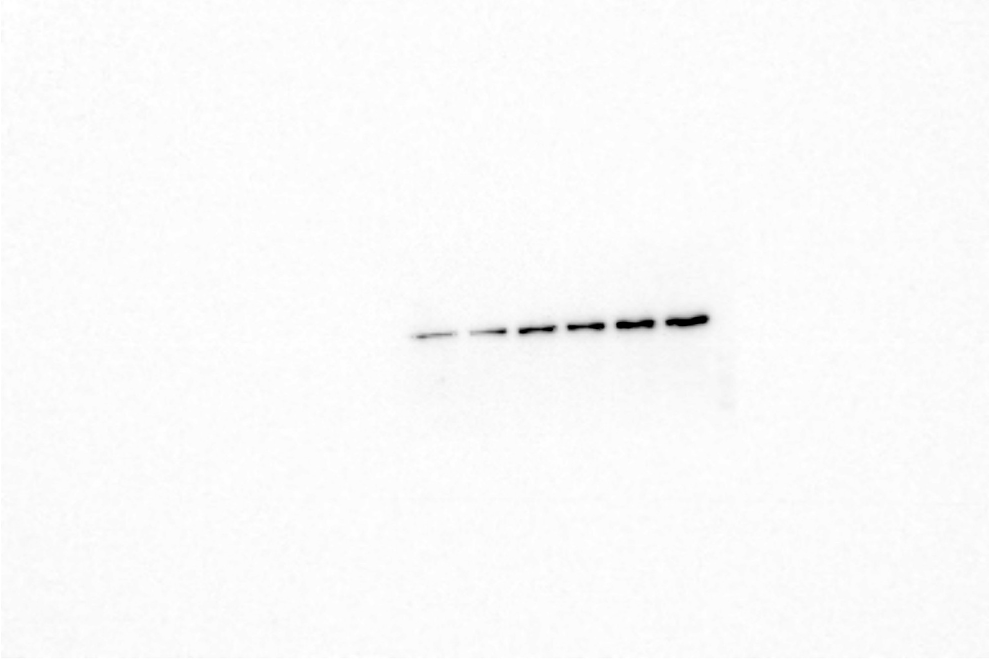

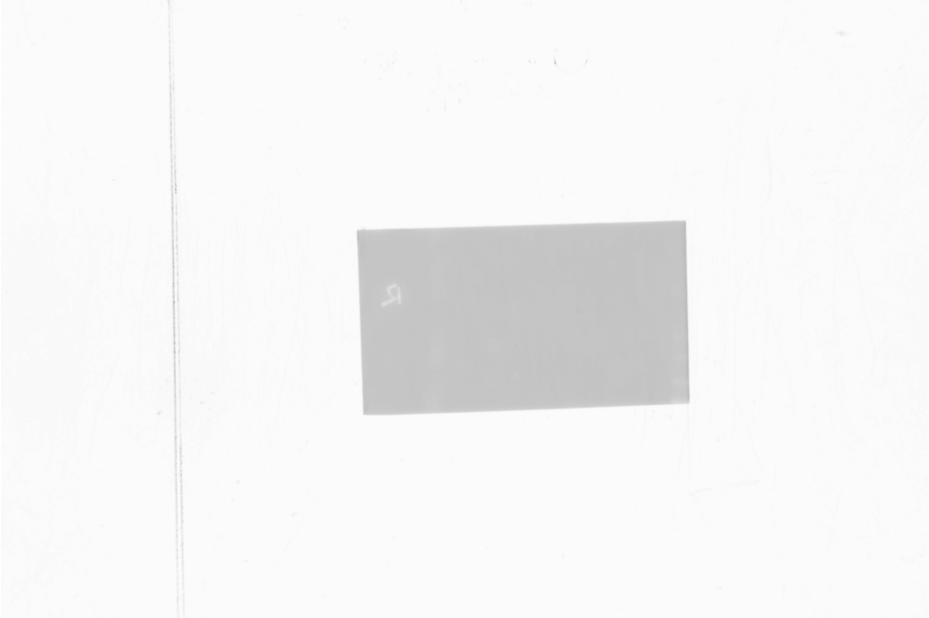

beta-actin

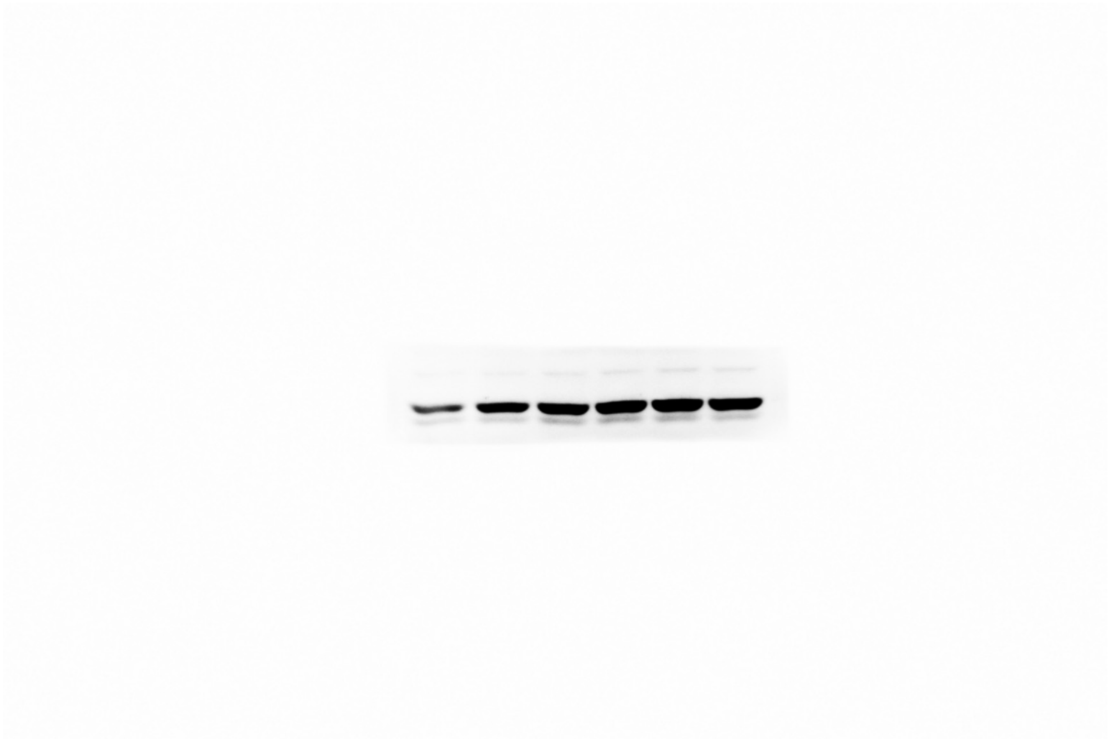

Fig.3D

IRS1

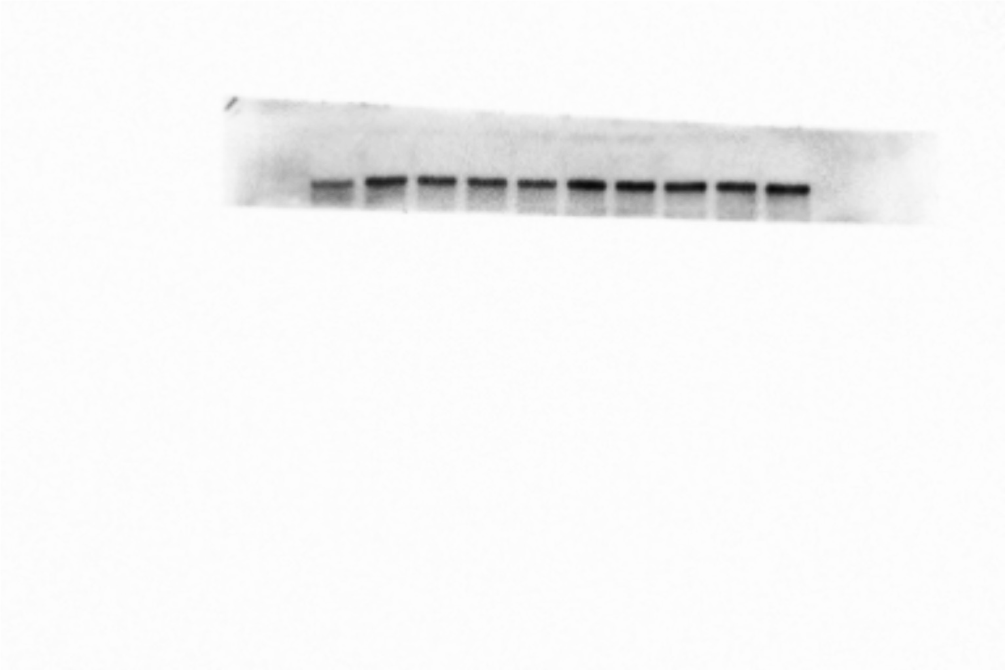

p-IR

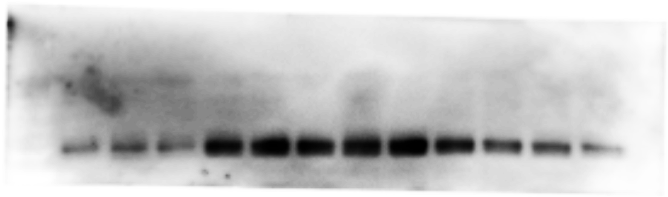

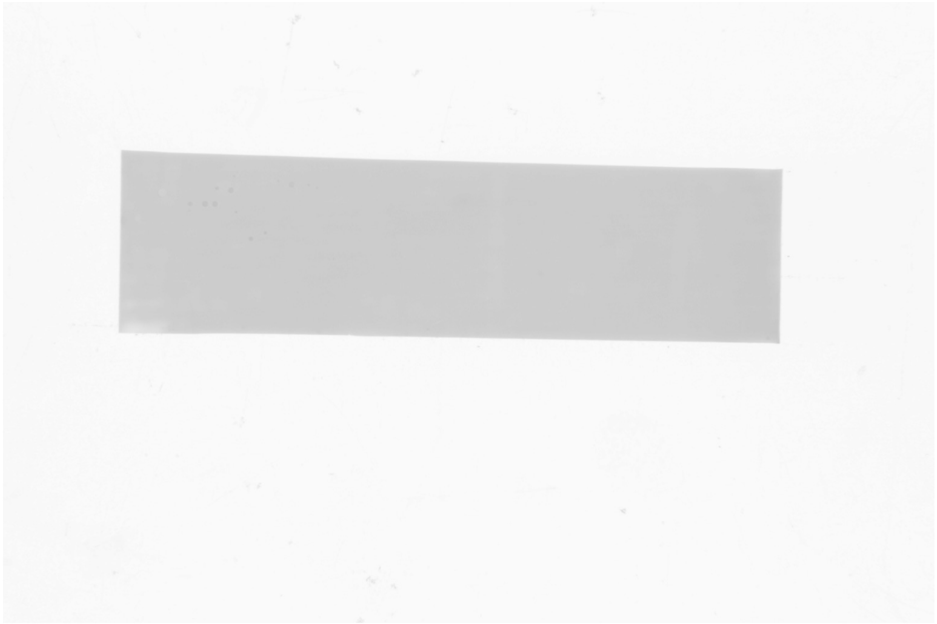

p-AMPK

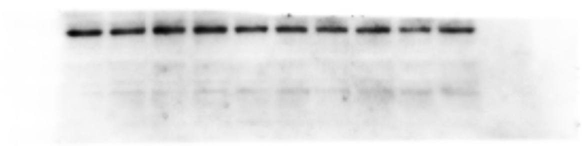

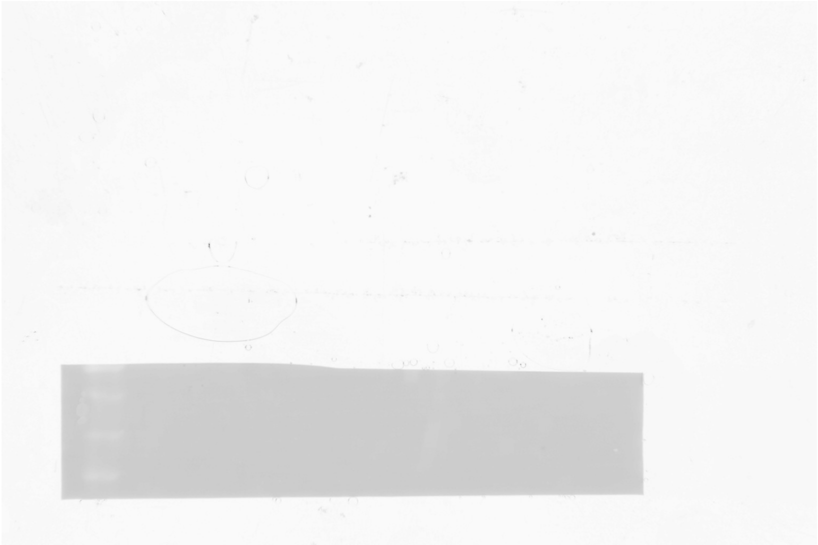

p-ERK

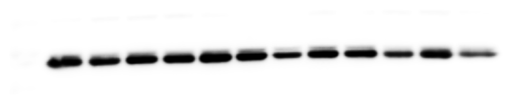

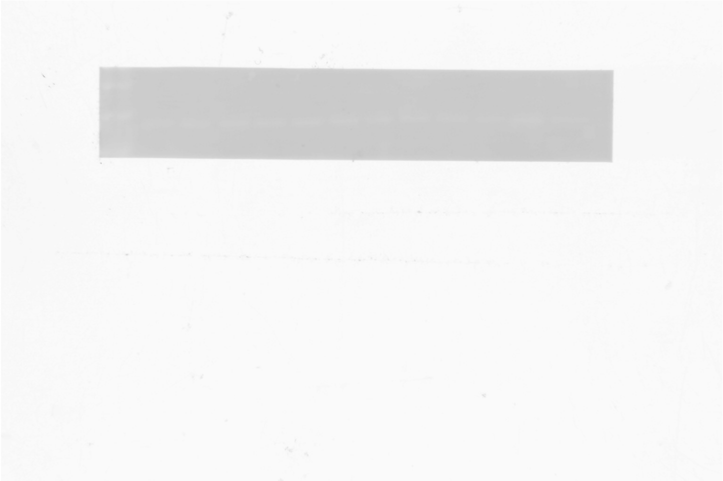

LC-3AB

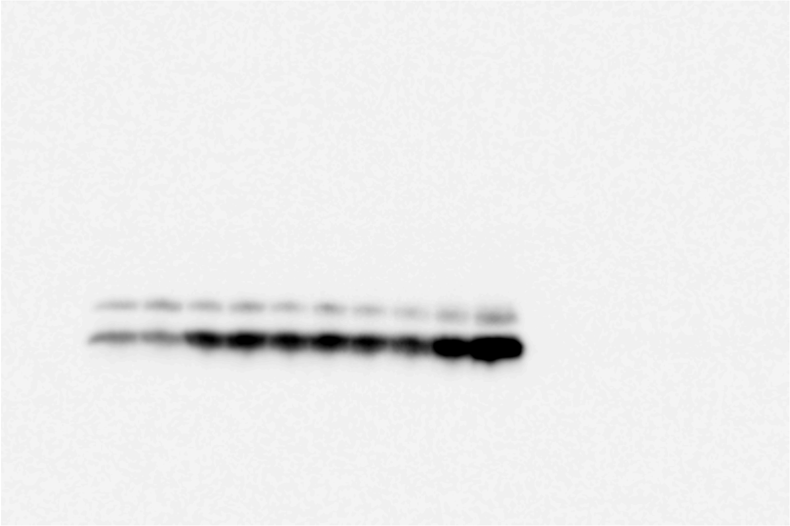

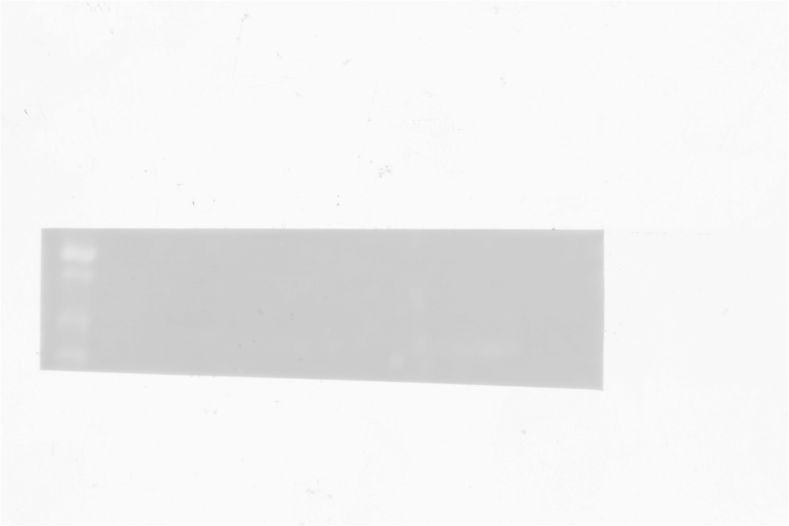

beta-actin

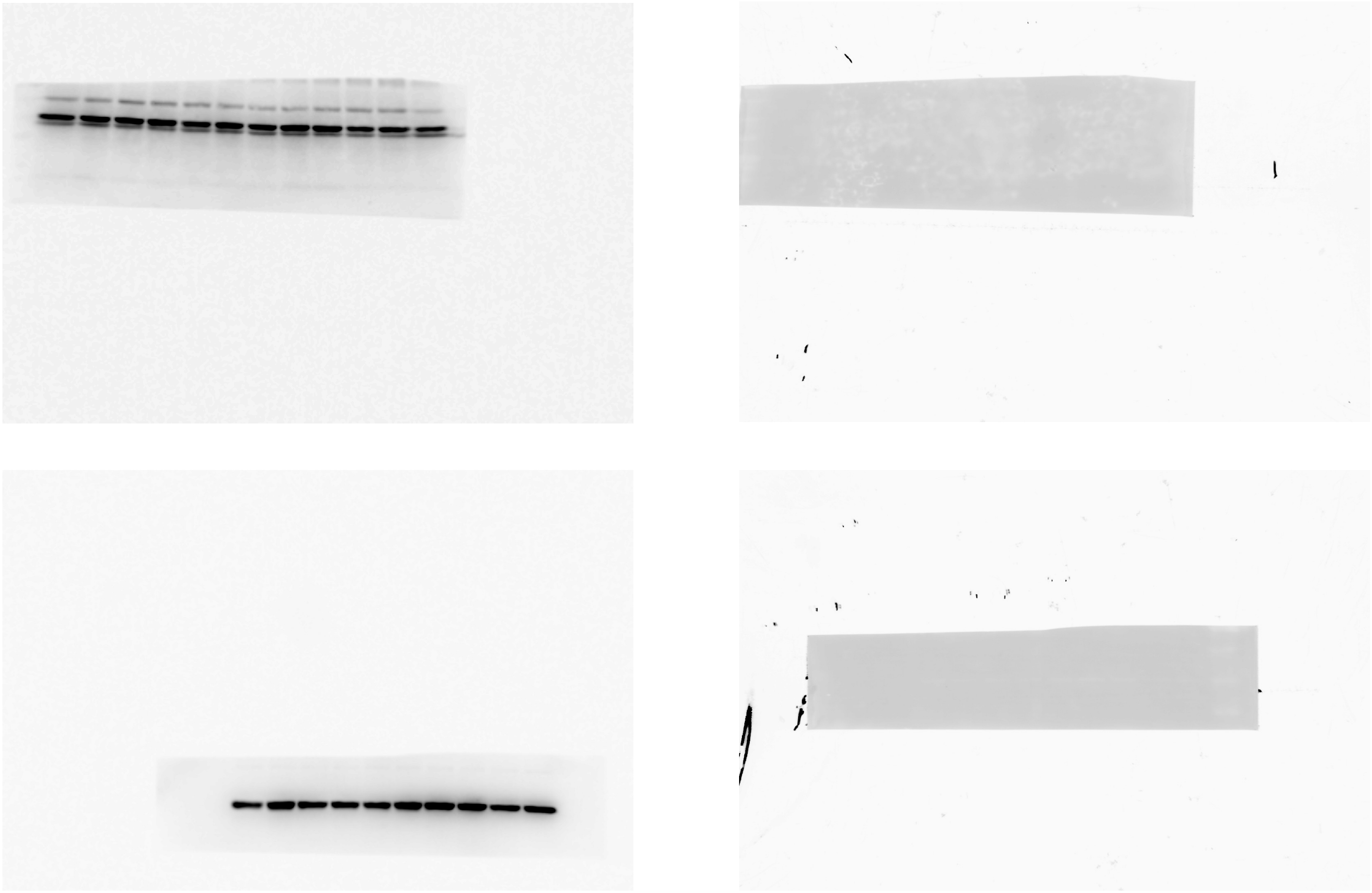

Fig.3E

p-IR

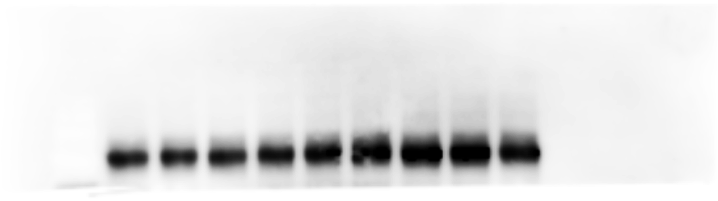

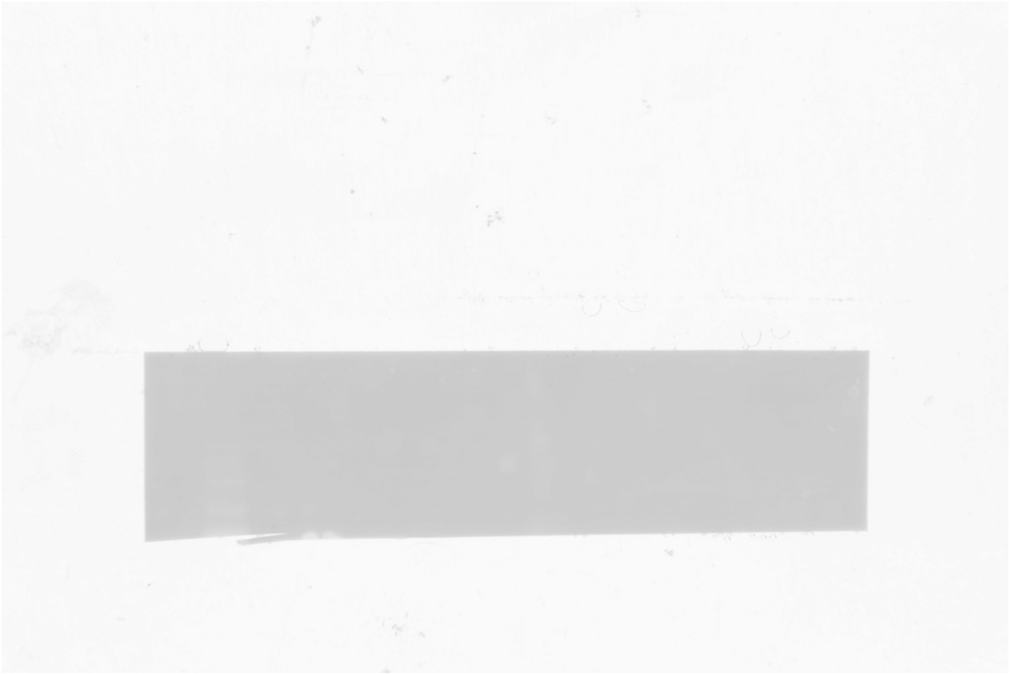

p-AKT473

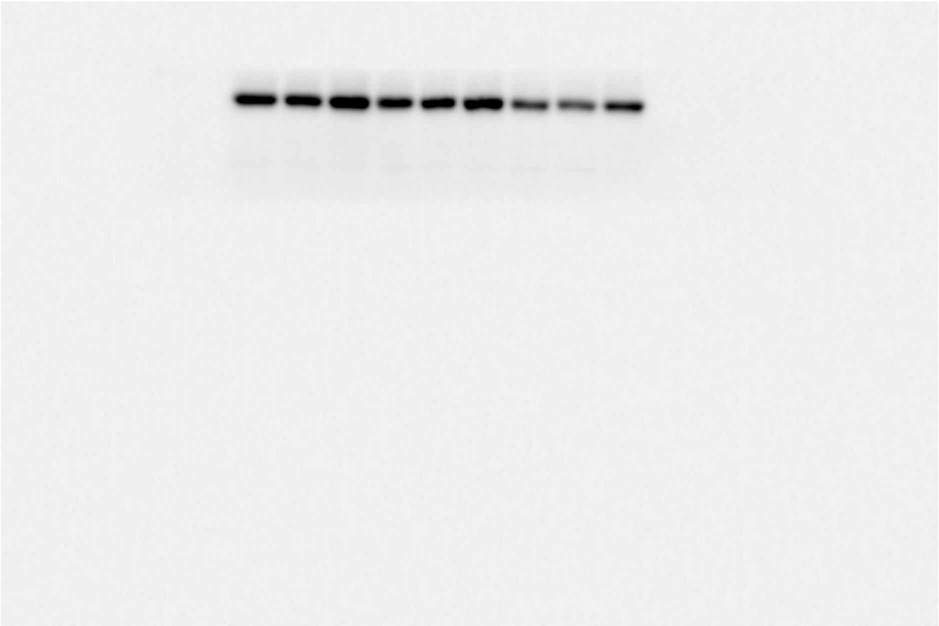

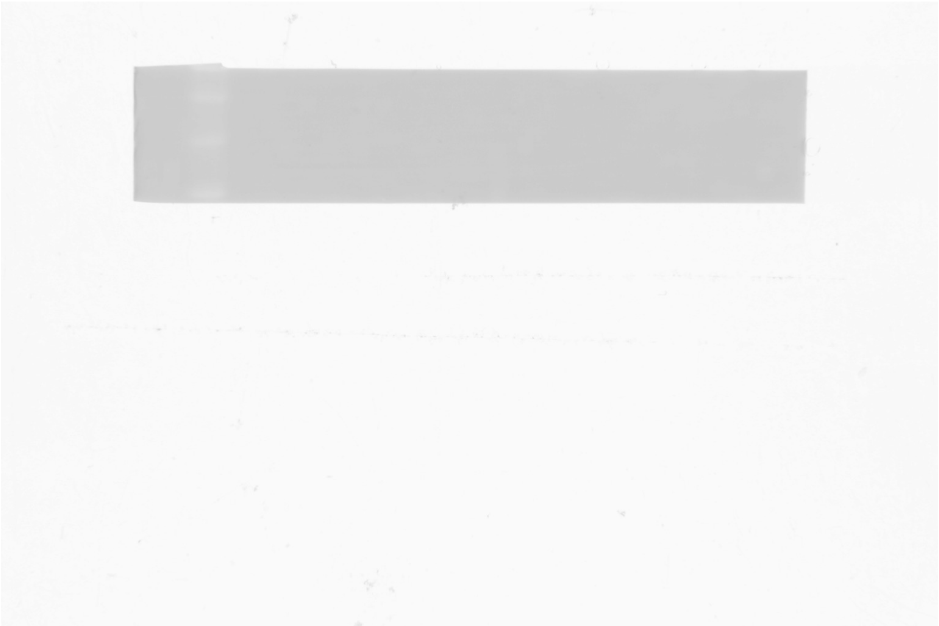

beta-actin

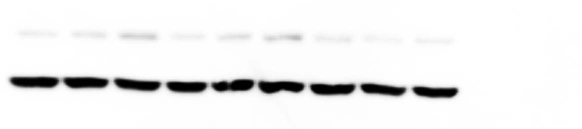

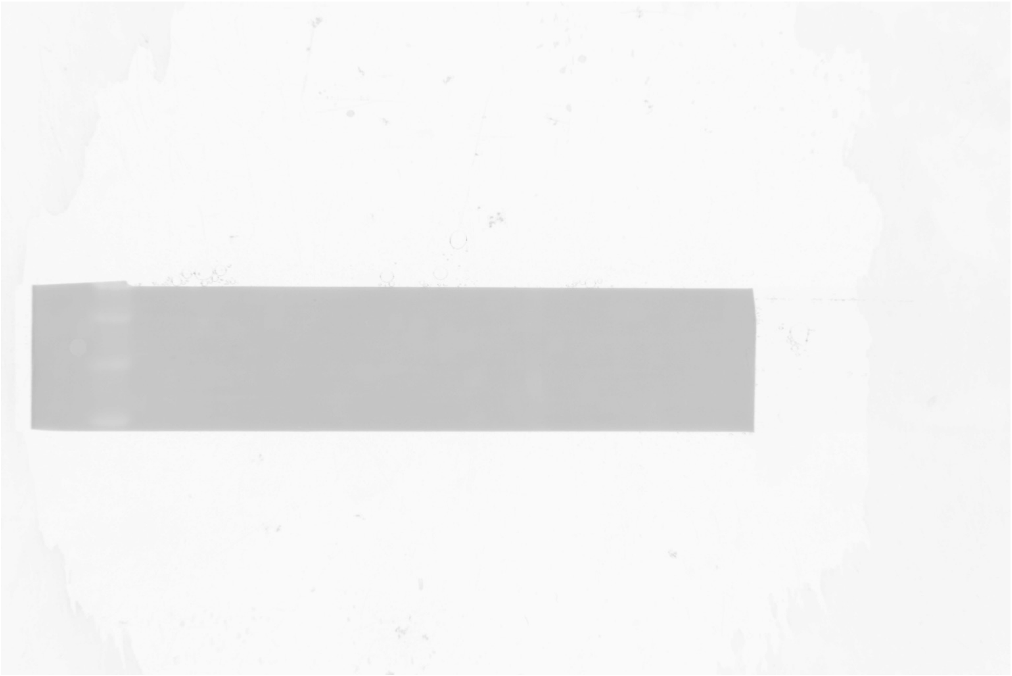

Fig.4G

p-IR

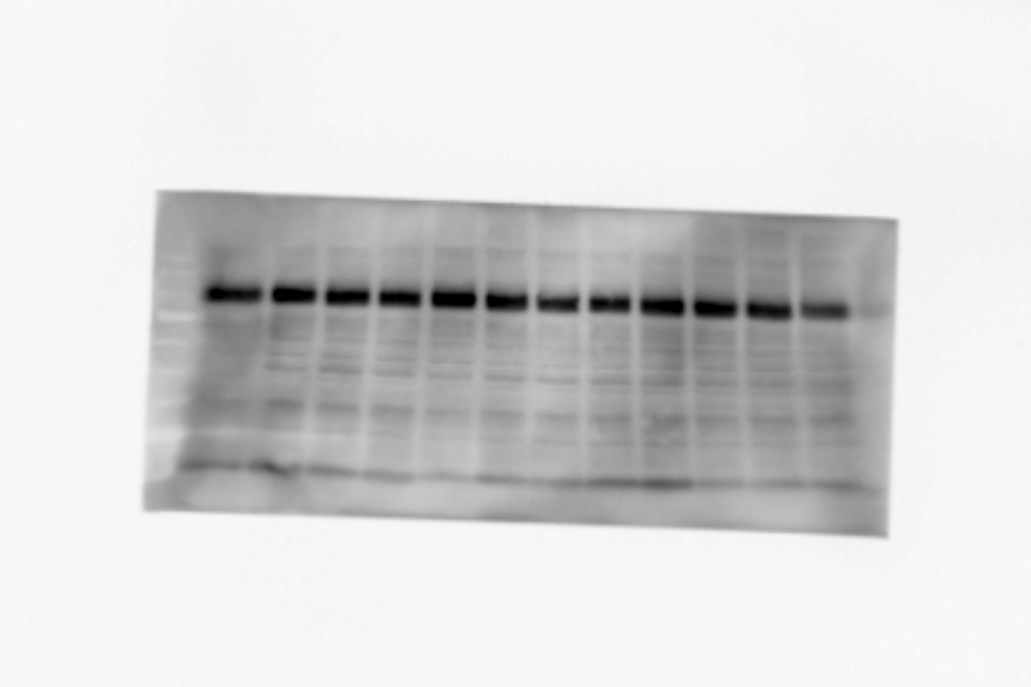

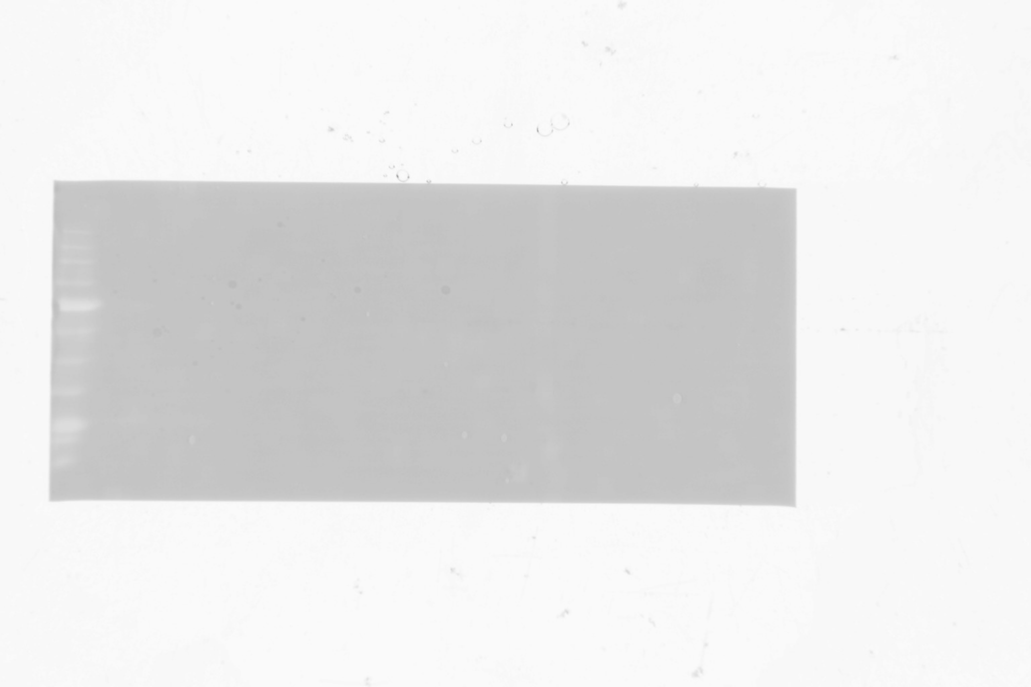

p-AKT473

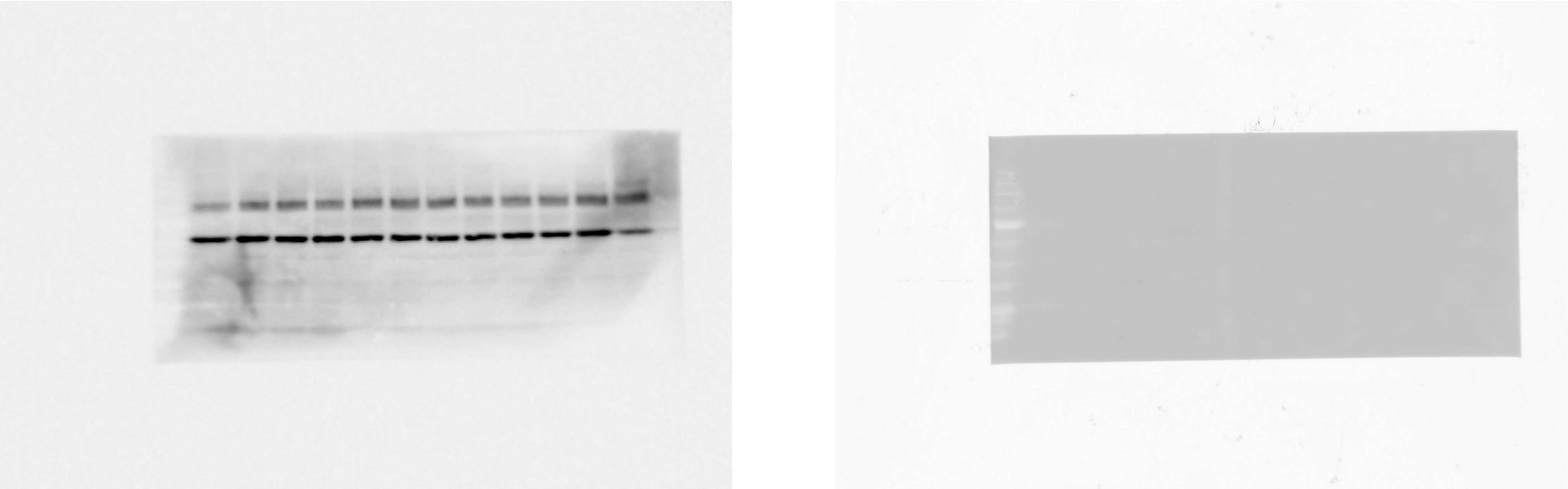

p-ERK

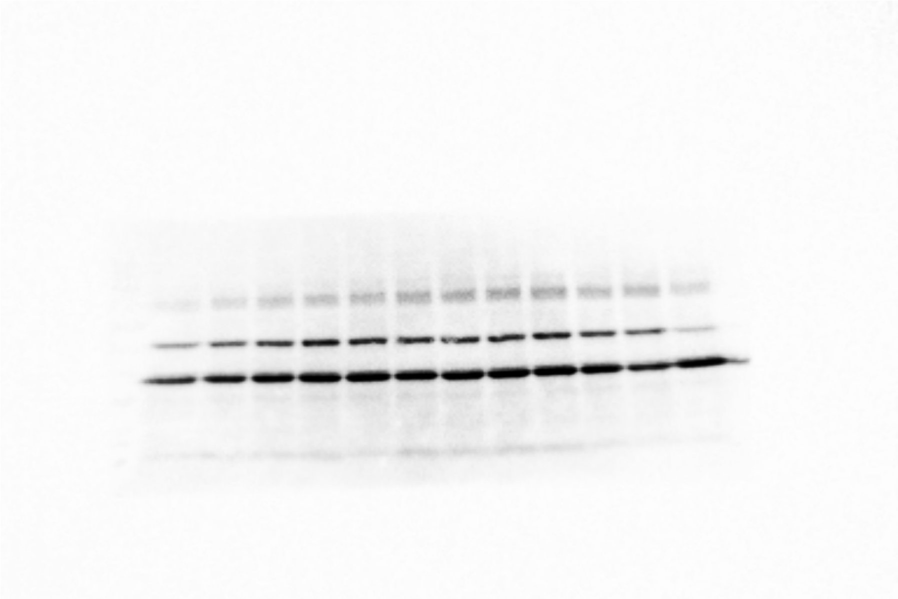

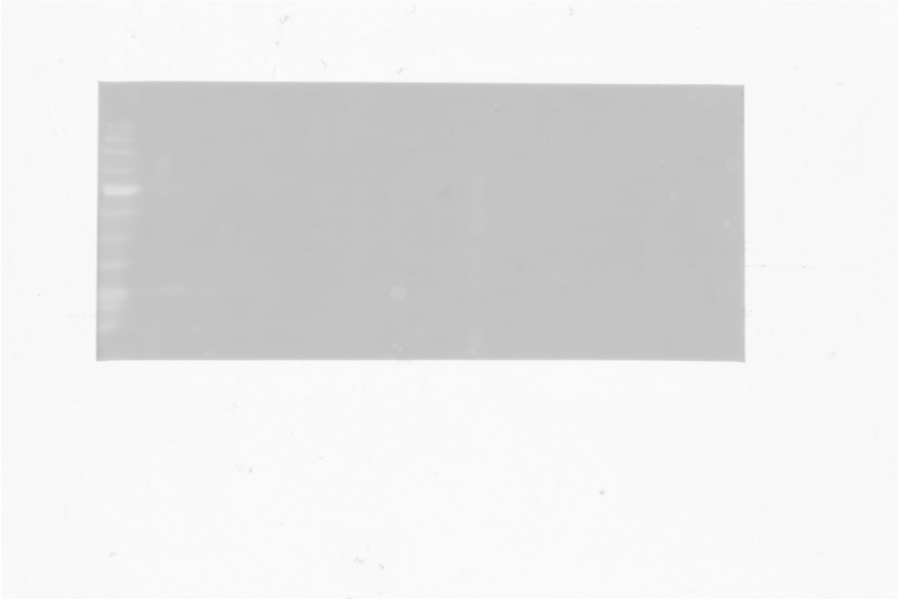

beta-actin

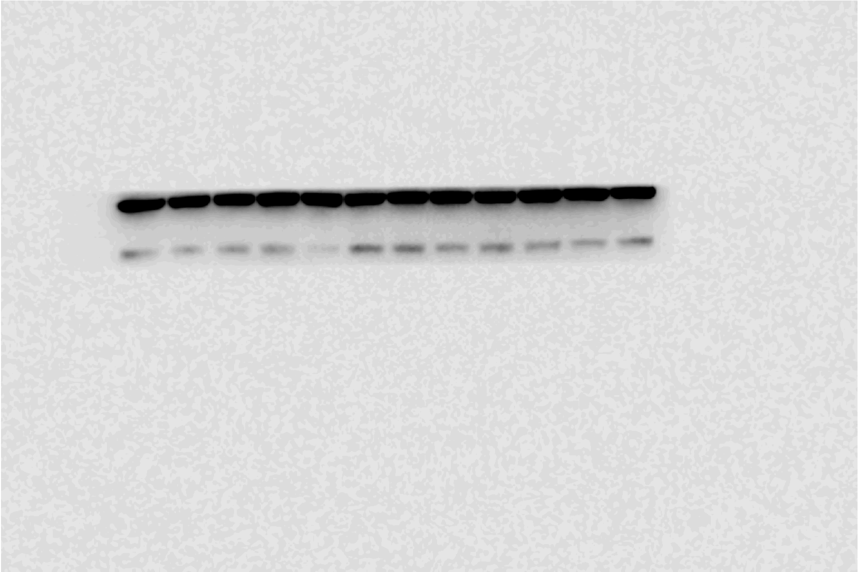

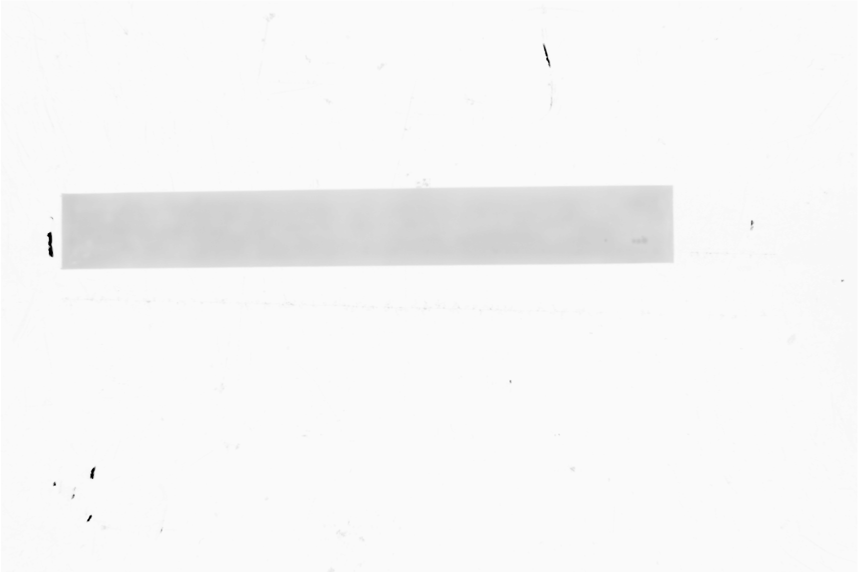

Fig.5B

p-mTOR

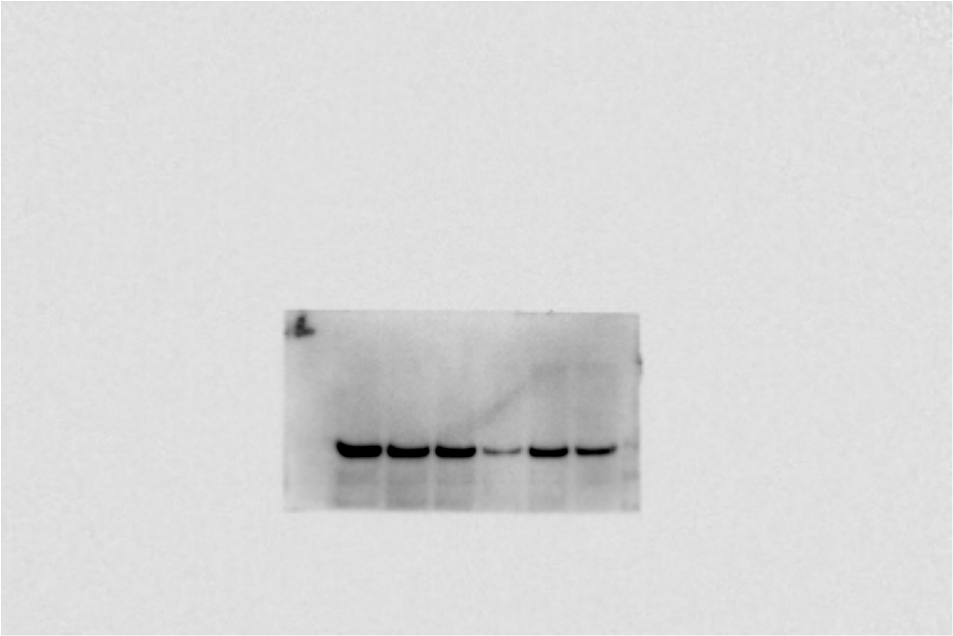

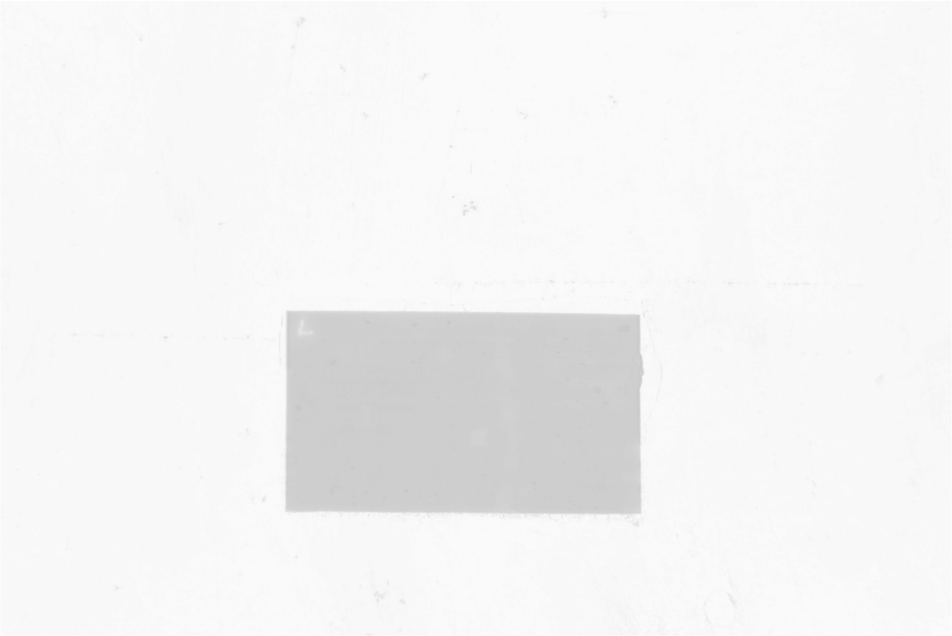

p-AMPK

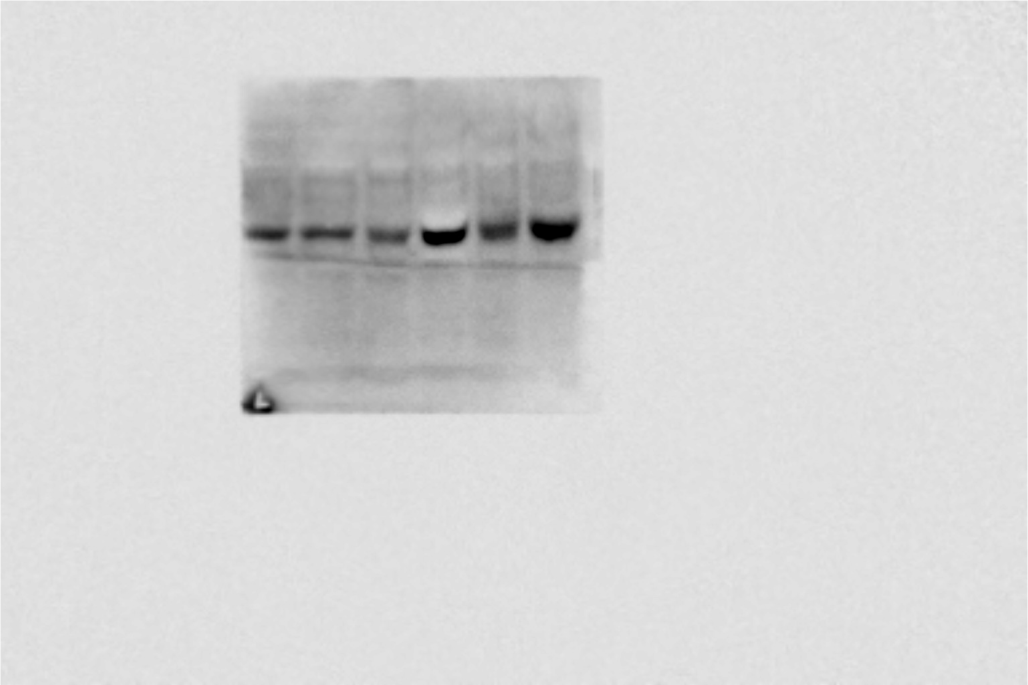

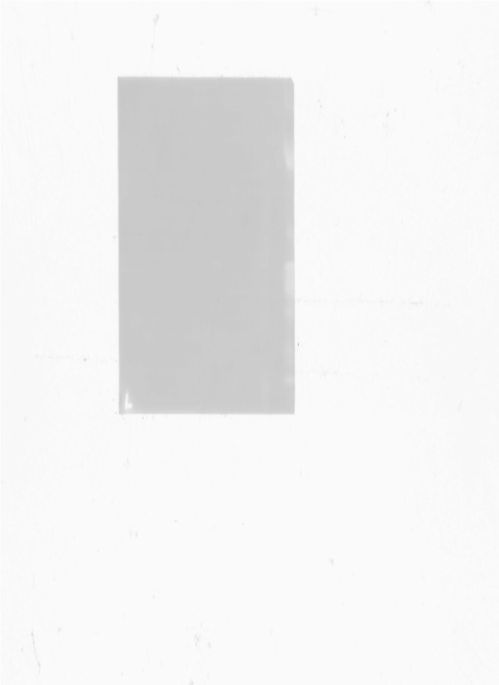

p-AKT308

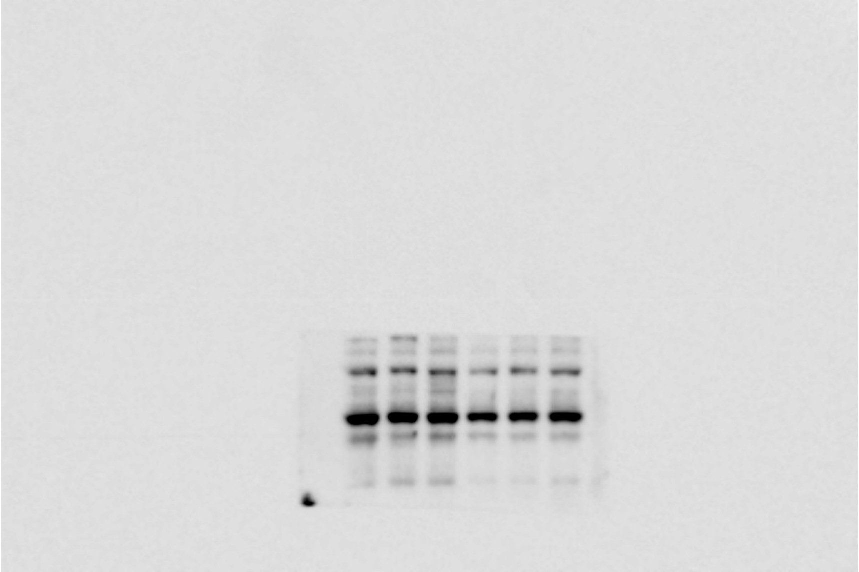

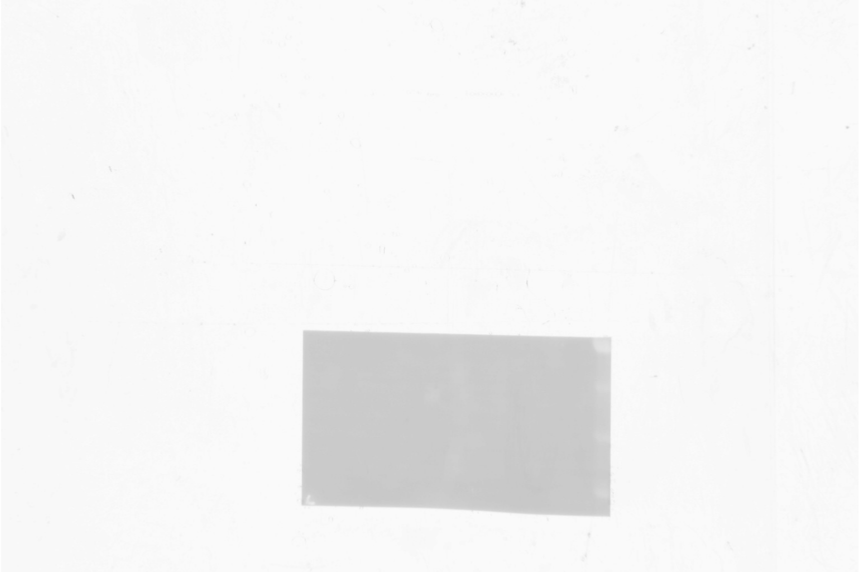

p-AKT473

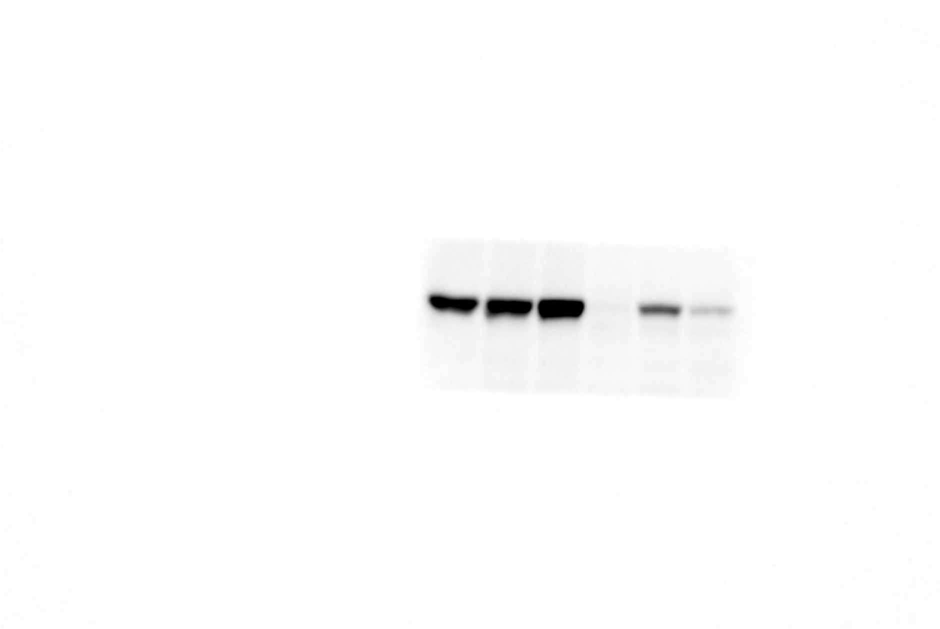

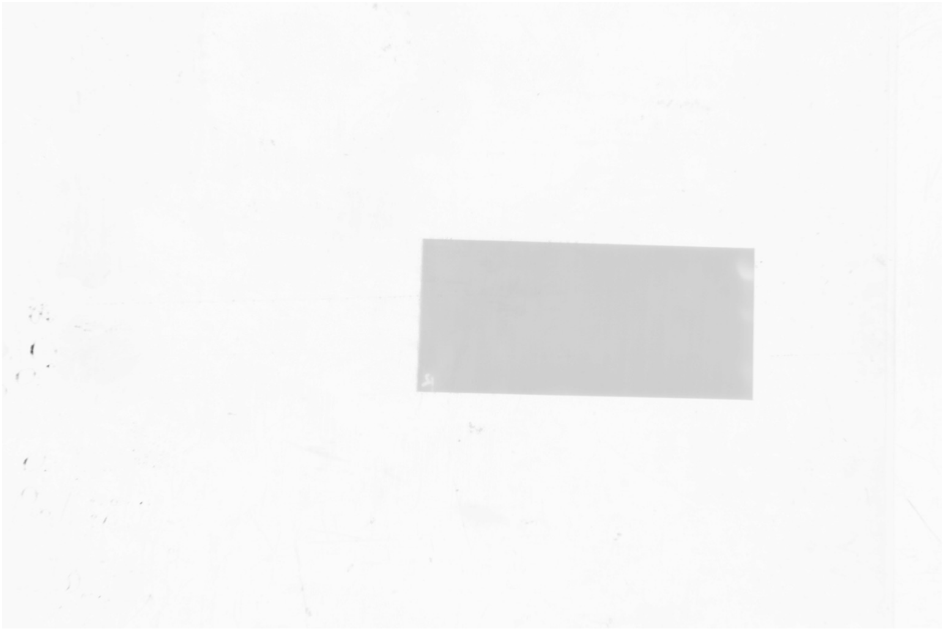

p-GSK

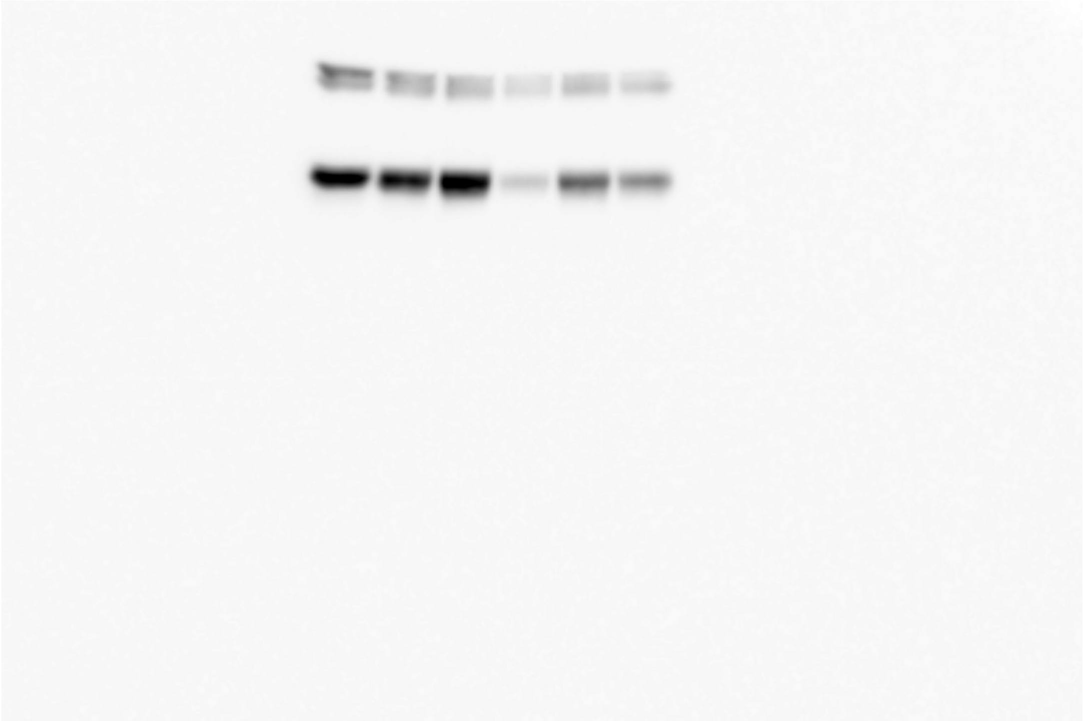

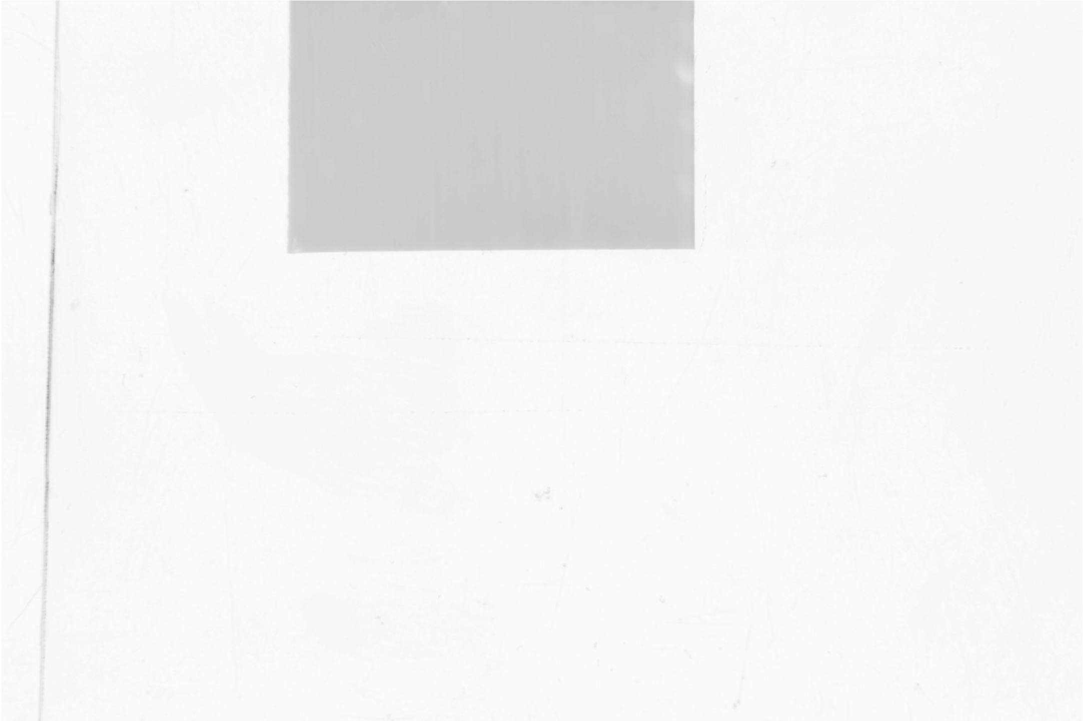

p-ERK

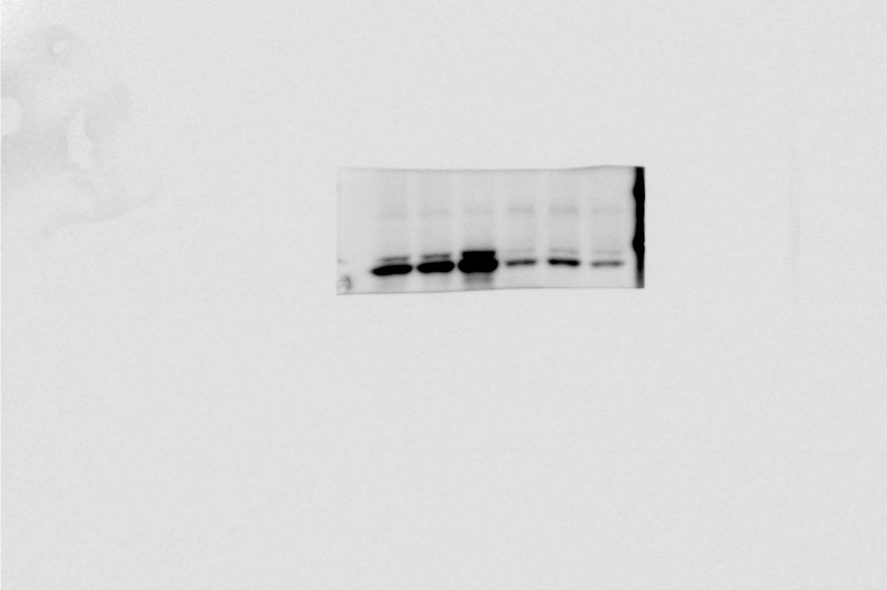

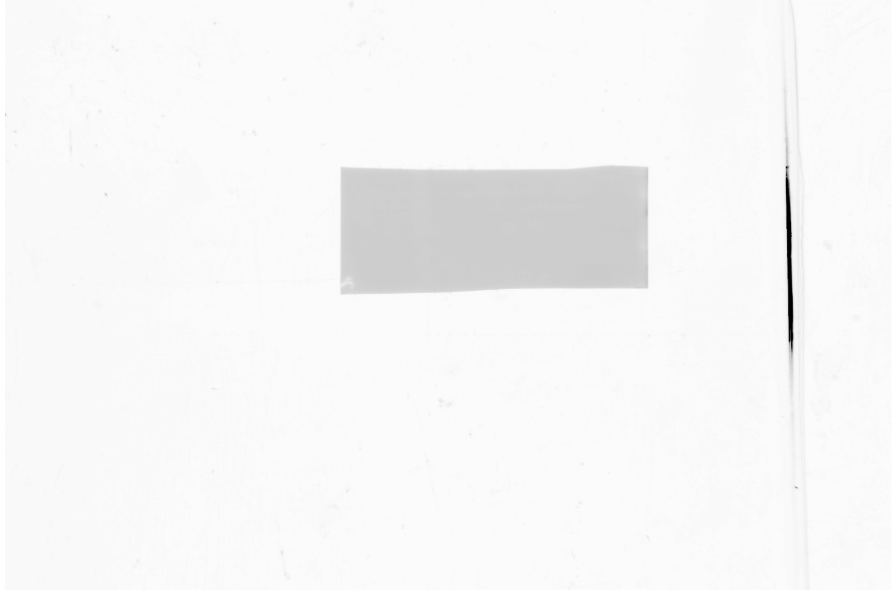

LC-3AB

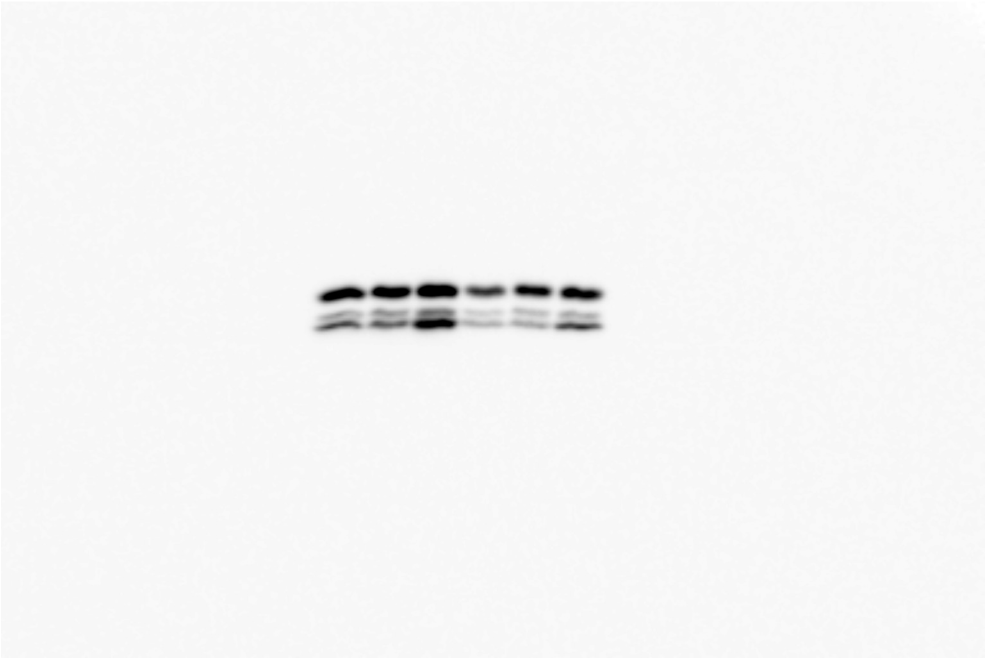

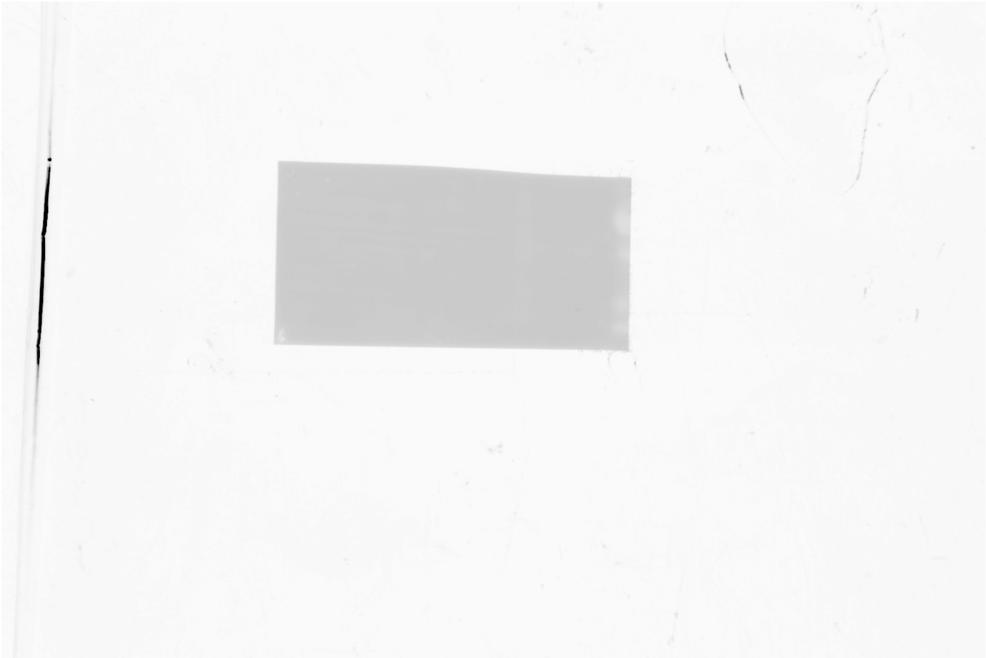

beta-actin

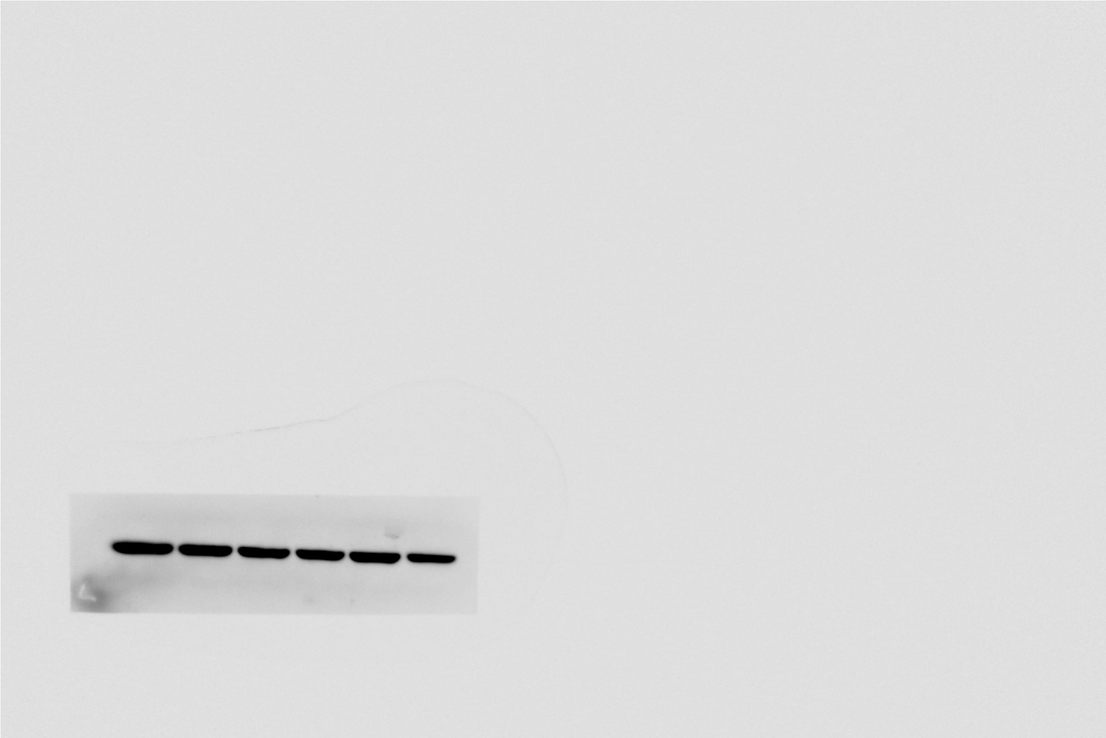

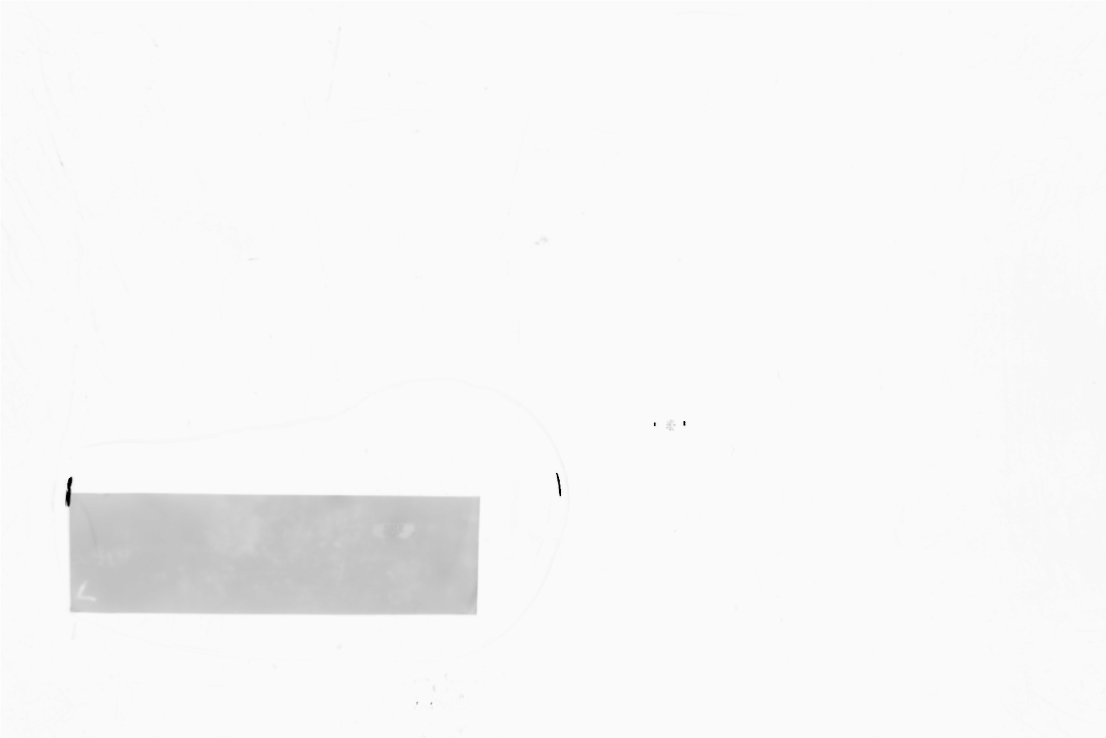

Supplement: Supplementary file 1 [file DataSheet1.PDF]
